# Supplementary material for: Effectiveness of a sepsis programme in a resource-limited setting: a retrospective analysis of data of a prospective observational study (Ubon-sepsis)
Source: BMJ Open. 2021 Feb 18;11(2):e041022. doi: 10.1136/bmjopen-2020-041022 (PMC7896572; doi:10.1136/bmjopen-2020-041022)
Supplement: Supplementary data [file bmjopen-2020-041022supp001.pdf]

Effectiveness of a sepsis programme in a resource-limited setting: a retrospective analysis of data of a prospective observational study (Ubon-sepsis)

Suchart Booraphun MD[1], Viriya Hantrakun PhD[2], Suwatthiya Siriboon MD[1], Chaiyaporn Boonsri MD[1], Pulyamon Poomthong MD[1], Bung-Orn Singkaew BNS[1], Oratai Wasombat BNS[1], Parinya Chamnan MD, PhD[1], Ratapum Champunot MD[3], Kristina Rudd MD, MPH[4, 5], Nicholas PJ. Day[2,6] MD[2], PhD, Arjen M. Dondorp MD, PhD[2,6], Prapit Teparrukkul MD[1], T. Eoin West MD, MPH[5,7], Direk Limmathurotsakul MD, PhD[2,6,8]

Short Title: Sepsis Fast Track Thailand

[1]Sunpasitthiprasong Hospital, Ubon Ratchathani, Thailand

[2]Mahidol Oxford Tropical Medicine Research Unit, Faculty of Tropical Medicine, Mahidol University, Bangkok, Thailand

[3]Department of Internal Medicine, Buddhachinaraj Phitsanulok Hospital, Phitsanulok, Thailand

[4]Department of Critical Care Medicine, University of Pittsburgh, Pittsburgh, Pennsylvania, USA

[5]Division of Pulmonary, Critical Care, and Sleep Medicine, University of Washington, Seattle, United States

[6]Centre for Tropical Medicine and Global Health, Nuffield Department of Medicine, University of Oxford, Churchill Hospital, Oxford, United Kingdom

[7]Department of Microbiology and Immunology, Faculty of Tropical Medicine, Mahidol University, Bangkok, Thailand

[8]Department of Tropical Hygiene, Faculty of Tropical Medicine, Mahidol University, Bangkok, Thailand

Supplementary Table 1 | Criteria used to systematically enroll patents into Sepsis Fast Track (SFT) upon admission

1. **Present with 2 or more of below Signs of systemic inflammatory response syndrome (SIRS)**
    - Body temperature > 38.3 °C or < 36.0 °C
    - Heart rate > 90 bpm
    - Respiratory rate > 20 pm or $PaCO_2$ < 32 mmHg
    - WBC > 12,000 /µL or < 4,000 /µL or Band forms > 10%
2. **Suspected sources of infection**
    - Pneumonia
    - Urinary track infection
    - Intra-abdominal infection
    - Skin and soft tissue infection
    - CNS infection
    - Others infections or unspecified source of infection
3. **Diagnostic criteria for severe sepsis:** patient met criteria in no 1 and 2 and has at least one of the following criteria
    - Mottled skin
    - Capillary refilling time ≥ 3 seconds
    - Urine output < 0.5 ml/kg/hour
    - Abrupt change in mental status
    - Acute respiratory failure
    - Platelet count < 100,000 /µL
    - Disseminated intravascular coagulation
    - Lactate > 2 mmol/L
    - SBP < 90 mmHg or MAP < 65 mmHg
4. **Diagnostic criteria for septic shock:** patient who are severe sepsis and has at least 1 of the following criteria
    - SBP < 90 mmHg or MAP < 65 mmHg after crystalliod administration ≥ 40-60 ml/kg of body weight OR after colloid administration ≥ 20-30 ml/kg of body weight
    - Require administration of dopamine > 5µg/kg of BW/min or norepinephrine/ epinephrine > 0.02 µg/kg of BW/min to maintain MAP to be > 65 mmHg

Booraphun S, *et al. BMJ Open* 2021; 11:e041022. doi: 10.1136/bmjopen-2020-041022

BMJ Publishing Group Limited (BMJ) disclaims all liability and responsibility arising from any reliance placed on this supplemental material which has been supplied by the author(s)

Supplementary Table 2 | Systemic manifestation of infection criteria used for enrollment in Ubon-Sepsis Cohort

General parameters

1. Fever or hypothermia (Core body temperature defined as > 38.3 °C or < 36.0 °C)
2. Tachycardia (heart rate > 90 beats per minute)
3. Tachypnea (respiratory rate > 20 per minute)
4. Altered mental status with Glasgow Coma Score (GCS) < 15 or <10 if intubated
5. Hyperglycemia (plasma glucose > 140 mg/dL) in the absence of diabetes

Inflammatory parameters

6. Leukocytosis (white blood cell count > 12,000/µL), leukopenia (white blood cell count < 4000/µL) or immature forms > 10%
7. Plasma C-reactive protein > 2 SD above the normal value
8. Plasma procalcitonin > 2 SD above the normal value

Hemodynamic parameters

9. Arterial hypotension (systolic blood pressure (SBP) < 90 mmHg, mean arterial pressure (MAP) < 70 mmHg, or SBP decrease > 40 mmHg)

Organ dysfunction parameters

10. Low oxygen saturation determined by pulse oximetry (SpO2 < 95%) determined by pulse oximetry
11. Arterial hypoxemia (PaO2 / FIO2 < 300)
12. Acute oliguria (urine output < 0.5 mL/kg/hr or 45 mmol/L for 2 hours)
13. Creatinine increase > 0.5 mg/dL
14. Coagulation abnormalities (international normalised ratio >1·5 or activated partial thromboplastin time >60 seconds)
15. Thrombocytopenia (Platelet count < 100,000 cells/µL)
16. Ileus (absent bowel sounds)
17. Hyperbilirubinaemia (plasma total bilirubin > 4 mg/dL)

Tissue perfusion parameters

18. Hyperlactatemia (> 1 mmol/L)
19. Decreased capillary refill or mottling
20. Significant edema or positive fluid balance

Booraphun S, *et al. BMJ Open* 2021; 11:e041022. doi: 10.1136/bmjopen-2020-041022

Supplementary Table 3 | Factors associated with 28-day mortality using multivariable Cox proportional hazards model

Variables	Died (n=779)	Survived (n=3027)	Adjusted hazard ratio (95%CI)	P value
**SFT group[1]**	205 (26%)	698 (23%)	0.72 (0.58-0.88)	0.001
**Male gender**	445 (57%)	698 (23%)	0.87 (0.75-1.01)	0.06
**Age group (years) (n [%])**				
• 18-40	59 (8%)	688 (23%)	1	<0.001
• >40-60	222 (29%)	930 (31%)	1.72 (1.28-2.30)	
• >60-70	159 (20%)	568 (19%)	2.10 (1.54-2.86)	
• >70	339 (44%)	841 (28%)	3.41 (2.57-4.53)	
**Transferred from other hospital**	715 (92%)	2595 (86%)	1.14 (0.88-1.49)	0.33
**Modified SOFA score (median, IQR)**	6 (4-9)	4 (3-6)	1.23 (1.21-1.26)	<0.001
**Comorbidities**				
• Diabetes mellitus	205 (26%)	602 (20%)	1.06 (0.90-1.26)	0.47
• Chronic kidney disease	141 (18%)	379 (13%)	1.22 (1.01-1.48)	0.04
• Liver disease	39 (5%)	85 (3%)	1.27 (0.91-1.76)	0.16
• Malignancy	24 (3%)	36 (1%)	2.64 (1.75-3.99)	<0.001
**Blood culture positive for pathogenic organisms**	190 (24%)	332 (11%)	1.83 (1.55-2.17)	<0.001
**Year**				
• 2013	165 (21%)	637 (21%)	1	0.30
• 2014	183 (23%)	651 (22%)	1.03 (0.83-1.27)	
• 2015	207 (27%)	808 (27%)	1.05 (0.84-1.31)	
• 2016[2]	224 (29%)	931 (31%)	0.88 (0.70-1.11)	
**Direct admission to the ICU**	128 (16%)	170 (6%)	1.68 (1.36-2.06)	<0.001

[1] Enrolled in the Sepsis Fast Track (SFT) programme
[2] Included 28 patients in 2017

Supplementary Table 4 | Factors associated with 28-day mortality using multivariable Cox proportional hazards model without a variable of the direct admission to the ICU

Variables	Died (n=779)	Survived (n=3027)	Adjusted hazard ratio (95%CI)	P value
**SFT group**[1]	205 (26%)	698 (23%)	0.77 (0.63-0.94)	0.01
**Male gender**	445 (57%)	698 (23%)	0.86 (0.74-1.00)	0.05
**Age group (years) (n [%])**				
• 18-40	59 (8%)	688 (23%)	1	<0.001
• >40-60	222 (29%)	930 (31%)	1.69 (1.26-2.26)	
• >60-70	159 (20%)	568 (19%)	2.07 (1.52-2.81)	
• >70	339 (44%)	841 (28%)	3.32 (2.50-4.41)	
**Transferred from other hospital**	715 (92%)	2595 (86%)	1.16 (0.89-1.52)	0.26
**Modified SOFA score (median, IQR)**	6 (4-9)	4 (3-6)	1.25 (1.22-1.27)	<0.001
**Comorbidities**				
• Diabetes mellitus	205 (26%)	602 (20%)	1.08 (0.91-1.27)	0.39
• Chronic kidney disease	141 (18%)	379 (13%)	1.20 (0.99-1.45)	0.07
• Liver disease	39 (5%)	85 (3%)	1.24 (0.89-1.72)	0.20
• Malignancy	24 (3%)	36 (1%)	2.52 (1.67-3.81)	<0.001
**Blood culture positive for pathogenic organisms**	190 (24%)	332 (11%)	1.83 (1.54-2.16)	<0.001
**Year**				
• 2013	165 (21%)	637 (21%)	1	0.34
• 2014	183 (23%)	651 (22%)	0.98 (0.79-1.21)	
• 2015	207 (27%)	808 (27%)	1.01 (0.81-1.26)	
• 2016[2]	224 (29%)	931 (31%)	0.85 (0.68-1.07)	

[1] Enrolled in the Sepsis Fast Track (SFT) programme
[2] Included 28 patients in 2017

Supplementary Table 5 | Factors associated with 28-day mortality using logistic multivariable model

Variables	Died (n=779)	Survived (n=3027)	Adjusted odds ratio (95%CI)	P value
**SFT group[1]**	205 (26%)	698 (23%)	0.61 (0.48-0.79)	<0.001
**Male gender**	445 (57%)	698 (23%)	0.88 (0.73-1.06)	0.18
**Age group (years) (n [%])**				
• 18-40	59 (8%)	688 (23%)	1	<0.001
• >40-60	222 (29%)	930 (31%)	1.91 (1.36-2.66)	
• >60-70	159 (20%)	568 (19%)	2.54 (1.78-3.63)	
• >70	339 (44%)	841 (28%)	4.76 (3.43-6.59)	
**Transferred from other hospital**	715 (92%)	2595 (86%)	1.21 (0.89-1.65)	0.23
**Modified SOFA score (median, IQR)**	6 (4-9)	4 (3-6)	1.30 (1.26-1.34)	<0.001
**Comorbidities**				
• Diabetes mellitus	205 (26%)	602 (20%)	1.12 (0.91-1.39)	0.29
• Chronic kidney disease	141 (18%)	379 (13%)	1.20 (0.94-1.53)	0.13
• Liver disease	39 (5%)	85 (3%)	1.34 (0.85-2.10)	0.21
• Malignancy	24 (3%)	36 (1%)	3.65 (2.05-6.51)	<0.001
**Blood culture positive for pathogenic organisms**	190 (24%)	332 (11%)	2.08 (1.66-2.61)	<0.001
**Year**				
• 2013	165 (21%)	637 (21%)	1	0.64
• 2014	183 (23%)	651 (22%)	1.05 (0.80-1.37)	
• 2015	207 (27%)	808 (27%)	1.05 (0.80-1.38)	
• 2016[2]	224 (29%)	931 (31%)	0.91 (0.69-1.21)	
**Admitted directly to an ICU upon admission**	128 (16%)	170 (6%)	1.95 (1.45-2.62)	<0.001

[1] Enrolled in the Sepsis Fast Track (SFT) programme
[2] Included 28 patients in 2017

Booraphun S, *et al. BMJ Open* 2021; 11:e041022. doi: 10.1136/bmjopen-2020-041022

Supplementary Table 6 | Factors associated with 28-day mortality using logistic multivariable model without a variable of the direct admission to the ICU

Variables	Died (n=779)	Survived (n=3027)	Adjusted odds ratio (95%CI)	P value
**SFT group[1]**	205 (26%)	698 (23%)	0.67 (0.53-0.86)	0.002
**Male gender**	445 (57%)	698 (23%)	0.87 (0.73-1.05)	0.14
**Age group (years) (n [%])**				
• 18-40	59 (8%)	688 (23%)	1	<0.001
• >40-60	222 (29%)	930 (31%)	1.85 (1.33-2.58)	
• >60-70	159 (20%)	568 (19%)	2.44 (1.71-3.46)	
• >70	339 (44%)	841 (28%)	4.55 (3.29-6.28)	
**Transferred from other hospital**	715 (92%)	2595 (86%)	1.23 (0.90-1.68)	0.19
**Modified SOFA score (median, IQR)**	6 (4-9)	4 (3-6)	1.32 (1.28-1.36)	<0.001
**Comorbidities**				
• Diabetes mellitus	205 (26%)	602 (20%)	1.16 (0.94-1.43)	0.17
• Chronic kidney disease	141 (18%)	379 (13%)	1.17 (0.92-1.49)	0.20
• Liver disease	39 (5%)	85 (3%)	1.31 (0.84-2.06)	0.23
• Malignancy	24 (3%)	36 (1%)	3.49 (1.95-6.24)	<0.001
**Blood culture positive for pathogenic organisms**	190 (24%)	332 (11%)	2.08 (1.67-2.61)	<0.001
**Year**				
• 2013	165 (21%)	637 (21%)	1	0.70
• 2014	183 (23%)	651 (22%)	1.00 (0.77-1.31)	
• 2015	207 (27%)	808 (27%)	1.01 (0.77-1.33)	
• 2016[2]	224 (29%)	931 (31%)	0.89 (0.67-1.17)	

[1] Enrolled in the Sepsis Fast Track (SFT) programme
[2] Included 28 patients in 2017

Booraphun S, *et al. BMJ Open* 2021; 11:e041022. doi: 10.1136/bmjopen-2020-041022

Supplementary Table 7 | Factors associated with 28-day mortality using multivariable Cox proportional hazards model in 2,170 patients enrolled into the study after the implementation of the Sepsis Fast Track (SFT) programme

Variables	Died (n=431)	Survived (n=1739)	Adjusted hazard ratio (95%CI)	P value
**SFT group**	205 (48%)	698 (40%)	0.68 (0.55-0.84)	<0.001
**Male gender**	254(59%)	992 (57%)	0.89 (0.73-1.09)	0.26
**Age group (years) (n [%])**				
• 18-40	30 (7%)	363(21%)	1	<0.001
• >40-60	125 (29%)	502 (29%)	1.97 (1.31-2.95)	
• >60-70	85 (20%)	359 (21%)	1.97 (1.28-3.03)	
• >70	191 (44%)	515 (30%)	3.33 (2.24-4.95)	
**Transferred from other hospital**	406 (94%)	1513 (87%)	1.49 (0.99-2.26)	0.06
**Modified SOFA score (median, IQR)**	7 (5-10)	4 (3-6)	1.24 (1.21-1.28)	<0.001
**Comorbidities**				
• Diabetes mellitus	122 (28%)	377(22%)	1.08 (0.87-1.35)	0.49
• Chronic kidney disease	84 (19%)	242 (14%)	1.23 (0.96-1.58)	0.11
• Liver disease	22 (5%)	57 (3%)	1.10 (0.71-1.70)	0.68
• Malignancy	19 (4%)	27 (2%)	2.90 (1.81-4.63)	<0.001
**Blood culture positive for pathogenic organisms**	110(26%)	225 (13%)	1.64 (1.31-2.05)	<0.001
**Year**				
• 2015	207 (48%)	808 (46%)	1	0.08
• 2016	224 (52%)	931 (54%)	0.84 (0.69-1.02)	
**Direct admission to the ICU**	85 (20%)	124 (7%)	1.78 (1.37-2.32)	<0.001

Booraphun S, *et al. BMJ Open* 2021; 11:e041022. doi: 10.1136/bmjopen-2020-041022

Supplementary Figure 1 | Preprinted recommended doctor orders for sepsis fast track programme used at the Emergency Department at Sunpasitthiprasong Hospital from 1 January 2015

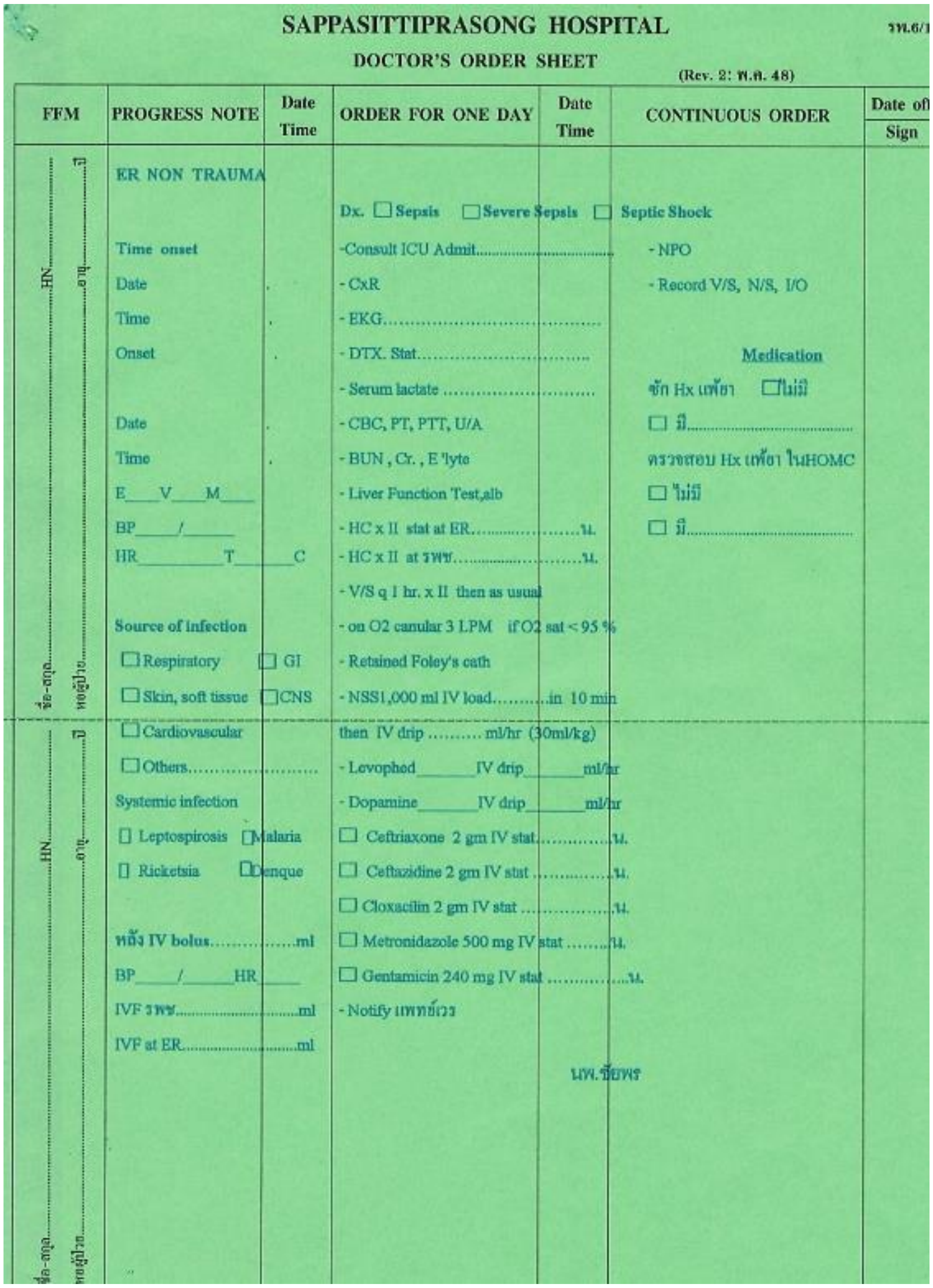

SAPPASITTIPRASONG HOSPITAL

รพ.6/1

DOCTOR'S ORDER SHEET

(Rev. 2: พ.ค. 48)

FFM	PROGRESS NOTE	Date Time	ORDER FOR ONE DAY	Date Time	CONTINUOUS ORDER	Date off Sign

HN.......... อายุ..........ปี ชื่อ-สกุล.......... หอผู้ป่วย..........

PROGRESS NOTE

ER NON TRAUMA

Time onset
Date .
Time .
Onset .

Date .
Time .
E___V___M___
BP____/____
HR_____T____C

Source of infection
☐ Respiratory ☐ GI
☐ Skin, soft tissue ☐ CNS
☐ Cardiovascular
☐ Others..........

Systemic infection
☐ Leptospirosis ☐ Malaria
☐ Ricketsia ☐ Denque

หลัง IV bolus..........ml
BP____/____HR____
IVF รพช..........ml
IVF at ER..........ml

ORDER FOR ONE DAY

Dx. ☐ Sepsis ☐ Severe Sepsis ☐ Septic Shock

-Consult ICU Admit..........
- CxR
- EKG..........
- DTX. Stat..........
- Serum lactate..........
- CBC, PT, PTT, U/A
- BUN , Cr. , E'lyte
- Liver Function Test,alb
- HC x II stat at ER..........น.
- HC x II at รพช..........น.
- V/S q 1 hr. x II then as usual
- on O2 canular 3 LPM if O2 sat < 95 %
- Retained Foley's cath
- NSS1,000 ml IV load..........in 10 min
then IV drip.......... ml/hr (30ml/kg)
- Levophed____IV drip____ml/hr
- Dopamine____IV drip____ml/hr
☐ Ceftriaxone 2 gm IV stat..........น.
☐ Ceftazidine 2 gm IV stat..........น.
☐ Cloxacilin 2 gm IV stat..........น.
☐ Metronidazole 500 mg IV stat..........น.
☐ Gentamicin 240 mg IV stat..........น.
- Notify แพทย์เวร

นพ.ชัยพร

CONTINUOUS ORDER

- NPO
- Record V/S, N/S, I/O

Medication
ซัก Hx แพ้ยา ☐ ไม่มี
☐ มี..........
ตรวจสอบ Hx แพ้ยา ในHOMC
☐ ไม่มี
☐ มี..........

Booraphun S, *et al. BMJ Open* 2021; 11:e041022. doi: 10.1136/bmjopen-2020-041022

Supplementary Figure 2 | The Sepsis Fast Track sepsis resuscitation workflow used at the Emergency Department at Sunpasitthiprasong Hospital from 1 March 2016

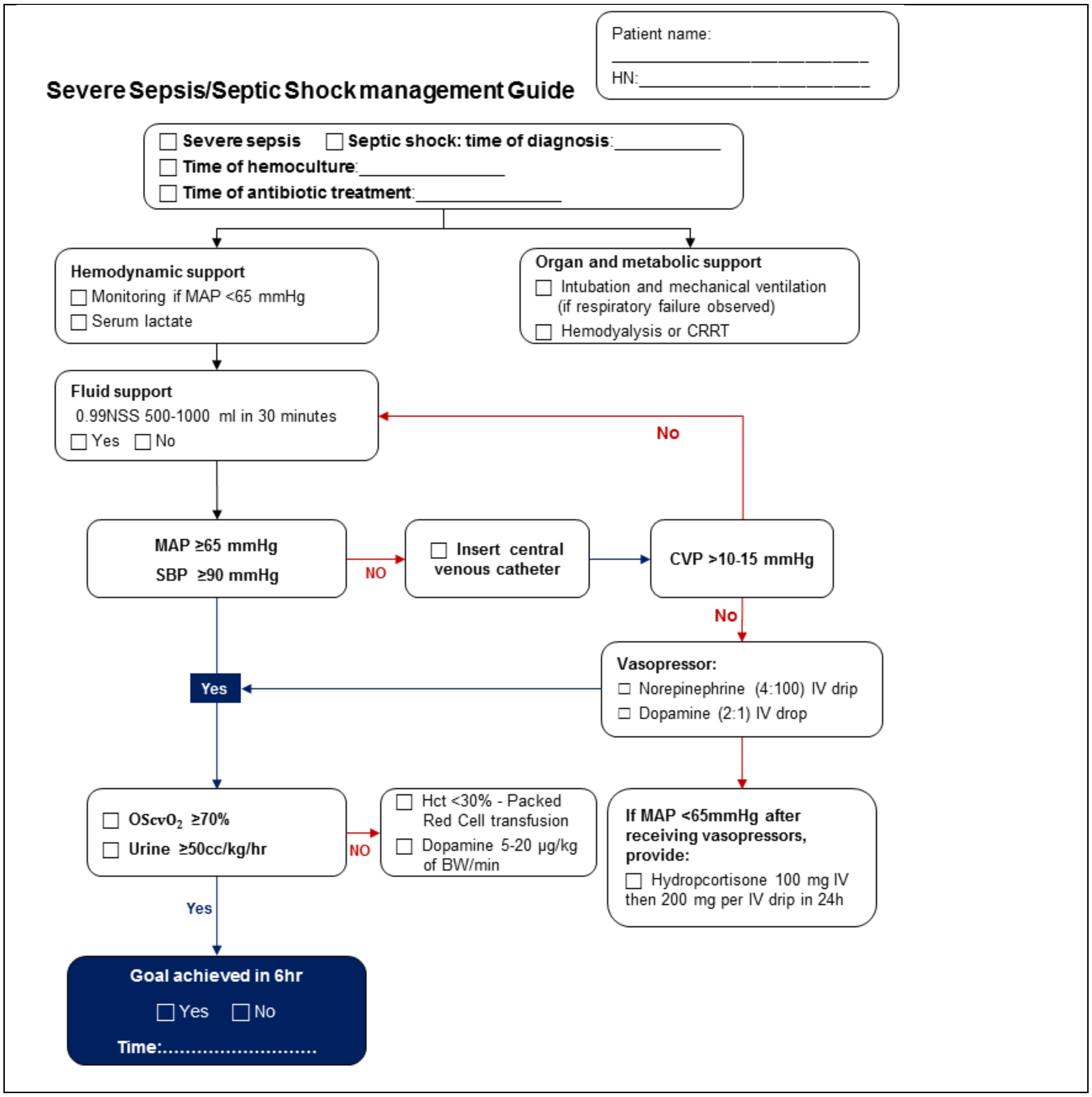

Booraphun S, *et al. BMJ Open* 2021; 11:e041022. doi: 10.1136/bmjopen-2020-041022
