# Supplementary material for: Effectiveness of a sepsis programme in a resource-limited setting: a retrospective analysis of data of a prospective observational study (Ubon-sepsis)
Source: BMJ Open. 2021 Feb 18;11(2):e041022. doi: 10.1136/bmjopen-2020-041022 (PMC7896572; doi:10.1136/bmjopen-2020-041022)
Supplement: Author's manuscript [file bmjopen-2020-041022.draft_revisions.pdf]

BMJ Open

BMJ Open is committed to open peer review. As part of this commitment we make the peer review history of every article we publish publicly available.

When an article is published we post the peer reviewers' comments and the authors' responses online. We also post the versions of the paper that were used during peer review. These are the versions that the peer review comments apply to.

The versions of the paper that follow are the versions that were submitted during the peer review process. They are not the versions of record or the final published versions. They should not be cited or distributed as the published version of this manuscript.

BMJ Open is an open access journal and the full, final, typeset and author-corrected version of record of the manuscript is available on our site with no access controls, subscription charges or pay-per-view fees (http://bmjopen.bmj.com).

If you have any questions on BMJ Open's open peer review process please email info.bmjopen@bmj.com

BMJ Open

Effectiveness of a sepsis programme implemented in a resource-limited setting: a natural experiment

Journal:	*BMJ Open*
Manuscript ID	bmjopen-2020-041022
Article Type:	Original research
Date Submitted by the Author:	28-May-2020
Complete List of Authors:	Booraphun, Suchart; Sunpasithiprasong Hospital, Medical Department<br>Hantrakun, Viriya; Faculty of Tropical Medicine, Microbiology<br>Siriboon, Suwatthiya; Sunpasithiprasong Hospital<br>Boonsri, Chaiyaporn ; Sunpasithiprasong Hospital, Emergency Department<br>Poomthong, Pulyamon; Sunpasithiprasong Hospital<br>Singkaew, Bung-Orn; Sunpasithiprasong Hospital<br>Wasombat, Oratai; Sunpasithiprasong Hospital<br>Chamnan, Parinya; Sunpasithiprasong Hospital<br>Champunot, Ratapum; Buddhachinaraj Phitsanulok Hospital, Department of Internal Medicine<br>Rudd, Kristina; University of Pittsburgh, Department of Critical Care Medicine<br>Day, Nicholas; Mahidol-Oxford Tropical Medicine Research Unit; University of Oxford, Centre for Tropical Medicine, Nuffield Department of Clinical Medicine<br>Dondorp, Arjen; Mahidol University, Mahidol-Oxford Tropical Medicine Research Unit (MORU), Faculty of Tropical Medicine; University of Oxford, Centre for Tropical Medicine, Nuffield Department of Medicine<br>Teparrukkul, Prapit; Sappasithiprasong Hospital<br>West, Eoin; University of Washington<br>Limmathurotsakul, Direk; Faculty of Tropical Medicine
Keywords:	Epidemiology < INFECTIOUS DISEASES, INFECTIOUS DISEASES, INTENSIVE & CRITICAL CARE

SCHOLARONE™
Manuscripts

BMJ

I, the Submitting Author has the right to grant and does grant on behalf of all authors of the Work (as defined in the below author licence), an exclusive licence and/or a non-exclusive licence for contributions from authors who are: i) UK Crown employees; ii) where BMJ has agreed a CC-BY licence shall apply, and/or iii) in accordance with the terms applicable for US Federal Government officers or employees acting as part of their official duties; on a worldwide, perpetual, irrevocable, royalty-free basis to BMJ Publishing Group Ltd ("BMJ") its licensees and where the relevant Journal is co-owned by BMJ to the co-owners of the Journal, to publish the Work in this journal and any other BMJ products and to exploit all rights, as set out in our licence.

The Submitting Author accepts and understands that any supply made under these terms is made by BMJ to the Submitting Author unless you are acting as an employee on behalf of your employer or a postgraduate student of an affiliated institution which is paying any applicable article publishing charge ("APC") for Open Access articles. Where the Submitting Author wishes to make the Work available on an Open Access basis (and intends to pay the relevant APC), the terms of reuse of such Open Access shall be governed by a Creative Commons licence – details of these licences and which Creative Commons licence will apply to this Work are set out in our licence referred to above.

Other than as permitted in any relevant BMJ Author's Self Archiving Policies, I confirm this Work has not been accepted for publication elsewhere, is not being considered for publication elsewhere and does not duplicate material already published. I confirm all authors consent to publication of this Work and authorise the granting of this licence.

For peer review only

Effectiveness of a sepsis programme implemented in a resource-limited setting: a natural experiment

Suchart Booraphun MD[1], Viriya Hantrakun PhD[2], Suwatthiya Siriboon MD[1], Chaiyaporn Boonsri MD[1], Pulyamon Poomthong MD[1], Bung-Orn Singkaew BNS[1], Oratai Wasombat BNS[1], Parinya Chamna MD, PhD[1], Ratapum Champunot MD[3], Kristina Rudd MD, MPH[4, 5], Nicholas PJ. Day[2,6] MD[2], PhD, Arjen Dondrop MD[2,6], PhD, Prapit Teparrukkul MD[1], T. Eoin West MD, MPH[5], Direk Limmathurotsakul MD, PhD[2,6,7]

Short Title: Sepsis Fast Track Thailand

[1]Sunpasitthiprasong Hospital, Ubon Ratchathani, Thailand

[2]Mahidol Oxford Tropical Medicine Research Unit, Faculty of Tropical Medicine, Mahidol University, Bangkok, Thailand

[3]Department of Internal Medicine, Buddhachinaraj Phitsanulok Hospital, Phitsanulok, Thailand

[4]Department of Critical Care Medicine, University of Pittsburgh, Pittsburgh, Pennsylvania, USA

[5]Division of Pulmonary, Critical Care, and Sleep Medicine, University of Washington, Seattle, United States

[6]Centre for Tropical Medicine and Global Health, Nuffield Department of Medicine, University of Oxford, Churchill Hospital, Oxford, United Kingdom

[7]Department of Tropical Hygiene, Faculty of Tropical Medicine, Mahidol University, Bangkok, Thailand

For peer review only - http://bmjopen.bmj.com/site/about/guidelines.xhtml

***Corresponding author:** Assoc. Prof. Direk Limmathurotsakul, Mahidol-Oxford Tropical Medicines Research Unit, Faculty of Tropical Medicine, Mahidol University, 420/6 Rajvithi Road, Bangkok, 10400, Thailand. Tel: +66 2 203 6333, E-mail: direk@tropmedres.ac (DL)

Word count: abstract 298, text 3,914

Number of pages: 32, Tables: 4; Figures: 3, Supporting Information: 1

Keywords: sepsis, sepsis care, sepsis management, mortality, resource limited setting, natural experiment

For peer review only

For peer review only - http://bmjopen.bmj.com/site/about/guidelines.xhtml

Abstract

Objective: To evaluate the effectiveness of a Sepsis Fast Track programme (SFT) implemented at a referral hospital

Design: A natural experiment using the data of an observational study on sepsis patients (Ubon-sepsis) from March 2013 to January 2017

Setting: General medical wards and medical intensive care units (ICUs) of a referral hospital

Participants: 3,716 patients with community-acquired sepsis observed under the Ubon-sepsis cohort. Sepsis was defined as modified Sequential Organ Failure Assessment (SOFA) score ≥ 2.

Interventions: The SFT was a protocol to identify and initiate sepsis care on hospital admission and to admit patients directly to the ICUs when available. The SFT implemented at the study hospital in January 2015.

Main outcome measures: The primary outcome was 28-day mortality. The secondary outcomes were measured sepsis management.

Results: Of 3,716 patients with community-acquired sepsis, 899 were detected and enrolled in the SFT of the study hospital (SFT group) and 2,817 received standard of care (control group). Patients in the SFT group had more organ dysfunction, were more likely to receive measured sepsis management and to be admitted directly to the ICUs. 28-day mortality was 23% (205/899) in the SFT group and 20% (560/2,817) in the control group. In the primary analysis, patients in the SFT group were more likely to survive (adjusted hazard ratio for death 0.70; 95%CI 0.57-0.86, p<0.001) adjusted for admission year, gender, age, comorbidities, organ dysfunctions and direct admission to the ICUs. An interaction test showed that the effect of the SFT programme was not influenced by direct admission to the ICUs (p=0.71).

For peer review only

For peer review only - http://bmjopen.bmj.com/site/about/guidelines.xhtml

Conclusions: An implementation of SFT programme can improve sepsis care and reduce mortality of sepsis patients in rural Thailand, where some critical care resources are limited. The survival benefit is present even when patients could not be admitted directly into the ICUs.

Study registration number: NCT02217592

Strengths and limitations of this study

- The study estimated the interventional effect of an implementation of sepsis protocol in a tropical resource-limited setting by utilizing a natural experiment study design and data from a large prospective observational study.
- This study had the control group from both pre and post-intervention periods, and estimated the interventional effect by adjusting for important confounding factors which were systematically measured throughout the study period.
- The study hospital was a referral tertiary-care hospital in Thailand, and our findings may have limited generalizability to the more restricted resources settings in other LMICs.

For peer review only - http://bmjopen.bmj.com/site/about/guidelines.xhtml

INTRODUCTION

Sepsis is defined as life-threatening organ dysfunction caused by a dysregulated host response to infection,[1] and is the primary cause of death from infection, especially if not recognized and treated promptly.[2-4] Sepsis is a major cause of health loss worldwide and is associated with approximately eleven million deaths each year, most of which occur in low and middle-income countries (LMICs).[5] The United Nations World Health Assembly has recognized sepsis as a global health priority and adopted a resolution on improving its worldwide prevention, diagnosis and management.[6] Comprehensive guidelines such as those developed by the Surviving Sepsis Campaign have been associated with reduced mortality in high-income countries,[2-4] but effectiveness of these guidelines in LMICs needs more evaluation.[7-10]

Following the Surviving Sepsis Campaign (SSC) 2012,[11] the Ministry of Public Health Thailand and the Thai Society of Critical Care Medicine developed local recommendations on sepsis based on resource availability and local context.[12] The recommendations suggest that secondary-care and tertiary-care hospitals in the country should develop a Sepsis Fast Track (SFT) so that, on presentation, sepsis patients can be identified, treated and directly admitted to the ICUs when available. One small retrospective study showed lower mortality among sepsis patients enrolled than those not enrolled in the SFT (21% vs. 43%) at the study hospital,[13] while the other study did not find an association between SFT and mortality outcome.[14] Those studies are subject to selection biases due to their retrospective nature.[13 14] Interventional studies to randomize patients to receive or not receive the SFT, however, would be unethical and impractical after the national recommendations.

For peer review only - http://bmjopen.bmj.com/site/about/guidelines.xhtml

It is increasingly recommended to evaluate the impact of healthcare interventions using routine data, particularly when a wide range of routinely collected data is available.[15] A few methods can be used to perform an impact evaluation; including natural experiments (for natural or unplanned interventions), or quasi experiments (for planned or intentional interventions). Natural experiment studies have certain advantages when it is impossible to manipulate exposure to the intervention.[16] Nonetheless, a natural experiment study requires a good understanding of the process determining exposure to the intervention, a careful choice and combination of analytical methods, and transparent reporting.[16]

Here, we developed a natural experiment study by using data from our prospective observational study of community-acquired sepsis patients presenting to a referral hospital in Thailand over four years (from March 2013 to January 2017)[17,18] to evaluate the effectiveness of a SFT programme which was implemented at the study hospital in January 2015. The study is defined as a natural experiment because the detection and enrollment of patients in to the SFT programme and admission to the ICUs were neither manipulated nor influenced by the research team of the observational study.[17,18] The design, analysis and reporting of this study follow the guideline on natural experiments recently published.[16]

For peer review only - http://bmjopen.bmj.com/site/about/guidelines.xhtml

MATERIAL AND METHODS

Trial design

In this natural experiment, we evaluated the effectiveness of the SFT programme by using the data of a prospective observational study (Ubon-sepsis).[17 18] The SFT programme was implemented at the study hospital in January 2015. The SFT programme at the study hospital included (1) diagnostic criteria for attending physicians and medical teams to systematically identify sepsis patients on hospital admission (Supplementary Table 1), (2) a recommended sepsis care protocol and (3) direct admission to the ICUs when available. The SFT programme at the study hospital was generated by the SFT committee of the study hospital (S.B., S.S., C.B., P.P., B.S., O.W., P.C. and P.T.) based on SSC 2012,[11] resource availability and local context.[12]

Details of the Ubon-sepsis cohort have been published elsewhere.[17 18] In short, the Ubon-sepsis research team, who were not attending physicians or medical teams at the study hospital, conducted a prospective observational study of community-acquired infections and sepsis from March 2013 to January 2017.[17 18] The research team prospectively enrolled adult patients ≥ 18 years old who were admitted to the general medical wards and medical intensive care units (ICUs) with a primary diagnosis of infection made by the attending physician, were within 24 hours of admission to the study hospital, and had three of 20 systemic manifestations of infection documented in the medical records (Supplementary Table 2). The 20 systemic manifestations of the infections were consolidated from the 22 variables proposed as diagnostic criteria for sepsis for SSC 2012.[11] The study team sequentially screened all medical patients by reviewing admission logs in the emergency department (ED), medical wards, and medical ICUs twice daily (morning

For peer review only - http://bmjopen.bmj.com/site/about/guidelines.xhtml

and afternoon) on each working day. The Ubon-sepsis cohort was initiated in 2012 prior to the implementation of SFT at the study hospital. The research team were not involved in any clinical interventions; enrollment in the SFT programme and all medical treatment was performed by attending physicians and medical teams. The research team did not adjust the study protocol, inclusion criteria and exclusion criteria of the Ubon-sepsis cohort during the entire study period, and the research team recorded whether participants in the Ubon-sepsis cohort were enrolled in the SFT programme.

This study was defined as a natural experiment study using the definitions that (1) the intervention (enrollment in the SFT programme) was not undertaken for the purposes of research (Ubon-sepsis cohort), and (2) the variation in exposure (decision to enroll in the SFT programme) and outcomes were analysed using methods that attempt to make causal inferences.[16]

The reporting of this study follows the CONSORT guidelines and the guideline on natural experiments recently published.[16] Written, informed permission was obtained from participants prior to enrollment in the Ubon-sepsis cohort.

Participants

For this study, we evaluated patients who were included into the Ubon-sepsis cohort and had community-acquired sepsis. Sepsis was defined as an infection with organ dysfunction in accordance with the 2016 international Consensus (Sepsis-3) guidelines for sepsis.[1] Organ dysfunction was determined by a modified sequential (sepsis-based) organ failure assessment

For peer review only

For peer review only - http://bmjopen.bmj.com/site/about/guidelines.xhtml

(SOFA) score ≥2 as previously described.[17 18] The Ubon-sepsis cohort excluded patients who were suspected of having hospital-acquired infections (determined by the attending physician), hospitalized within 30 days prior to the current admission, or hospitalized in other hospital longer than 72 hours prior to study hospital admission.

Study group assignment and blinding

All patients included in the Ubon-sepsis cohort from March 2013 to December 2014 were designated as the control group. Patients included in the Ubon-sepsis cohort from January 2015 to January 2017 who received standard of care or were assigned to the SFT programme by attending medical teams using their criteria on admission (Supplementary Table 1) were designated as additional controls or as the SFT group, respectively. The Ubon-sepsis research team were not involved in decision-making regarding enrollment to the SFT programme. Due to the nature of the intervention, there was no blinding. All attending physicians and medical teams in the ED, OPD and admission awards were informed whether patients were enrolled in SFT programme.

Interventions

Patients in the control group received standard care according to local guidelines. Patients in the SFT group received the standard of care along with a recommended sepsis care protocol of the SFT programme. First, preprinted recommended doctor orders for the SFT programme were used as of January 2015 (Supplementary Figure 1). The recommended orders included oxygen administration, intravenous fluid loading and fluid administration to achieve the recommended target of 30 mL/kg crystalloid, blood culture, recommended stat (immediate) doses and choices of

For peer review only - http://bmjopen.bmj.com/site/about/guidelines.xhtml

parenteral antibiotics including ceftriaxone, ceftazidime, cloxacillin, metronidazole and gentamycin, contact ICU for ICU admission (if available), oxygen supplementation, close monitoring of vital signs and urine output, and a set of diagnostic tests including chest radiography, electrocardiogram, rapid blood glucose test, serum lactate, complete blood count, blood urea nitrogen, creatinine, electrolytes, liver function tests, albumin level, prothrombin time and partial thromboplastin time. Second, as of March 2016, the resuscitation workflow to normalize and maintain a mean arterial pressure (MAP) ≥65 mmHg, systolic blood pressure (SBP) ≥90 mmHg and urine output ≥0.5 mL/kg/hr within the first six hours was formally implemented and recommended (Supplementary Figure 2). The resuscitation workflow included fluid resuscitation, measurement of central venous pressure (CVP) and central venous oxygen saturation ($SCVO_2$), administration of adrenergic agents, blood transfusion for haematocrit <30% and hydrocortisone if adequate fluid resuscitation and vasopressor therapy could not restore hemodynamic stability. The resuscitation workflow was pre-printed and included in the clinical chart of every SFT patient (together with pre-printed doctor's order), and was recommended even if patients could not be admitted directly to the ICU. A separate set of documents, recommended management and recommended frequency of vital signs monitoring for nurses (i.e. nurse notes for SFT patients) were also used for every SFT patient. Preparation and regular meetings to implement and monitor SFT programme were organized by the SFT committee of Sunpasitthiprasong Hospital.

Outcome measures

The primary outcome measure was 28-day mortality as recorded in the Ubon-sepsis cohort.[17] 28-day mortality data were collected via telephone contact if subjects were no longer hospitalized and

For peer review only - http://bmjopen.bmj.com/site/about/guidelines.xhtml

had been discharged alive.[17] The secondary outcome measures were sepsis management interventions; including antibiotics administration, blood cultures, mechanical ventilation, adrenergic agents, acute haemodialysis and placement of a urinary catheter within the first day of hospitalization.[17 18]

Sample size

The sample size of the study was determined by the sample size of Ubon-sepsis cohort. In this natural experiment study, we estimated that about 50% of 3,716 sepsis patients in the Ubon-sepsis cohort were enrolled after the implementation of SFT programme, of which 50% were enrolled in the SFT programme (i.e. 794 and 2,382 patients were estimated to be the intervention and control group, respectively). We assumed that the mortality of the control group was 21% as previously published.[17 18] With a risk of type I error of 5% and a type II error of 20%, we calculated that our data set (n=3,716) would provide adequate power to detect the mortality difference if the mortality ratio in the SFT group compared with the control group was 0.78.

Statistical analysis

All sepsis patients were included in the analysis regardless of whether they were enrolled before or after the implementation of the SFT programme. We used the Chi-square test and Mann-Whitney test to compare the proportions of binary variables and median of continuous variables between groups, respectively. The interquartile range is presented as 25th and 75th percentiles.

For peer review only - http://bmjopen.bmj.com/site/about/guidelines.xhtml

In the primary analysis, we used multivariable Cox proportional hazard models to evaluate the effectiveness of SFT programme on 28-day mortality. The multivariable Cox proportional hazard model was used to adjust the difference between those receiving the intervention and the others for the natural experiment.[16] To reduce bias in the model development, we used the previous multivariable Cox proportional hazard model as the base model,[17] added the intervention variable and direct admission to the ICU, and modified by adding a time variable to represent possible changes over time and by using continuous modified SOFA score on admission rather than as a binary variable (modified SOFA score $\geq$2). Due to a very small number of patients enrolled in early 2017 (n=28), we considered them as enrolled in 2016. The continuous modified SOFA score was used to improve regression adjustment for disease severity of the model. The other variables included in the model were gender, age group, transfer from other hospital, comorbidities (diabetes mellitus, chronic kidney disease, liver disease and malignancy) and blood culture positive for pathogenic organisms. We tested potential predefined interactions between intervention and direct admission to the ICUs. The goodness of fit for the multivariable Cox proportional hazard model was tested with a Hosmer and Lemeshow test. Number needed to treat (NNT) was defined as the number of sepsis patients needed to be included in the SFT to avert one 28-day mortality. The NNT was estimated by using estimated hazard ratio from Cox proportional hazard model as described previously.[19]

For the secondary endpoints, we used multivariable logistic regression models with similar independent variables as the model for 28-mortality outcome and used each sepsis management process as an outcome. The multivariable logistic regression models were used to adjust for

For peer review only

For peer review only - http://bmjopen.bmj.com/site/about/guidelines.xhtml

difference in characteristics and disease severity of the patients in each group. We preformed sensitivity analyses by excluding patients enrolled prior to the implementation of the SFT programme. We also performed another sensitivity analysis by replacing direct admission to the ICUs with admission to the ICUs within the first hospital day. All analyses were performed with STATA 15.1 (StataCorp, College Station, TX, USA).

Patient and public involvement

No patients were involved in setting the research question or the outcome measures, nor were they involved in developing plans for recruitment, design, or implementation of the study. No patients were asked to advice on interpretation or writing the results. The trial results will be disseminated to the public through online social media.

RESULTS

Baseline characteristics

The observational cohort study (Ubon-sepsis) included 5,001 patients presenting with community-acquired infections from March 2013 to January 2017. 3,716 (74%) met criteria for sepsis within the first 24 hours of admission with a modified SOFA score ≥2, and were included for this natural experiment study. Figure 1 shows the flow of participants through the study. Among 3,716 sepsis patients, 899 were enrolled in the SFT programme and defined as the SFT group, and 2,817 were not enrolled in the SFT programme, received standard of care, and defined as the control group. Of 2,817 sepsis patients in the control group, 1,599 were included in the observational cohort study

For peer review only - http://bmjopen.bmj.com/site/about/guidelines.xhtml

prior to the implementation of SFT programme and 1,218 were after the implementation of the programme.

Table 1 shows the characteristic of the study patients. Patients in the SFT group were older and more likely to have underlying diseases of diabetes mellitus, cerebrovascular diseases and dyslipidemia. Patients included in the SFT group had higher severity of organ dysfunction determined by the modified SOFA score compared with the control group (median 6 [IQR 4-8] vs. 4 [IQR 3-6], p<0.001). A higher proportion of patients in the SFT group were admitted directly to the ICU compared with the control group (19% vs 5%, p<0.001).

Table 1| Baseline characteristics of sepsis patients enrolled in the Sepsis Fast Track programme[1] (SFT group) or standard of care (control group). Values are number (percentages) unless stated otherwise

**Characteristics**	**SFT group[2] (n=899)**	**Control group[3] (n=2817)**	**P value**
**Male gender**	523 (58%)	1616 (57%)	0.70
**Age (years) (median [IQR])**	63 (49-74)	56 (39-70)	<0.001
**Age group (years)**			
18-40	98 (11%)	628 (22%)	<0.001
>40-60	277 (31%)	858 (30%)	
>60-70	214 (24%)	501 (18%)	
>70	310 (34%)	830 (29%)	
**Comorbidities**			
Hypertension	239 (27%)	696 (25%)	0.26
Diabetes mellitus	213 (24%)	575 (20%)	0.04
Chronic kidney disease	129 (14%)	386 (14%)	0.63
Dyslipidemia	66 (7%)	145 (5%)	0.01
Heart disease	47 (5%)	177 (6%)	0.25

For peer review only

For peer review only - http://bmjopen.bmj.com/site/about/guidelines.xhtml

Characteristics	SFT group[2] (n=899)	Control group[3] (n=2817)	P value
Lung disease	65 (7%)	227 (8%)	0.42
Liver disease	33 (4%)	91 (3%)	0.52
Cerebrovascular disease	29 (3%)	54 (2%)	0.02
Malignancy	13 (1%)	44 (2%)	0.81
Human immunodeficiency virus (HIV)	6 (1%)	31 (1%)	0.26
**Organ dysfunction**			
Modified SOFA score (median [IQR])	6 (4-8)	4 (3-6)	<0.001
Renal dysfunction[4]	705 (78%)	1814 (64%)	<0.001
Cardiovascular dysfunction[4]	800 (89%)	1374 (49%)	<0.001
Coagulation dysfunction[4]	391 (43%)	1467 (52%)	<0.001
Liver dysfunction[4]	311 (35%)	818 (29%)	0.002
Respiratory dysfunction[4]	287 (32%)	600 (21%)	<0.001
Central nervous system dysfunction[4]	166 (18%)	523 (19%)	0.96
**Transferred from other hospitals**	**874 (97%)**	**2372 (84%)**	**<0.001**
**Duration of symptoms (median [IQR])**	**2 (1-3)**	**3 (1-5)**	**<0.001**
≤ 2 days	503 (56%)	1157 (41%)	
3-7 days	361 (40%)	1445 (51%)	
> 7 days	35 (4%)	215 (8%)	
**Presenting clinical syndromes[5]**			
Septic shock	686 (76%)	730 (26%)	<0.001
Acute febrile illness	205 (23%)	918 (33%)	<0.001
Lower respiratory infection	221 (25%)	839 (30%)	0.003
Sepsis	223 (25%)	265 (9%)	<0.001
Others	13 (1%)	446 (16%)	<0.001
Diarrheal illness	150 (17%)	261 (9%)	<0.001
**Admitted directly to an ICU upon admission**	169 (19%)	128 (5%)	<0.001
**Admitted to an ICU within 24 hours of admission**	267 (30%)	360 (13%)	<0.001
**Blood culture positive for pathogenic organisms**	176 (20%)	435 (15%)	0.002
**Year**			
2013	N/A	790 (28%)	<0.001
2014	N/A	809 (29%)	
2015	355 (39%)	634 (23%)	
2016	532 (59%)	568 (20%)	
2017	12 (1%)	16 (1%)	

275

For peer review only - http://bmjopen.bmj.com/site/about/guidelines.xhtml

[1] SFT programme was implemented at the study hospital in January 2015. This natural experiment evaluated patients in the Ubon-sepsis cohort from March 2013 to January 2017.

[2] 899 patients of the Ubon-sepsis cohort were enrolled in SFT programme after the implementation of the SFT programme (Figure 1)

[3] Included 1,599 and 1,218 patients in the Ubon-sepsis cohort before and after the implementation of the SFT programme, respectively.

[4] Organ dysfunction defined as modified SOFA score was ≥1 for each organ system.[17]

[5] Patients may have more than one presenting clinical syndrome.

Primary outcomes

The primary outcome, mortality within 28 days, occurred in 205 of 899 (23%) in the SFT group and 560 of 2,871 (20%) in the control group. In an unadjusted comparison, there was a borderline evidence showing that 28-day mortality of the SFT group was higher than control group (23% vs 20%, p=0.06). In the primary analysis, the risk of mortality was 30% lower in the SFT group compared to the control group (adjusted hazard ratio [aHR] 0.70, 95% CI 0.57-0.86, p<0.001; Table 2) adjusted for baseline characteristics, severity of sepsis and direct admission the ICUs. Older age, high modified SOFA score, underlying disease of malignancy, blood culture positive for pathogenic organisms and direct admission to the ICUs were associated with mortality outcome.

For peer review only

For peer review only - http://bmjopen.bmj.com/site/about/guidelines.xhtml

Table 2| Factors associated with 28-day mortality using multivariable Cox proportional hazards model

Variables	Died (n=765)	Survived (n=2951)	Adjusted hazard ratio (95%CI)	P value
**SFT group**[1]	205 (27%)	694 (24%)	0.70 (0.57- 0.86)	<0.001
**Male gender**	473 (58%)	2186 (52%)	0.91 (0.78- 1.06)	0.21
**Age group (years) (n [%])**				
• 18-40	68 (8%)	1072 (26%)	1.0	<0.001
• >40-60	235 (29%)	1308 (31%)	1.72 (1.28- 2.30)	
• >60-70	164 (20%)	745 (18%)	2.12 (1.56- 2.88)	
• >70	352 (43%)	1045 (25%)	3.30 (2.48- 4.39)	
**Transferred from other hospital**	749 (91%)	3067 (74%)	1.23 (1.20- 1.25)	0.07
**Modified SOFA score (median, IQR)**	7 (4-9)	4 (3-6)	1.23 (1.20- 1.25)	<0.001
**Comorbidities**				
• Diabetes mellitus	213 (26%)	793 (19%)	1.08 (0.91- 1.28)	0.37
• Chronic kidney disease	142 (17%)	403 (10%)	1.22 (1.01- 1.48)	0.04
• Liver disease	39 (5%)	94 (2%)	1.31 (0.94- 1.82)	0.11
• Malignancy	25 (3%)	57 (1%)	2.72 (1.78- 4.16)	<0.001
**Blood culture positive for pathogenic organisms**	221 (29%)	390 (13%)	1.90 (1.62- 2.22)	<0.001
**Year**				
• 2013	165 (22%)	625 (21%)	1	0.35
• 2014	177 (23%)	632 (21%)	1.00 (0.81- 1.24)	
• 2015	203 (27%)	786 (27%)	1.03 (0.82- 1.29)	
• 2016[2]	220 (29%)	908 (31%)	0.87 (0.69- 1.09)	
**Direct admission to the ICU**	128 (17%)	169 (6%)	1.70 (1.38- 2.09)	<0.001

[1] Enrolled in the Sepsis Fast Track (SFT) programme

[2] Included 28 patients in 2017

For peer review only

For peer review only - http://bmjopen.bmj.com/site/about/guidelines.xhtml

In a pre-specified interaction test, we found consistency of the intervention when stratifying by direct admission to the ICUs (interaction test p=0.71). In the multivariable model including an interaction variable, the intervention effect was comparable among those admitted directly to the ICUs (aHR 0.74, 95% CI 0.51 to 1.08) and those admitted directly to the general medical wards (aHR 0.69, 95% CI 0.57 to 0.86).

We estimated NNT by assuming that the adjusted HR 0.70 (95% CI 0.57 to 0.66) was the size of effect caused by the SFT programme. The NNT to prevent one case of death involving community-acquired sepsis was 8.1 (95% CI 5.0 to 19.8).

Secondary outcomes

Using multivariable logistic regression models, we found that patients in the SFT group were more likely to receive most sepsis management interventions than patients in the control group adjusting for baseline characteristics and severity of sepsis (Table 3). Those included antibiotics, blood cultures, adrenergic agents, and placement of a urinary catheter within the first day of hospitalization. However, sepsis patients in the SFT group were less likely to receive mechanical ventilation compared with those in the control group (adjusted odds ratio [aOR] 0.32; 95% CI 0.25 to 0.40) adjusted for baseline characteristics, severity of sepsis and direct admission to the ICUs. We found that direct admission to the ICUs (aOR 6.93, 95% CI 5.01 to 9.58) and transfer from other hospitals (aOR 4.49, 95% CI 3.08 to 6.55) were strongly associated with receiving mechanical ventilation.

For peer review only

For peer review only - http://bmjopen.bmj.com/site/about/guidelines.xhtml

Table 3| Clinical management within the first day of hospital

Clinical management	SFT group (n=899)	Control group (n=2817)	Adjusted odds ratio (95% CI)	P value
**Antibiotic**	870 (97%)	2356 (84%)	14.12 (6.11 to 32.68)	<0.001
**Blood culture**	826 (92%)	2310 (82%)	1.79 (1.32 to 2.42)	<0.001
**Urinary catherization**	858 (95%)	1599 (57%)	10.79 (7.51 to 15.50)	<0.001
**Acute dialysis**	10 (1%)	23 (0.8%)	1.90 (0.63 to 5.75)	0.26
**Adrenergic agent**	706 (79%)	900 (32%)	10.70 (8.42 to 13.60)	<0.001
**Mechanical ventilation**	305 (34%)	845 (30%)	0.32 (0.25 to 0.40)	<0.001

Sensitivity Analyses

We performed a sensitivity analysis by excluding the 1,599 patients enrolled in the observational study prior to the implementation of SFT programme. Similar differences in baseline characteristics were observed when comparing 899 patients in the SFT group to the 1,218 patients in the control group enrolled after the implementation of the SFT programme (Supplementary Table 3). A lower risk of mortality in the SFT group compared to the control group was also observed (aHR 0.65, 95% CI 0.53 to 0.81, p<0.001; Supplementary Table 4). There was no interaction between the intervention and direct admission to the ICUs (p=0.71).

We also performed another sensitivity analysis by replacing direct admission to the ICUs with admission to the ICUs within the first hospital day. Of 3,716 patients, 627 (17%) were admitted to the ICUs within the first day of admission. A lower risk of mortality in the SFT group compared to the control group was also observed (aHR 0.67, 95% CI 0.53 to 0.86, p<0.001). There was also no interaction between the intervention and admission to the ICUs within the first day of admission (p=0.56).

For peer review only

For peer review only - http://bmjopen.bmj.com/site/about/guidelines.xhtml

DISCUSSION

In this natural experiment evaluating 3,716 patients with community acquired sepsis, enrollment into a programme to identify and initiate sepsis care implemented at the study hospital (SFT programme) was associated with 30% lower risk of mortality (aHR 0.70, 95% CI 0.57 to 0.86). In recent years, there has been an increasing need to understand benefit and cost effectiveness of implementation of sepsis care interventions in LMICs because of concerns that international sepsis guidelines[11] may not be extrapolated to patients with tropical infectious diseases[7-9] and to resource-limited settings with poor ICU capacity.[10] In this trial we show effectiveness of implementation of sepsis protocol modified based on resource availability in a tropical country, where causes of community-acquired sepsis include malaria and tropical viral diseases.[17 20 21] Access to the ICU increased after the implementation of the SFT programme, but the majority of sepsis patients were still managed on the general wards, including those with respiratory failure or shock. Nonetheless, our study shows that enhancing sepsis care in the emergency department and general medical wards, as well as improving access to ICUs can reduce sepsis mortality in a LMIC.

The negative association between the intervention and received mechanical ventilation could be a sign of improved sepsis care. Patients in the SFT group are monitored closely either in or outsides the ICUs, and the attending physicians aim to obviate the need for airway intubation when possible.[7] Attending physicians may tend to provide mechanical ventilation to patients in the control group based on broad indications such as (1) airway protection, (2) hypercapnic respiratory failure, (3) hypoxemic respiratory failure or (4) circulatory failure[22 23] because they may not be

For peer review only - http://bmjopen.bmj.com/site/about/guidelines.xhtml

able to monitor patients' breathing and oxygen saturation as often as those enrolled in the SFT programme.

Patients in the SFT group had more organ dysfunction than those in the control group because the SFT programme enrolled patients on admission and, therefore, could not use laboratory test results from blood samples drawn on admission. However, the control group were defined as having sepsis based on clinical findings and all laboratory test results within 24 hours of admission (per protocol of Ubon-sepsis cohort study[17 18]). Therefore, the control group could use laboratory test results (i.e. liver function tests, creatinine level, international normalised ratio and activated partial thromplastin time) from blood specimens drawn on admission. Therefore, the SFT programme were more likely to enroll patients with obvious signs of sepsis and septic shock; such as acute respiratory failure and hypotension, while Ubon-sepsis cohort could include sepsis patients with relatively lower modified SOFA score.

Comparison with other studies

Our study is not the first to evaluate effectiveness of sepsis intervention in LMICs. Early recognition and protocol directed intervention improves outcomes of sepsis in adults[24] and severe infection in children[25] in LMICs. The optimal method of fluid resuscitation in sepsis in tropical LMICs has not been determined.[8 24 26 27] Our resuscitation protocol is a simple guideline, and the SFT recommend doctors to be careful and adjust fluid resuscitation based on preliminary diagnoses, underlying diseases and rapid diagnostic test results (i.e. if sepsis is caused by malaria or dengue infection). The implementation of the SFT programme in our study hospital and in

For peer review only - http://bmjopen.bmj.com/site/about/guidelines.xhtml

Thailand is consistent with the recommendation of "SCAN-TEACH-TREAT" programme developed by Sepsis in Resource-Limited Settings Workgroup of the Surviving Sepsis Campaign.[7] The SFT programme evaluated resources in the setting (SCAN component), focused on educational interventions on early recognition and management of sepsis among medical personnel including physicians, nurses and students (TEACH component) and implemented pragmatic and simple bundles into practice (TREAT component). In addition, the SFT programme has the strong support and endorsement of local health and governmental leaders.[12]

For peer review only

Strength and limitations of the study

This study features three strengths. First, it took advantage of a robust prospective observational study design that strengthened causal inference by providing pre-intervention information, having an appropriate control group from both pre and post-intervention periods, and controlling important confounding factors (i.e. the modified SOFA score) which were measured systematically throughout the study period. Second, this study incorporated several predictors of interest (measured sepsis management interventions and admission to the ICUs). This allows us to identify that most measured sepsis interventions increased and that admission to the ICUs were not associated with the interventional effect. Third, the focus on sepsis at a public tertiary-care hospital in Thailand helped us to estimate the interventional effect of an implementation of sepsis protocol in a tropical resource-limited setting.

Our study had several limitations. First, our findings may not be able to generalize to all hospitals and all sepsis protocols in LMICs. It is recommended that each resource-limited setting should set

For peer review only - http://bmjopen.bmj.com/site/about/guidelines.xhtml

up their own SCAN-TEACH-TREAT programme, and closely monitor and evaluate the effectiveness of an intervention implemented.[7] Second, a modified SOFA score was used because the dosage of dobutamine, dopamine, epinephrine and norepinephrine were not recorded and arterial blood gases were rarely performed. The modified SOFA score (maximum 23) may be lower than the SOFA score (maximum 24). Nonetheless, the modified SOFA score is strongly associated with mortality in sepsis.[17 18] Third, the cost of implementing the SFT programme was not formally estimated.

Conclusions and future implications

Our study successfully measured effectiveness of a sepsis programme implemented in a LMIC. Measuring effectiveness of a sepsis programme is a complex issue, and we utilized a natural experiment and carefully controlled for severity of sepsis and temporal trends in our analyses. Care in sepsis patients improved after the implementation of the programme. While the natural experiment adds to our knowledge of effectiveness of a sepsis programme, additional research is needed to better understand cost of the intervention, long-term benefits and impact of the programme on a national scale. National policies aimed at saving lives from sepsis in LMICs should not hesitate to analyze their resources and situations, and then develop, implement and monitor their programmes.

For peer review only

For peer review only - http://bmjopen.bmj.com/site/about/guidelines.xhtml

Acknowledgement:

We thank all patients, their relatives, and staff of the Sunpasitthiprasong hospital who participated in the study. We thank Mayura Malasit, Praweennuch Watanachaiprasert, Chayamon Krainoonsing, Passaraporn Kesaphun, Nannicha Jirapornuwat, Gumphol Wongsuvan, Areeya Faosap, Yaowaret Dokket, Sukhumal Pewlaorng, Jintana Suwannapruek, Prapass Wannapinij and Diane Tomita for their clinical, laboratory and administrative support.

Contributors:

NPJD, TEW, and DL obtained grant funding. SB, VH, PT, TEW, and DL contributed to study conception development and study design. SB, VH, PT, TEW and DL contributed to study conduct, data collection, and study administration. VH and DL performed the statistical analysis and interpreted the data and had full access to all of the data in the study. Both authors can take responsibility for the integrity of the data and the accuracy of the data analysis. DL is a guarantor. SB, VH, PT, TEW, and DL wrote the first draft of a manuscript. All authors contributed to results interpretation, and critically revised and approved the final submitted manuscript. The corresponding author attests that all listed authors meet authorship criteria and that no others meeting the criteria have been omitted.

Funding:

The study was funded by the Wellcome Trust (090219/Z/09/Z) and National Heart, Lung and Blood Institute, National Institutes of Health (R01HL113382). DL is supported by an intermediate fellowship from the Wellcome Trust (101103/Z/13/Z). The funders had no role in the design and

For peer review only

For peer review only - http://bmjopen.bmj.com/site/about/guidelines.xhtml

447 conduct of the study, all study procedures, data collection, data analyses, data interpretation,
448 writing of the report, and the decision to submit the article for publication.
449
450 **Competing interests:**
451 The authors declare that they have no completing interests.
452
453 **Ethics approvals:**
454 The study was conducted the study in full compliance with the principles of good clinical practice
455 (GCP), and the ethical principles of the Declaration of Helsinki. The study protocol and related
456 documents were approved by Sunpasitthiprasong Hospital Ethics Committee (039/2556), the
457 Ethics Committee of the Faculty of Tropical Medicine, Mahidol University (MUTM2012-024-
458 01), the University of Washington Institutional Review Board (42988) and the Oxford Tropical
459 Research Ethics Committee at the University of Oxford (OXTREC172-12). Signed or
460 fingerprinted informed consent was obtained from the participants or their representatives before
461 enrollment.
462
463 **Data sharing:**
464 The final database with the data dictionary are publicly available online
465 https://doi.org/10.6084/m9.figshare.12102627.
466 The lead authors (SB, VH and DL) affirm that the manuscript is an honest, accurate, and
467 transparent account of the study being reported; that no important aspects of the study have been
468 omitted; and that any discrepancies from the study as planned have been explained. This is an

For peer review only - http://bmjopen.bmj.com/site/about/guidelines.xhtml

Open Access article distributed in accordance with the terms of the Creative Commons Attribution (CC BY 4.0) license, which permits unrestricted reuse, distribution, and reproduction in any medium, provided the original work is properly cited. See: http://creativecommons.org/licenses/by/4.0/

Patient and Public Involvement

No patients were involved in setting the research question or the outcome measures, and interpretation or writing up of results. The results of this study will be disseminated to physicians at the study hospital, health care providers, policy makers, and academic communities through various mediums, including printed report, internal hospital meetings, academic conferences, and institutional networks. The results from this study will be used to inform the current Sepsis Fast Track programme at Sunpasitthiprasong hospital and the community hospitals which are located in jurisdiction of the study hospital catchment areas in the Northeast Thailand. The study results will not be disseminated to patients or general population, because study results are in medical context.

For peer review only - http://bmjopen.bmj.com/site/about/guidelines.xhtml

References

1 Singer M, Deutschman CS, Seymour CW, et al. The Third International Consensus Definitions for Sepsis and Septic Shock (Sepsis-3). *JAMA* 2016;315(8):801-10. doi: 10.1001/jama.2016.0287

2 Rhodes A, Evans LE, Alhazzani W, et al. Surviving Sepsis Campaign: International Guidelines for Management of Sepsis and Septic Shock 2016. *Crit Care Med* 2017;45(3):486-552. doi: 10.1097/ccm.0000000000002255

3 WHO. WHO Sepsis Technical Expert Meeting - Meeting report Geneva: World Health Organization; 2018 [Available from: https://www.who.int/servicedeliverysafety/areas/sepsis_meeting-report-2018.pdf accessed 25 November 2019 2019].

4 Vincent J-L. The Clinical Challenge of Sepsis Identification and Monitoring. *PLoS Med* 2016;13(5):e1002022. doi: 10.1371/journal.pmed.1002022

5 Rudd KE, Johnson SC, Agesa KM, et al. Global, regional, and national sepsis incidence and mortality, 1990–2017: analysis for the Global Burden of Disease Study. *The Lancet* 2020;395(10219):200-11. doi: 10.1016/S0140-6736(19)32989-7

6 Reinhart K, Daniels R, Kissoon N, et al. Recognizing Sepsis as a Global Health Priority — A WHO Resolution. *N Engl J Med* 2017;377(5):414-17. doi: 10.1056/NEJMp1707170

For peer review only

For peer review only - http://bmjopen.bmj.com/site/about/guidelines.xhtml

7 Kwizera A, Baelani I, Mer M, et al. The long sepsis journey in low- and middle-income countries begins with a first step...but on which road? *Crit Care* 2018;22(1):64. doi: 10.1186/s13054-018-1987-z

8 McGloughlin S, Richards GA, Nor MBM, et al. Sepsis in tropical regions: Report from the task force on tropical diseases by the World Federation of Societies of Intensive and Critical Care Medicine. *J Crit Care* 2018;46:115-18. doi: 10.1016/j.jcrc.2017.12.018

9 Becker JU, Theodosis C, Jacob ST, et al. Surviving sepsis in low-income and middle-income countries: new directions for care and research. *Lancet Infect Dis* 2009;9(9):577-82. doi: 10.1016/S1473-3099(09)70135-5

10 Schultz MJ, Dunser MW, Dondorp AM, et al. Current challenges in the management of sepsis in ICUs in resource-poor settings and suggestions for the future. *Intensive Care Med* 2017;43(5):612-24. doi: 10.1007/s00134-017-4750-z

11 Dellinger RP, Levy MM, Rhodes A, et al. Surviving Sepsis Campaign: international guidelines for management of severe sepsis and septic shock, 2012. *Intensive Care Med* 2013;39(2):165-228. doi: 10.1007/s00134-012-2769-8

12 Office of permanent secretatry, Ministry of Public Health, Thailand. The Development of Health Service Plan and Indicators of Health Outcomes 2018:8.

13 Ruangchan S, Chusri S, Saengsanga P, et al. Clinical Outcomes of Community-Acquired Severe Sepsis after Implementation of a Simple Severe Sepsis Fast Track. *J Med Assoc Thai* 2016;99(8):877-85.

For peer review only

For peer review only - http://bmjopen.bmj.com/site/about/guidelines.xhtml

14 Ittisanyakorn M, Ruchichanantakul S, Vanichkulbodee A, et al. Prevalence and factors associated with one-year mortality of infectious diseases among elderly emergency department patients in a middle-income country. *BMC Infect Dis* 2019;19(1):662. doi: 10.1186/s12879-019-4301-z

15 Clarke GM, Conti S, Wolters AT, et al. Evaluating the impact of healthcare interventions using routine data. *BMJ* 2019;365:l2239. doi: 10.1136/bmj.l2239

16 Craig P, Cooper C, Gunnell D, et al. Using natural experiments to evaluate population health interventions: new Medical Research Council guidance. *J Epidemiol Community Health* 2012;66(12):1182-6. doi: 10.1136/jech-2011-200375

17 Hantrakun V, Somayaji R, Teparrukkul P, et al. Clinical epidemiology and outcomes of community acquired infection and sepsis among hospitalized patients in a resource limited setting in Northeast Thailand: A prospective observational study (Ubon-sepsis). *PLoS One* 2018;13(9):e0204509. doi: 10.1371/journal.pone.0204509

18 Rudd KE, Hantrakun V, Somayaji R, et al. Early management of sepsis in medical patients in rural Thailand: a single-center prospective observational study. *J Intensive Care* 2019;7:55. doi: 10.1186/s40560-019-0407-z

19 Altman DG, Andersen PK. Calculating the number needed to treat for trials where the outcome is time to an event. *BMJ* 1999;319(7223):1492. doi: 10.1136/bmj.319.7223.1492

20 Teparrukkul P, Hantrakun V, Imwong M, et al. Utility of qSOFA and modified SOFA in severe malaria presenting as sepsis. *PLoS One* 2019;14(10):e0223457. doi: 10.1371/journal.pone.0223457

For peer review only

For peer review only - http://bmjopen.bmj.com/site/about/guidelines.xhtml

21 Teparrukkul P, Hantrakun V, Day NPJ, et al. Management and outcomes of severe dengue patients presenting with sepsis in a tropical country. *PLoS One* 2017;12(4):e0176233. doi: 10.1371/journal.pone.0176233

22 Pierson DJ. Indications for mechanical ventilation in adults with acute respiratory failure. *Respir Care* 2002;47(3):249-62; discussion 62-5.

23 Pham T, Brochard LJ, Slutsky AS. Mechanical Ventilation: State of the Art. *Mayo Clin Proc* 2017;92(9):1382-400. doi: 10.1016/j.mayocp.2017.05.004

24 Jacob ST, Banura P, Baeten JM, et al. The impact of early monitored management on survival in hospitalized adult Ugandan patients with severe sepsis: a prospective intervention study*. *Crit Care Med* 2012;40(7):2050-8. doi: 10.1097/CCM.0b013e31824e65d7

25 Sazawal S, Black RE, Pneumonia Case Management Trials G. Effect of pneumonia case management on mortality in neonates, infants, and preschool children: a meta-analysis of community-based trials. *Lancet Infect Dis* 2003;3(9):547-56. doi: 10.1016/s1473-3099(03)00737-0

26 Maitland K, Kiguli S, Opoka RO, et al. Mortality after fluid bolus in African children with severe infection. *N Engl J Med* 2011;364(26):2483-95. doi: 10.1056/NEJMoa1101549

27 Andrews B, Semler MW, Muchemwa L, et al. Effect of an Early Resuscitation Protocol on In-hospital Mortality Among Adults With Sepsis and Hypotension: A Randomized Clinical Trial. *JAMA* 2017;318(13):1233-40. doi: 10.1001/jama.2017.10913

For peer review only

For peer review only - http://bmjopen.bmj.com/site/about/guidelines.xhtml

For peer review only

For peer review only - http://bmjopen.bmj.com/site/about/guidelines.xhtml

572 **Figure legends**

573 **Figure 1| Flow of participants through study**

574 **Footnote of figure 1:** This natural experiment used the data of an observational study on sepsis
575 patients (Ubon-sepsis) from March 2013 to January 2017 to evaluate the effectiveness of a Sepsis
576 Fast Track (SFT) programme implemented at the study hospital in January 2015

For peer review only

For peer review only - http://bmjopen.bmj.com/site/about/guidelines.xhtml

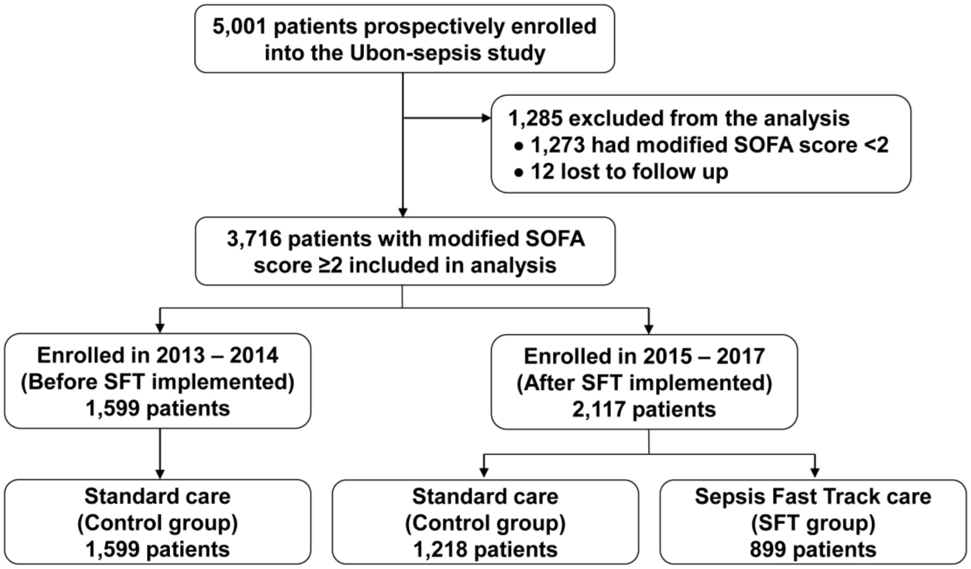
5,001 patients prospectively enrolled into the Ubon-sepsis study
1,285 excluded from the analysis
• 1,273 had modified SOFA score <2
• 12 lost to follow up
3,716 patients with modified SOFA score ≥2 included in analysis
Enrolled in 2013 – 2014 (Before SFT implemented) 1,599 patients
Enrolled in 2015 – 2017 (After SFT implemented) 2,117 patients
Standard care (Control group) 1,599 patients
Standard care (Control group) 1,218 patients
Sepsis Fast Track care (SFT group) 899 patients

Effectiveness of a sepsis programme implemented in a resource-limited setting: a natural experiment

Suchart Booraphun MD[1], Viriya Hantrakun PhD[2], Suwatthiya Siriboon MD[1], Chaiyaporn Boonsri MD[1], Pulyamon Poomthong MD[1], Bung-Orn Singkaew BNS[1], Oratai Wasombat BNS[1], Parinya Chamna MD, PhD[1], Ratapum Champunot MD[3], Kristina Rudd MD, MPH[4, 5], Nicholas PJ. Day[2,6] MD[2], PhD, Arjen Dondrop MD[2,6], PhD, Prapit Teparrukkul MD[1], T. Eoin West MD, MPH[5], Direk Limmathurotsakul MD, PhD[2,6,7]

Short Title: Sepsis Fast Track Thailand

[1]Sunpasitthiprasong Hospital, Ubon Ratchathani, Thailand

[2]Mahidol Oxford Tropical Medicine Research Unit, Faculty of Tropical Medicine, Mahidol University, Bangkok, Thailand

[3]Department of Internal Medicine, Buddhachinaraj Phitsanulok Hospital, Phitsanulok, Thailand

[4]Department of Critical Care Medicine, University of Pittsburgh, Pittsburgh, Pennsylvania, USA

[5]Division of Pulmonary, Critical Care, and Sleep Medicine, University of Washington, Seattle, United States

[6]Centre for Tropical Medicine and Global Health, Nuffield Department of Medicine, University of Oxford, Churchill Hospital, Oxford, United Kingdom

[7]Department of Tropical Hygiene, Faculty of Tropical Medicine, Mahidol University, Bangkok, Thailand

For peer review only

Supplementary Table 1 | Criteria used to systematically enroll patents into Sepsis Fast Track (SFT) upon admission

1. **Present with 2 or more of below Signs of systemic inflammatory response syndrome (SIRS)**
    - Body temperature > 38.3 °C or < 36.0 °C
    - Heart rate > 90 bpm
    - Respiratory rate > 20 pm or $PaCO_2$ < 32 mmHg
    - WBC > 12,000 /µL or < 4,000 /µL or Band forms > 10%
2. **Suspected sources of infection**
    - Pneumonia
    - Urinary track infection
    - Intra-abdominal infection
    - Skin and soft tissue infection
    - CNS infection
    - Others infections or unspecified source of infection
3. **Diagnostic criteria for severe sepsis:** patient met criteria in no 1 and 2 and has at least one of the following criteria
    - Mottled skin
    - Capillary refilling time ≥ 3 seconds
    - Urine output < 0.5 ml/kg/hour
    - Abrupt change in mental status
    - Acute respiratory failure
    - Platelet count < 100,000 /µL
    - Disseminated intravascular coagulation
    - Lactate > 2 mmol/L
    - SBP < 90 mmHg or MAP < 65 mmHg
4. **Diagnostic criteria for septic shock:** patient who are severe sepsis and has at least 1 of the following criteria
    - SBP < 90 mmHg or MAP < 65 mmHg after crystalliod administration ≥ 40-60 ml/kg of body weight OR after colloid administration ≥ 20-30 ml/kg of body weight
    - Require administration of dopamine > 5µg/kg of BW/min or norepinephrine / epinephrine > 0.02 µg/kg of BW/min to maintain MAP to be > 65 mmHg

For peer review only - http://bmjopen.bmj.com/site/about/guidelines.xhtml

Supplementary Table 2 | Systemic manifestation of infection criteria used for enrollment in Ubon-Sepsis Cohort

General parameters

1. Fever or hypothermia (Core body temperature defined as > 38.3 °C or < 36.0 °C)
2. Tachycardia (heart rate > 90 beats per minute)
3. Tachypnea (respiratory rate > 20 per minute)
4. Altered mental status with Glasgow Coma Score (GCS) < 15 or <10 if intubated
5. Hyperglycemia (plasma glucose > 140 mg/dL) in the absence of diabetes

Inflammatory parameters

6. Leukocytosis (white blood cell count > 12,000/µL), leukopenia (white blood cell count < 4000/µL) or immature forms > 10%
7. Plasma C-reactive protein > 2 SD above the normal value
8. Plasma procalcitonin > 2 SD above the normal value

Hemodynamic parameters

9. Arterial hypotension (systolic blood pressure (SBP) < 90 mmHg, mean arterial pressure (MAP) < 70 mmHg, or SBP decrease > 40 mmHg)

Organ dysfunction parameters

10. Low oxygen saturation determined by pulse oximetry (SpO_2 < 95%) determined by pulse oximetry
11. Arterial hypoxemia (PaO_2 / FIO_2 < 300)
12. Acute oliguria (urine output < 0.5 mL/kg/hr or 45 mmol/L for 2 hours)
13. Creatinine increase > 0.5 mg/dL
14. Coagulation abnormalities (international normalised ratio >1·5 or activated partial thromboplastin time >60 seconds)
15. Thrombocytopenia (Platelet count < 100,000 cells/µL)
16. Ileus (absent bowel sounds)
17. Hyperbilirubinaemia (plasma total bilirubin > 4 mg/dL)

Tissue perfusion parameters

18. Hyperlactatemia (> 1 mmol/L)
19. Decreased capillary refill or mottling
20. Significant edema or positive fluid balance

For peer review only

For peer review only - http://bmjopen.bmj.com/site/about/guidelines.xhtml

Supplementary Table 3 | Baseline characteristics of sepsis patients included in the Sepsis Fast Track (SFT) programme or standard of care (control) after the implementation of SFT programme. Values are number (percentages) unless stated otherwise

**Characteristics**	**SFT group[2] (n=899)**	**Control group[3] (n=1218)**	**P value**
**Male gender**	523 (58%)	702 (58%)	0.82
**Age (years) (median [IQR])**	63 (49-74)	59 (41-73)	<0.001
**Age group (years) (n [%])**			
18-40	98 (11%)	282 (23%)	<0.001
>40-60	277 (31%)	342 (28%)	
>60-70	214 (24%)	221 (18%)	
>70	310 (34%)	373 (31%)	
**Comorbidities**	484 (54%)	639 (52%)	0.53
Hypertension	239 (27%)	332 (27%)	0.73
Diabetes mellitus	213 (24%)	274 (23%)	0.52
Chronic kidney disease	129 (14%)	195 (16%)	0.29
Dyslipidemia	66 (7%)	76 (6%)	0.32
Heart disease	47 (5%)	96 (8%)	0.02
Lung disease	65 (7%)	116 (10%)	0.06
Liver disease	33 (4%)	46 (4%)	0.90
Cerebrovascular disease	29 (3%)	25 (2%)	0.09
Malignancy	13 (1%)	32 (3%)	0.06
Human immunodeficiency virus (HIV)	6 (1%)	9 (1%)	0.85
**Organ dysfunction[4]**			
Total modified SOFA score (median [IQR])	6 (4-8)	4 (3-6)	<0.001
Renal dysfunction[4]	705 (78%)	728 (60%)	<0.001
Cardiovascular dysfunction[4]	800 (89%)	437 (36%)	<0.001
Coagulation dysfunction[4]	391 (43%)	612 (50%)	0.002
Liver dysfunction[4]	311 (35%)	314 (26%)	<0.001
Respiratory dysfunction[4]	287 (32%)	339 (28%)	0.04
Central nervous system dysfunction[4]	166 (18%)	214 (18%)	0.61
**Transferred from other hospitals**	874 (97%)	1009 (83%)	<0.001
**Duration of symptoms (median [IQR])**	2 (1-3)	3 (1-5)	<0.001
≤ 2 days	503 (56%)	519 (43%)	<0.001
3-7 days	361 (40%)	624 (51%)	
> 7 days	35 (4%)	75 (6%)	

For peer review only

For peer review only - http://bmjopen.bmj.com/site/about/guidelines.xhtml

Characteristics	SFT group[2] (n=899)	Control group[3] (n=1218)	P value
**Presenting clinical syndromes[5] (n [%])**			
Septic shock	686 (76%)	179 (15%)	<0.001
Acute febrile illness	205 (23%)	469 (39%)	<0.001
Lower respiratory infection	221 (25%)	431 (35%)	<0.001
Sepsis	223 (25%)	131 (11%)	<0.001
Others	13 (1%)	163 (13%)	<0.001
Diarrheal illness	150 (17%)	102 (8%)	<0.001
**Admitted directly to an ICU upon admission**	169 (19%)	39 (3%)	<0.001
**Blood culture positive for pathogenic organisms**	176 (20%)	164 (13%)	<0.001
**Year**			
2015	355 (39%)	634 (52%)	<0.001
2016	532 (59%)	568 (47%)	
2017	12 (1%)	16 (1%)	

[1]Organ dysfunction is defined by modified SOFA ≥ 2

[2]Sepsis patients who were identified and treated in Sepsis-FT system in 2015 – January 2018.

[3]Sepsis patients whom were not in Sepsis-FT system; 1599/2871 patients were enrolled into the study during 2013-2014 before Sepsis-FT implemented in the hospital.

[4]Organ dysfunction defined as modified SOFA score was ≥ 1 for each organ system [13].

[5]Patients may have more than one presenting clinical syndrome.

For peer review only

For peer review only - http://bmjopen.bmj.com/site/about/guidelines.xhtml

Supplementary Table 4 | Factors associated with 28-day mortality using multivariable Cox proportional hazards model in 2,117 patients enrolled into the study after the implementation of the Sepsis Fast Track (SFT) programme

Variables	Died (n=423)	Survived (n=1694)	Adjusted hazard ratio (95%CI)	P value
**SFT group**	205(48%)	694(41%)	0.65 (0.53- 0.81)	<0.001
**Male gender**	251(59%)	974(57%)	0.91 (0.75- 1.12)	0.38
**Age group (years) (n [%])**				
• 18-40	30(7%)	350(21%)	1	<0.001
• >40-60	125(30%)	494(29%)	2.05 (1.37- 3.06)	
• >60-70	83(20%)	352(21%)	1.93 (1.26- 2.97)	
• >70	185(44%)	498(29%)	3.19 (2.14- 4.74)	
**Transferred from other hospital**	403(95%)	1480(87%)	1.82 (1.15- 2.90)	0.01
**Modified SOFA score (median, IQR)**	7 (5-10)	4 (3-6)	1.25 (1.21- 1.28)	<0.001
**Comorbidities**				
• Diabetes mellitus	120(28%)	367(22%)	1.06 (0.85- 1.33)	0.59
• Chronic kidney disease	83(20%)	241(14%)	1.21 (0.94- 1.56)	0.14
• Liver disease	22(5%)	57(3%)	1.06 (0.68- 1.64)	0.81
• Malignancy	19(4%)	26(2%)	3.10 (1.94- 4.96)	<0.001
**Blood culture positive for pathogenic organisms**	115(27%)	225(13%)	1.80 (1.44- 2.24)	<0.001
**Year**				
• 2015	203(48%)	786(46%)	1	0.10
• 2016	220(52%)	908(54%)	0.85 (0.70- 1.03)	
**Direct admission to the ICU**	85(20%)	123(7%)	1.71 (1.31- 2.23)	<0.001

For peer review only

Supplementary Figure 1 | Preprinted recommended doctor orders for sepsis fast track programme used at the Emergency Department at Sunpasitthiprasong Hopsital from 1 January 2015

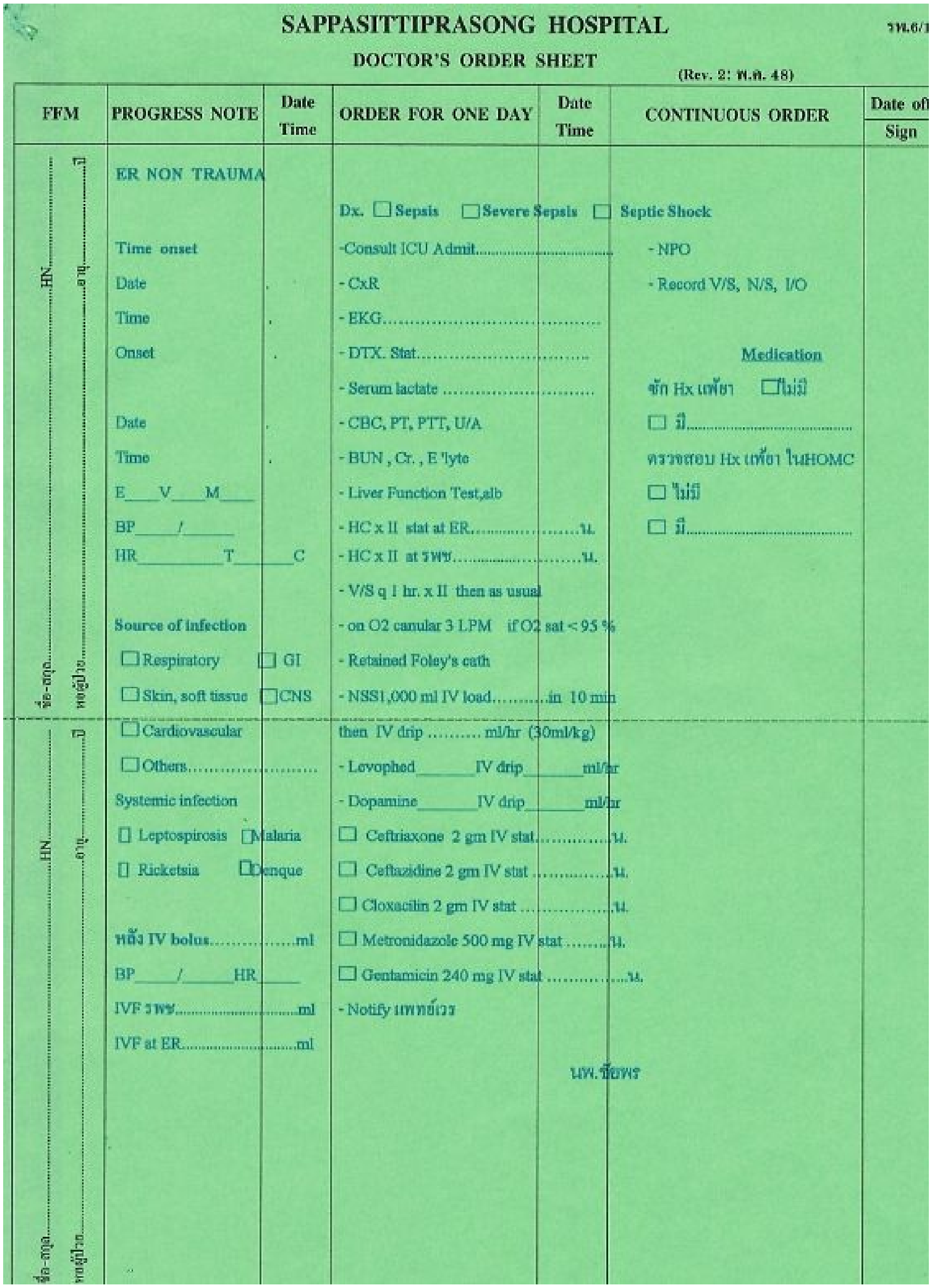
SAPPASITTIPRASONG HOSPITAL

DOCTOR'S ORDER SHEET

(Rev. 2: พ.ศ. 48)

FFM	PROGRESS NOTE	Date Time	ORDER FOR ONE DAY	Date Time	CONTINUOUS ORDER	Date of Sign
ชื่อ-สกุล HN อายุ ปี หอผู้ป่วย	ER NON TRAUMA<br>Time onset<br>Date<br>Time<br>Onset<br>Date<br>Time<br>E___V___M___<br>BP___/___<br>HR___T___C<br>Source of infection<br>☐ Respiratory ☐ GI<br>☐ Skin, soft tissue ☐ CNS<br>☐ Cardiovascular<br>☐ Others........<br>Systemic infection<br>☐ Leptospirosis ☐ Malaria<br>☐ Ricketsia ☐ Denque<br>หลัง IV bolus.........ml<br>BP___/___HR___<br>IVF รพช.........ml<br>IVF at ER.........ml		Dx. ☐ Sepsis ☐ Severe Sepsis ☐ Septic Shock<br>-Consult ICU Admit........<br>- CxR<br>- EKG........<br>- DTX. Stat........<br>- Serum lactate........<br>- CBC, PT, PTT, U/A<br>- BUN , Cr. , E 'lyte<br>- Liver Function Test,alb<br>- HC x II stat at ER........น.<br>- HC x II at รพช........น.<br>- V/S q 1 hr. x II then as usual<br>- on O2 canular 3 LPM if O2 sat < 95 %<br>- Retained Foley's cath<br>- NSS1,000 ml IV load........in 10 min<br>then IV drip .... ml/hr (30ml/kg)<br>- Levophed___IV drip___ml/hr<br>- Dopamine___IV drip___ml/hr<br>☐ Ceftriaxone 2 gm IV stat........น.<br>☐ Ceftazidine 2 gm IV stat........น.<br>☐ Cloxacilin 2 gm IV stat........น.<br>☐ Metronidazole 500 mg IV stat........น.<br>☐ Gentamicin 240 mg IV stat........น.<br>- Notify แพทย์เวร<br>นพ.ชัยพร		- NPO<br>- Record V/S, N/S, I/O<br>Medication<br>ซัก Hx แพ้ยา ☐ไม่มี<br>☐ มี........<br>ตรวจสอบ Hx แพ้ยา ในHOMC<br>☐ ไม่มี<br>☐ มี........	

109 **Supplementary Figure 2 | The Sepsis Fast Track sepsis resuscitation workflow used at the**
110 **Emergency Department at Sunpasitthiprasong Hopsital from 1 March 2016**

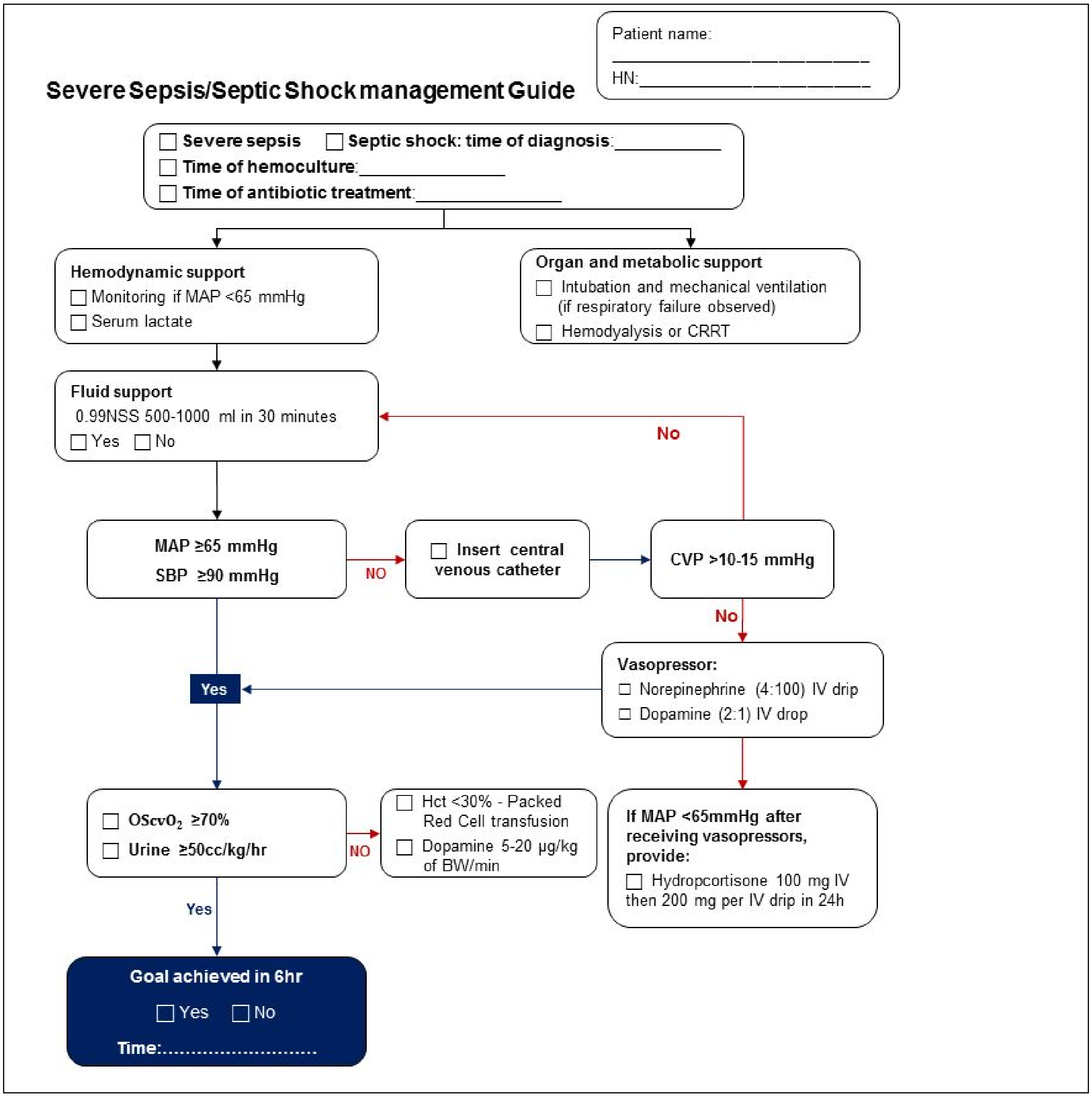

111

112

CONSORT 2010 checklist of information to include when reporting a randomised trial*

Section/Topic	Item No	Checklist item	Reported on page No
**Title and abstract**			
	1a	Identification as a randomised trial in the title	1
	1b	Structured summary of trial design, methods, results, and conclusions (for specific guidance see CONSORT for abstracts)	3
**Introduction**			
Background and objectives	2a	Scientific background and explanation of rationale	5-6
	2b	Specific objectives or hypotheses	6
**Methods**			
Trial design	3a	Description of trial design (such as parallel, factorial) including allocation ratio	7-8
	3b	Important changes to methods after trial commencement (such as eligibility criteria), with reasons	-
Participants	4a	Eligibility criteria for participants	8-9
	4b	Settings and locations where the data were collected	7
Interventions	5	The interventions for each group with sufficient details to allow replication, including how and when they were actually administered	9-10
Outcomes	6a	Completely defined pre-specified primary and secondary outcome measures, including how and when they were assessed	10-11
	6b	Any changes to trial outcomes after the trial commenced, with reasons	-
Sample size	7a	How sample size was determined	11
	7b	When applicable, explanation of any interim analyses and stopping guidelines	-
Randomisation:			
Sequence generation	8a	Method used to generate the random allocation sequence	9
	8b	Type of randomisation; details of any restriction (such as blocking and block size)	-
Allocation concealment mechanism	9	Mechanism used to implement the random allocation sequence (such as sequentially numbered containers), describing any steps taken to conceal the sequence until interventions were assigned	9
Implementation	10	Who generated the random allocation sequence, who enrolled participants, and who assigned participants to interventions	9
Blinding	11a	If done, who was blinded after assignment to interventions (for example, participants, care providers, those	-

For peer review only

For peer review only - http://bmjopen.bmj.com/site/about/guidelines.xhtml

		assessing outcomes) and how	
	11b	If relevant, description of the similarity of interventions	-
Statistical methods	12a	Statistical methods used to compare groups for primary and secondary outcomes	11-13
	12b	Methods for additional analyses, such as subgroup analyses and adjusted analyses	13
**Results**			
Participant flow (a diagram is strongly recommended)	13a	For each group, the numbers of participants who were randomly assigned, received intended treatment, and were analysed for the primary outcome	13-14
	13b	For each group, losses and exclusions after randomisation, together with reasons	-
Recruitment	14a	Dates defining the periods of recruitment and follow-up	13
	14b	Why the trial ended or was stopped	-
Baseline data	15	A table showing baseline demographic and clinical characteristics for each group	14-15
Numbers analysed	16	For each group, number of participants (denominator) included in each analysis and whether the analysis was by original assigned groups	16-17
Outcomes and estimation	17a	For each primary and secondary outcome, results for each group, and the estimated effect size and its precision (such as 95% confidence interval)	16-19
	17b	For binary outcomes, presentation of both absolute and relative effect sizes is recommended	17-19
Ancillary analyses	18	Results of any other analyses performed, including subgroup analyses and adjusted analyses, distinguishing pre-specified from exploratory	19
Harms	19	All important harms or unintended effects in each group (for specific guidance see CONSORT for harms)	-
**Discussion**			
Limitations	20	Trial limitations, addressing sources of potential bias, imprecision, and, if relevant, multiplicity of analyses	22-23
Generalisability	21	Generalisability (external validity, applicability) of the trial findings	23
Interpretation	22	Interpretation consistent with results, balancing benefits and harms, and considering other relevant evidence	20-22
**Other information**			
Registration	23	Registration number and name of trial registry	4
Protocol	24	Where the full trial protocol can be accessed, if available	-
Funding	25	Sources of funding and other support (such as supply of drugs), role of funders	24-25

*We strongly recommend reading this statement in conjunction with the CONSORT 2010 Explanation and Elaboration for important clarifications on all the items. If relevant, we also recommend reading CONSORT extensions for cluster randomised trials, non-inferiority and equivalence trials, non-pharmacological treatments, herbal interventions, and pragmatic trials. Additional extensions are forthcoming: for those and for up to date references relevant to this checklist, see www.consort-statement.org.

For peer review only - http://bmjopen.bmj.com/site/about/guidelines.xhtml
For peer review only

BMJ Open

Effectiveness of a sepsis programme in a resource-limited setting

Journal:	*BMJ Open*
Manuscript ID	bmjopen-2020-041022.R1
Article Type:	Original research
Date Submitted by the Author:	19-Oct-2020

Complete List of Authors:	Booraphun, Suchart; Sunpasithiprasong Hospital, Medical Department<br>Hantrakun, Viriya; Mahidol Oxford Tropical Medicine Research Unit, Microbiology<br>Siriboon, Suwatthiya; Sunpasithiprasong Hospital<br>Boonsri, Chaiyaporn ; Sunpasithiprasong Hospital, Emergency Department<br>Poomthong, Pulyamon; Sunpasithiprasong Hospital<br>Singkaew, Bung-Orn; Sunpasithiprasong Hospital<br>Wasombat, Oratai; Sunpasithiprasong Hospital<br>Chamnan, Parinya; Sunpasithiprasong Hospital<br>Champunot, Ratapum; Buddhachinaraj Phitsanulok Hospital, Department of Internal Medicine<br>Rudd, Kristina; University of Pittsburgh, Department of Critical Care Medicine<br>Day, Nicholas; Mahidol Oxford Tropical Medicine Research Unit; Univerity of Oxford Nuffield Department of Medicine, Centre for Tropical Medicine<br>Dondorp, Arjen; Mahidol Oxford Tropical Medicine Research Unit; Univerity of Oxford Nuffield Department of Medicine, Centre for Tropical Medicine<br>Teparrukkul, Prapit; Sunpasithiprasong Hospital<br>West, Timothy Eoin; University of Washington, Division of Pulmonary, Critical Care, and Sleep Medicine; Mahidol University Faculty of Tropical Medicine, Department of Microbiology and Immunology<br>Limmathurotsakul, Direk; Mahidol Oxford Tropical Medicine Research Unit; Mahidol University Faculty of Tropical Medicine, Department of Tropical Hygiene

**Primary Subject Heading**:	Intensive care
Secondary Subject Heading:	Infectious diseases, Epidemiology
Keywords:	Epidemiology < INFECTIOUS DISEASES, INFECTIOUS DISEASES, INTENSIVE & CRITICAL CARE

For peer review only - http://bmjopen.bmj.com/site/about/guidelines.xhtml

I, the Submitting Author has the right to grant and does grant on behalf of all authors of the Work (as defined in the below author licence), an exclusive licence and/or a non-exclusive licence for contributions from authors who are: i) UK Crown employees; ii) where BMJ has agreed a CC-BY licence shall apply, and/or iii) in accordance with the terms applicable for US Federal Government officers or employees acting as part of their official duties; on a worldwide, perpetual, irrevocable, royalty-free basis to BMJ Publishing Group Ltd ("BMJ") its licensees and where the relevant Journal is co-owned by BMJ to the co-owners of the Journal, to publish the Work in this journal and any other BMJ products and to exploit all rights, as set out in our licence.

The Submitting Author accepts and understands that any supply made under these terms is made by BMJ to the Submitting Author unless you are acting as an employee on behalf of your employer or a postgraduate student of an affiliated institution which is paying any applicable article publishing charge ("APC") for Open Access articles. Where the Submitting Author wishes to make the Work available on an Open Access basis (and intends to pay the relevant APC), the terms of reuse of such Open Access shall be governed by a Creative Commons licence – details of these licences and which Creative Commons licence will apply to this Work are set out in our licence referred to above.

Other than as permitted in any relevant BMJ Author's Self Archiving Policies, I confirm this Work has not been accepted for publication elsewhere, is not being considered for publication elsewhere and does not duplicate material already published. I confirm all authors consent to publication of this Work and authorise the granting of this licence.

For peer review only

Effectiveness of a sepsis programme in a resource-limited setting

Suchart Booraphun MD[1], Viriya Hantrakun PhD[2], Suwatthiya Siriboon MD[1], Chaiyaporn Boonsri MD[1], Pulyamon Poomthong MD[1], Bung-Orn Singkaew BNS[1], Oratai Wasombat BNS[1], Parinya Chamnan MD, PhD[1], Ratapum Champunot MD[3], Kristina Rudd MD, MPH[4, 5], Nicholas PJ. Day[2,6] MD[2], PhD, Arjen M. Dondorp MD, PhD[2,6], Prapit Teparrukkul MD[1], T. Eoin West MD, MPH[5,7], Direk Limmathurotsakul MD, PhD[2,6,8]

Short Title: Sepsis Fast Track Thailand

[1]Sunpasitthiprasong Hospital, Ubon Ratchathani, Thailand

[2]Mahidol Oxford Tropical Medicine Research Unit, Faculty of Tropical Medicine, Mahidol University, Bangkok, Thailand

[3]Department of Internal Medicine, Buddhachinaraj Phitsanulok Hospital, Phitsanulok, Thailand

[4]Department of Critical Care Medicine, University of Pittsburgh, Pittsburgh, Pennsylvania, USA

[5]Division of Pulmonary, Critical Care, and Sleep Medicine, University of Washington, Seattle, United States

[6]Centre for Tropical Medicine and Global Health, Nuffield Department of Medicine, University of Oxford, Churchill Hospital, Oxford, United Kingdom

[7]Department of Microbiology and Immunology, Faculty of Tropical Medicine, Mahidol University, Bangkok, Thailand

For peer review only - http://bmjopen.bmj.com/site/about/guidelines.xhtml

[8]Department of Tropical Hygiene, Faculty of Tropical Medicine, Mahidol University, Bangkok, Thailand

***Corresponding author:** Assoc. Prof. Direk Limmathurotsakul, Mahidol-Oxford Tropical Medicines Research Unit, Faculty of Tropical Medicine, Mahidol University, 420/6 Rajvithi Road, Bangkok, 10400, Thailand. Tel: +66 2 203 6333, E-mail: direk@tropmedres.ac (DL)

Word count: abstract 300, text 3,829

Number of pages: 32, Tables: 2; Figures: 1, Supporting Information: 1

Keywords: sepsis, sepsis care, sepsis management, mortality, resource limited setting

For peer review only

For peer review only - http://bmjopen.bmj.com/site/about/guidelines.xhtml

Abstract

Objective: To evaluate the effectiveness of a Sepsis Fast Track (SFT) programme initiated at a regional referral hospital in Thailand in January 2015

Design: A retrospective analysis using the data of a prospective observational study (Ubon-sepsis) from March 2013 to January 2017

Setting: General medical wards and medical intensive care units (ICUs) of a study hospital

Participants: Patients with community-acquired sepsis observed under the Ubon-sepsis cohort. Sepsis was defined as modified Sequential Organ Failure Assessment (SOFA) score ≥ 2.

Main exposure: The SFT programme was a protocol to identify and initiate sepsis care on hospital admission, implemented at the study hospital in 2015. Patients in the SFT programme were admitted directly to the ICUs when available. The non-exposed group comprised of patients who received standard of care.

Main outcome: The primary outcome was 28-day mortality. The secondary outcomes were measured sepsis management interventions.

Results: Of 3,806 sepsis patients, 903 (24%) were detected and enrolled in the SFT programme of the study hospital (SFT group) and 2,903 received standard of care (non-exposed group). Patients in the SFT group had more organ dysfunction, were more likely to receive measured sepsis management and to be admitted directly to the ICU (19% vs. 4%). Patients in the SFT group were more likely to survive (adjusted hazard ratio 0.72; 95% CI 0.58 to 0.88, p=0.001) adjusted for admission year, gender, age, comorbidities, modified SOFA score and direct admission to the ICUs. The benefit of the SFT programme was not influenced by direct admission to the ICUs (p=0.44).

For peer review only - http://bmjopen.bmj.com/site/about/guidelines.xhtml

Conclusions: The SFT programme is associated with improved sepsis care and lower risk of death in sepsis patients in rural Thailand, where some critical care resources are limited. The survival benefit is observed even when patients enrolled in the programme could not be admitted directly into the ICUs.

Study registration number: NCT02217592

For peer review only

Strengths and limitations of this study

- The study hospital utilized the published framework, SCAN-TEACH-TREAT programme to develop a context specific quality of care improvement for sepsis in a tropical resource-limited setting.
- The study took advantage of a robust prospective observational study design that strengthened causal inference by providing pre-intervention information, having an appropriate control group from both pre and post-intervention periods, and controlling important confounding factors (i.e. the modified SOFA score).
- We found that most measured sepsis interventions increased and that admission to the ICUs were not associated with the survival benefit of the sepsis programme.
- The study did not record dosage of dobutamine, dopamine, epinephrine and norepinephrine, arterial blood gases were rarely performed, and the modified SOFA score (maximum 23) may be lower than the SOFA score (maximum 24).
- The observational study may have residual confounding factors such as improvement of care and profile of organ failure recognition overtimes.

For peer review only - http://bmjopen.bmj.com/site/about/guidelines.xhtml

INTRODUCTION

Sepsis is defined as life-threatening organ dysfunction caused by a dysregulated host response to infection,[1] and is the primary cause of death from infection, especially if not recognized and treated promptly.[2-4] Sepsis is a major cause of health loss worldwide and is associated with approximately eleven million deaths each year, most of which occur in low and middle-income countries (LMICs).[5] The United Nations World Health Assembly has recognized sepsis as a global health priority and adopted a resolution on improving its worldwide prevention, diagnosis and management.[6] Comprehensive guidelines such as those developed by the Surviving Sepsis Campaign have been associated with reduced mortality in high-income countries,[2-4] but effectiveness of these guidelines in LMICs needs more evaluation.[7-10]

Following the Surviving Sepsis Campaign (SSC) 2012,[11] the Ministry of Public Health Thailand and the Thai Society of Critical Care Medicine developed local recommendations on sepsis based on resource availability and local context.[12] The recommendations suggest that secondary-care and tertiary-care hospitals in the country should develop a Sepsis Fast Track (SFT) so that, on presentation, sepsis patients can be identified, treated and directly admitted to the ICUs when available. One small retrospective study showed lower mortality among sepsis patients enrolled than those not enrolled in the SFT (21% vs. 43%) at the study hospital,[13] while another study did not find an association between SFT and mortality outcome.[14] These studies were subject to selection biases due to their retrospective nature.[13-14] Interventional studies to randomize patients to receive or not receive the SFT, however, would be unethical and impractical after the national recommendations have been implemented. It is increasingly recommended to evaluate the impact

For peer review only - http://bmjopen.bmj.com/site/about/guidelines.xhtml

of healthcare interventions using routine data, particularly when a wide range of routinely collected data is available.[15]

Here, we analysed data from our prospective observational study of community-acquired sepsis patients presenting to a referral hospital in Thailand over four years (from March 2013 to January 2017)[16-17] to retrospectively evaluate the effectiveness of a SFT programme which was implemented at the study hospital in January 2015.

MATERIAL AND METHODS

Study design

We conducted a retrospective study to evaluate the effectiveness of the SFT programme by using the data of a prospective observational study (Ubon-sepsis).[16-17] The SFT programme was implemented at the study hospital in January 2015. The SFT programme at the study hospital included (1) diagnostic criteria for attending physicians and medical teams to systematically identify sepsis patients on hospital admission (Supplementary Table 1), (2) a recommended sepsis care protocol and (3) direct admission to the ICUs when available. The SFT programme at the study hospital was generated by the SFT committee of the study hospital (S.B., S.S., C.B., P.P., B.S., O.W., P.C. and P.T.) based on SSC 2012,[11] resource availability and local context.[12]

Details of the Ubon-sepsis cohort have been published elsewhere.[16-17] In short, the Ubon-sepsis research team, who were not attending physicians or medical teams at the study hospital, conducted a prospective observational study of community-acquired infections and sepsis from

For peer review only - http://bmjopen.bmj.com/site/about/guidelines.xhtml

March 2013 to January 2017.[16-17] The research team prospectively enrolled adult patients ≥ 18 years old who were admitted to the general medical wards and medical intensive care units (ICUs) with a primary diagnosis of infection made by the attending physician, were within 24 hours of admission to the study hospital, and had three of 20 systemic manifestations of infection documented in the medical records (Supplementary Table 2). The 20 systemic manifestations of the infections were consolidated from the 22 variables proposed as diagnostic criteria for sepsis for SSC 2012.[11] The study team sequentially screened all medical patients by reviewing admission logs in the emergency department (ED), medical wards, and medical ICUs twice daily (morning and afternoon) on each working day. The Ubon-sepsis cohort was initiated in 2012 prior to the implementation of SFT at the study hospital. The research team was not involved in any clinical interventions; enrollment in the SFT programme and all medical treatment was performed by attending physicians and medical teams. The research team did not adjust the study protocol, inclusion criteria and exclusion criteria of the Ubon-sepsis cohort during the entire study period, and the research team recorded whether participants in the Ubon-sepsis cohort were enrolled in the SFT programme.

The reporting of this study follows the STROBE guidelines. Written, informed permission was obtained from participants prior to enrollment in the Ubon-sepsis cohort.

Participants

For this study, we evaluated patients who were included into the Ubon-sepsis cohort and had community-acquired sepsis. Sepsis was defined as an infection with organ dysfunction in

For peer review only - http://bmjopen.bmj.com/site/about/guidelines.xhtml

accordance with the 2016 international Consensus (Sepsis-3) guidelines for sepsis.[1] Organ dysfunction was determined by a modified sequential (sepsis-based) organ failure assessment (SOFA) score ≥2 as previously described.[16-17] The study was conducted in 2013 prior to the Sepsis-3 definition, and inotropic and vasopressor agent doses were not recorded into the CRF.[1, 18] For the cardiovascular component of the SOFA score, the scoring was modified such that subjects were scored a maximum of 2 (on a 4-point scale) if they received only dobutamine or dopamine, and scored a maximum of 3 if they received epinephrine or norepinephrine. For the respiratory component of the SOFA score, as PaO2/FiO2 indices were not available for the majority of subjects due to infrequency of arterial blood gas tests, the score was modified as follows: Subjects were scored a maximum of 2 (4-point scale) if they received advanced respiratory support (endotracheal tube, gas powered or electrical powered mechanical ventilation) and arterial blood gas test was not performed.[16-17] The Ubon-sepsis cohort excluded patients who were suspected of having hospital-acquired infections (determined by the attending physician), hospitalized within 30 days prior to the current admission, or hospitalized at any facility for a total duration longer than 72 hours prior to enrollment.

Main exposure

Main exposure of the study was the SFT programme. All patients included in the Ubon-sepsis cohort from March 2013 to December 2014 who received standard care were considered as the non-exposed group. Patients included in the Ubon-sepsis cohort from January 2015 to January 2017 who received standard care or were received care in the SFT programme by attending medical teams using their criteria on admission (Supplementary Table 1) were considered as the

For peer review only - http://bmjopen.bmj.com/site/about/guidelines.xhtml

additional non-exposed group or as the SFT group, respectively. The Ubon-sepsis research team were not involved in decision-making regarding enrollment to the SFT programme.

Patients in the non-exposed group received standard care according to local guidelines. Patients in the SFT group received the standard of care along with a recommended sepsis care protocol of the SFT programme. First, preprinted recommended doctor orders for the SFT programme were used as of January 2015 (Supplementary Figure 1). The recommended orders included oxygen administration, intravenous fluid loading and fluid administration to achieve the recommended target of 30 mL/kg crystalloid, blood culture, recommended stat (immediate) doses and choices of parenteral antibiotics including ceftriaxone, ceftazidime, cloxacillin, metronidazole and gentamycin, contact ICU for ICU admission (if available), oxygen supplementation, close monitoring of vital signs and urine output, and a set of diagnostic tests including chest radiography, electrocardiogram, rapid blood glucose test, serum lactate, complete blood count, blood urea nitrogen, creatinine, electrolytes, liver function tests, albumin level, prothrombin time and partial thromboplastin time. Second, as of March 2016, the resuscitation workflow to normalize and maintain a mean arterial pressure (MAP) ≥65 mmHg, systolic blood pressure (SBP) ≥90 mmHg and urine output ≥0.5 mL/kg/hr within the first six hours was formally implemented and recommended (Supplementary Figure 2). The resuscitation workflow included fluid resuscitation, measurement of central venous pressure (CVP) and central venous oxygen saturation ($SCVO_2$), administration of adrenergic agents, blood transfusion for haematocrit <30% and hydrocortisone if adequate fluid resuscitation and vasopressor therapy could not restore hemodynamic stability. The resuscitation workflow was pre-printed and included in the clinical chart of every SFT patient

For peer review only - http://bmjopen.bmj.com/site/about/guidelines.xhtml

(together with pre-printed doctor's orders), and was recommended even if patients could not be admitted directly to the ICU. A separate set of documents, recommended management and recommended frequency of vital signs monitoring for nurses (i.e. nurse notes for SFT patients) were also used for every SFT patient. Preparation and regular meetings to implement and monitor the SFT programme were organized by the SFT committee of Sunpasitthiprasong Hospital.

Outcome measures

The primary outcome measure was 28-day mortality as recorded in the Ubon-sepsis cohort.[16] 28-day mortality data were collected via telephone contact if subjects were no longer hospitalized and had been discharged alive.[16] The secondary outcome measures were sepsis management interventions; including antibiotics administration, blood cultures, mechanical ventilation, adrenergic agents, acute haemodialysis and placement of a urinary catheter within the first day of hospitalization.[16-17]

Sample size

The sample size of the study was determined by the sample size of Ubon-sepsis cohort. We assumed that about 50% of 3,806 sepsis patients in the Ubon-sepsis cohort were enrolled after the implementation of the SFT programme, of which 50% were enrolled in the SFT programme (i.e. 952 and 2,854 patients were estimated to be the SFT and non-exposed group, respectively). We assumed that the mortality of the non-exposed group was 21% based on published data.[16-17] Our current sample size of 3,806 would provide a power of 80% at an alpha error of 5% to detect a 4% difference in the mortality outcome.

For peer review only - http://bmjopen.bmj.com/site/about/guidelines.xhtml

Statistical analysis

All sepsis patients were included in the analysis regardless of whether they were enrolled before or after the implementation of the SFT programme. We used the Chi-square test and Mann-Whitney test to compare the proportions of binary variables and median of continuous variables between groups, respectively. The interquartile range is presented as 25th and 75th percentiles.

In the primary analysis, we used multivariable Cox proportional hazard models to evaluate the effectiveness of SFT programme on 28-day mortality. The multivariable Cox proportional hazard model was used to adjust the difference between those receiving the SFT programme and the others.[19] To reduce bias in the model development, we used the previous multivariable Cox proportional hazard model as the base model,[16] added the SFT group variable and direct admission to the ICU, and modified by adding a time variable to represent possible changes over time and by using continuous modified SOFA score on admission rather than as a binary variable (modified SOFA score $\geq$2). Twenty eight patients enrolled in early 2017 were considered as enrolled in 2016. The continuous modified SOFA score was used to improve regression adjustment for disease severity of the model. The other variables included in the model were gender, age group, transfer from other hospital, comorbidities (diabetes mellitus, chronic kidney disease, liver disease and malignancy) and blood culture positive for pathogenic organisms. We tested potential predefined interactions between the SFT programme and direct admission to the ICUs. Using a conceptual framework, we also consider that admission directly to the ICU could also be caused by the SFT; therefore, we developed another multivariable model not including the variable for direct

For peer review only

For peer review only - http://bmjopen.bmj.com/site/about/guidelines.xhtml

admission to the ICU. The goodness of fit for the multivariable Cox proportional hazard model was tested with a Hosmer and Lemeshow test. For the Cox proportional hazard model, we assessed whether the hazard ratio was constant over time using Schoenfeld residuals.

For the secondary endpoints, we used multivariable logistic regression models with similar independent variables as the model for 28-mortality outcome and used each sepsis management process as an outcome. We estimated the total effect of the SFT on each sepsis management by using the multivariable logistic regression models adjusted for difference in characteristics and disease severity of the patients. This was because each sepsis management could be caused by characteristics of the patients, disease severity and the SFT.[20]

We also performed sensitivity analyses by excluding patients enrolled prior to the implementation of the SFT programme and by replacing direct admission to the ICUs with admission to the ICUs within the first hospital day. All analyses were performed with STATA 15.1 (StataCorp, College Station, TX, USA).

Patient and public involvement

No patients were involved in setting the research question or the outcome measures, nor were they involved in developing plans for recruitment, design, or implementation of the study. No patients were asked to advice on interpretation or writing the results. The results will be disseminated to the public through online social media.

For peer review only - http://bmjopen.bmj.com/site/about/guidelines.xhtml

RESULTS

Baseline characteristics

The observational cohort study (Ubon-sepsis) included 5,001 patients presenting with community-acquired infections from March 2013 to January 2017, and 12 patients were excluded due to unknown 28-day mortality outcome. 3,806 (76%) met criteria for sepsis within the first 24 hours of admission with a modified SOFA score ≥2, and were included for the analysis. Figure 1 shows the flow of participants through the study. Among 3,806 sepsis patients, 903 were enrolled in the SFT programme and considered as the SFT group, and 2,903 were not enrolled in the SFT programme, received standard of care, and considered as the non-exposed group. Of 2,903 sepsis patients in the non-exposed group, 1,636 were included in the observational cohort study prior to the implementation of SFT programme and 1,267 were after the implementation of the programme.

Table 1 shows the characteristics of the study patients. Patients in the SFT group were older and more likely to have underlying diseases of diabetes mellitus, cerebrovascular diseases and dyslipidemia. Patients included in the SFT group had higher severity of organ dysfunction determined by the modified SOFA score compared with the non-exposed group (median 6 [IQR 4-9] vs. 4 [IQR 3-6], p<0.001). A higher proportion of patients in the SFT group were admitted directly to the ICU compared with the non-exposed group (19% vs 5%, p<0.001).

For peer review only

For peer review only - http://bmjopen.bmj.com/site/about/guidelines.xhtml

Table 1| Baseline characteristics of sepsis patients enrolled in the Sepsis Fast Track programme[1] (SFT group) or standard of care (non-exposed group). Values are number (percentages) unless stated otherwise

Characteristics	SFT group[2] (n=903)	Non-exposed group[3] (n=2903)	P value
**Male gender**	526 (58%)	1653 (57%)	0.49
**Age (years) (median [IQR])**	63 (49-74)	56 (39-70)	<0.001
**Age group (years)**			
18-40	100 (11%)	647 (22%)	<0.001
>40-60	277 (31%)	875 (30%)	
>60-70	214 (24%)	513 (18%)	
>70	312 (35%)	868 (30%)	
**Comorbidities**			
Hypertension	239 (26%)	726 (25%)	0.38
Diabetes mellitus	213 (24%)	594 (20%)	0.05
Chronic kidney disease	129 (14%)	391 (13%)	0.53
Dyslipidemia	66 (7%)	152 (5%)	0.02
Heart disease	48 (5%)	183 (6%)	0.28
Lung disease	67 (7%)	239 (8%)	0.43
Liver disease	33 (4%)	91 (3%)	0.44
Cerebrovascular disease	29 (3%)	55 (2%)	0.02
Malignancy	13 (1%)	47 (2%)	0.71
Human immunodeficiency virus (HIV)	6 (1%)	33 (1%)	0.22
**Organ dysfunction**			
Modified SOFA score (median [IQR])	6 (4-9)	4 (3-6)	<0.001
Renal dysfunction[4]	706 (78%)	1846 (64%)	<0.001
Cardiovascular dysfunction[4]	811 (90%)	1532 (53%)	<0.001
Coagulation dysfunction[4]	419 (46%)	1562 (54%)	<0.001
Liver dysfunction[4]	311 (34%)	822 (28%)	0.001
Respiratory dysfunction[4]	337 (37%)	853 (29%)	<0.001
Central nervous system dysfunction[4]	166 (18%)	530 (18%)	0.92
**Transferred from other hospitals**	874 (97%)	2372 (84%)	<0.001
**Duration of symptoms (median [IQR])**	2 (1-3)	3 (1-5)	<0.001
≤ 2 days	505 (56%)	1191 (41%)	
3-7 days	362 (40%)	1488 (51%)	
> 7 days	36 (4%)	224 (8%)	

For peer review only
For peer review only - http://bmjopen.bmj.com/site/about/guidelines.xhtml

Characteristics	SFT group[2] (n=903)	Non-exposed group[3] (n=2903)	P value
**Presenting clinical syndromes[5]**			
Septic shock	687 (76%)	733 (25%)	<0.001
Acute febrile illness	206 (23%)	940 (32%)	<0.001
Lower respiratory infection	223 (25%)	890 (31%)	0.001
Sepsis	225 (25%)	273 (9%)	<0.001
Others	13 (1%)	456 (16%)	<0.001
Diarrheal illness	150 (17%)	264 (9%)	<0.001
**Admitted directly to an ICU upon admission**	170 (19%)	128 (4%)	<0.001
**Admitted to an ICU within 24 hours of admission**	270 (29%)	370 (13%)	<0.001
**Blood culture positive for pathogenic organisms**	175 (19%)	347 (12%)	<0.001
**Year**			
2013	N/A	1047 (26%)	<0.001
2014	N/A	1156 (29%)	
2015	369 (39%)	956 (24%)	
2016	556 (59%)	869 (21%)	
2017	14 (1%)	22 (1%)	

[1] SFT programme was implemented at the study hospital in January 2015.

[2] 903 patients of the Ubon-sepsis cohort were enrolled in SFT programme after the implementation of the SFT programme (Figure 1)

[3] Included 1,636 and 1,267 patients in the Ubon-sepsis cohort before and after the implementation of the SFT programme, respectively.

[4] Organ dysfunction defined as modified SOFA score was ≥1 for each organ system.[16]

[5] Patients may have more than one presenting clinical syndrome.

Primary outcomes

The primary outcome, mortality within 28 days, occurred in 205 of 903 (23%) in the SFT group and 574 of 2,903 (20%) in the non-exposed group. In the primary analysis, patients in the SFT group were more likely to survive adjusted for baseline characteristics, severity of sepsis and direct

For peer review only - http://bmjopen.bmj.com/site/about/guidelines.xhtml

287 admission the ICUs (adjusted hazard ratio [aHR] 0.72, 95% CI 0.58-0.88, p=0.001; Table 2). Older age, higher modified SOFA score, underlying disease of malignancy and chronic kidney disease, blood culture positive for pathogenic organisms and direct admission to the ICUs were associated with risk of mortality.

For peer review only

For peer review only - http://bmjopen.bmj.com/site/about/guidelines.xhtml

Table 2| Factors associated with 28-day mortality using multivariable Cox proportional hazards model

Variables	Died (n=779)	Survived (n=3027)	Adjusted hazard ratio[2] (95%CI)	P value
**SFT group[1]**	205 (26%)	698 (23%)	0.72 (0.58- 0.88)	0.001
**Male gender**	445 (57%)	698 (23%)	0.87 (0.75- 1.01)	0.06
**Age group (years) (n [%])**				
• 18-40	59 (8%)	688 (23%)	1	<0.001
• >40-60	222 (29%)	930 (31%)	1.72 (1.28- 2.30)	
• >60-70	159 (20%)	568 (19%)	2.10 (1.54- 2.86)	
• >70	339 (44%)	841 (28%)	3.41 (2.57- 4.53)	
**Transferred from other hospital**	715 (92%)	2595 (86%)	1.14 (0.88- 1.49)	0.33
**Modified SOFA score (median, IQR)**	6 (4-9)	4 (3-6)	1.23 (1.21- 1.26)	<0.001
**Comorbidities**				
• Diabetes mellitus	205 (26%)	602 (20%)	1.06 (0.90- 1.26)	0.47
• Chronic kidney disease	141 (18%)	379 (13%)	1.22 (1.01- 1.48)	0.04
• Liver disease	39 (5%)	85 (3%)	1.27 (0.91- 1.76)	0.16
• Malignancy	24 (3%)	36 (1%)	2.64 (1.75- 3.99)	<0.001
**Blood culture positive for pathogenic organisms**	190 (24%)	332 (11%)	1.83 (1.55- 2.17)	<0.001
**Year**				
• 2013	165 (21%)	637 (21%)	1	0.30
• 2014	183 (23%)	651 (22%)	1.03 (0.83- 1.27)	
• 2015	207 (27%)	808 (27%)	1.05 (0.84- 1.31)	
• 2016[2]	224 (29%)	931 (31%)	0.88 (0.70- 1.11)	
**Direct admission to the ICU**	128 (16%)	170 (6%)	1.68 (1.36- 2.06)	<0.001

[1] Enrolled in the Sepsis Fast Track (SFT) programme.

[2] The hazard ratio of SFT was adjusted for gender, age group, transferred from other hospital, modified SOFA score, comorbidities, blood culture positive for pathogenic organisms, year and direct admission to the ICU. The hazard ratios of other variable could be considered as the controlled direct effect of those variables.[20]

For peer review only - http://bmjopen.bmj.com/site/about/guidelines.xhtml

[2] Included 28 patients in 2017

In a pre-specified interaction test, we found consistency of the association between the SFT and lower risk of mortality when stratifying by direct admission to the ICUs (interaction test p=0.44). In the multivariable model including an interaction variable, the effect of SFT was comparable among those admitted directly to the ICUs (aHR 0.81, 95% CI 0.56 to 1.18) and those admitted directly to the general medical wards (aHR 0.69, 95% CI 0.56 to 0.86). Using a conceptual framework, we considered that admission directly to the ICU could also be caused by the SFT; therefore, we developed another multivariable model that did not adjust for direct admission to the ICU. We observed that the effect of the SFT was comparable (aHR 0.77, 95% CI 0.63 to 0.94; Supplementary Table 3).

Secondary outcomes

Using multivariable logistic regression models, we found that patients in the SFT group were more likely to receive most sepsis management interventions than patients in the non-exposed group adjusting for baseline characteristics and severity of sepsis (Supplementary Table 4). Those included antibiotics, blood cultures, adrenergic agents, and placement of a urinary catheter within the first day of hospitalization. However, sepsis patients in the SFT group were less likely to receive mechanical ventilation compared with those in the non-exposed group adjusting for baseline characteristics, severity of sepsis and direct admission to the ICUs group (adjusted odds ratio [aOR] 0.30; 95% CI 0.24 to 0.38). We found that direct admission to the ICUs (aOR 5.77,

For peer review only - http://bmjopen.bmj.com/site/about/guidelines.xhtml

95% CI 4.20 to 7.92) and transfer from other hospitals (aOR 3.45, 95% CI 2.42 to 4.91) were strongly associated with the requirement of mechanical ventilation.

Sensitivity Analyses

We performed a sensitivity analysis by excluding the 1,636 patients enrolled in the observational study prior to the implementation of SFT programme. Similar differences in baseline characteristics were observed when comparing 903 patients in the SFT group to the 1,267 patients in the non-exposed group enrolled after the implementation of the SFT programme (Supplementary Table 5). A higher chance of survival in the SFT group compared to the non-exposed group was also observed (aHR 0.68, 95% CI 0.55 to 0.84, p<0.001; Supplementary Table 6). There was no interaction between the management intervention and direct admission to the ICUs (p=0.92).

We also performed another sensitivity analysis by replacing direct admission to the ICUs with admission to the ICUs within the first hospital day. Of 3,806 patients, 640 (17%) were admitted to the ICUs within the first day of admission. A higher chance of survival in the SFT group compared to the non-exposed group was also observed (aHR 0.72, 95% CI 0.59 to 0.88, p=0.002). There was also no interaction between the management intervention and admission to the ICUs within the first day of admission (p=0.29).

For peer review only - http://bmjopen.bmj.com/site/about/guidelines.xhtml

DISCUSSION

In this study evaluating patients with community-acquired sepsis, enrollment into a programme to identify and initiate sepsis care implemented at the study hospital (SFT programme) was associated with 28% lower risk of mortality. In recent years, there has been an increasing need to understand benefit and cost effectiveness of implementation of sepsis care interventions in LMICs because of concerns that international sepsis guidelines[11] may not be extrapolated to patients with tropical infectious diseases[7-9] and to resource-limited settings with poor ICU capacity.[10] In this study we show the effectiveness of sepsis protocol modified based on resource availability in a tropical country, where causes of community-acquired sepsis include malaria and tropical viral diseases.[16, 21-22] Access to the ICU increased after the implementation of the SFT programme, but the majority of sepsis patients were still managed on the general wards, including those with respiratory failure or shock. Nonetheless, our study shows that enhancing sepsis care in the emergency department and general medical wards, as well as improving access to ICUs can reduce sepsis mortality in a LMIC.

The lower odds of receiving mechanical ventilation in the SFT group could be a sign of improved sepsis care. Patients in the SFT group are monitored closely either in or outside the ICUs, and the attending physicians aim to obviate the need for airway intubation when possible.[7] Attending physicians may tend to provide mechanical ventilation to patients in the non-exposed group based on broad indications such as (1) airway protection, (2) hypercapnic respiratory failure, (3) hypoxemic respiratory failure or (4) circulatory failure[23-24] because they may not be able to monitor patients' breathing and oxygen saturation as often as those enrolled in the SFT programme.

For peer review only - http://bmjopen.bmj.com/site/about/guidelines.xhtml

It is not surprising that patients in the SFT group had more organ dysfunction than those in the non-exposed group. This is because the severity of organ dysfunction among patients with septic shock, respiratory failure and alteration of conscious can be assessed clinically on admission, and those patients could be enrolled in the SFT programme when the laboratory test results were not yet available. However, the non-exposed group were defined as having sepsis based on clinical findings and all laboratory test results within 24 hours of admission (per protocol of Ubon-sepsis cohort study[16-17]). Therefore, the non-exposed group could use laboratory test results (i.e. liver function tests, creatinine level, international normalised ratio and activated partial thromplastin time) from blood specimens drawn on admission. Therefore, the SFT programme were more likely to enroll patients with obvious signs of sepsis and septic shock; such as acute respiratory failure and hypotension, while Ubon-sepsis cohort could include sepsis patients with relatively lower modified SOFA scores.

Comparison with other studies

Our study is not the first to evaluate effectiveness of sepsis intervention in LMICs. Early recognition and protocol directed intervention improves outcomes of sepsis in adults[25-27] and severe infection in children[28] in LMICs. The optimal method of fluid resuscitation in sepsis in tropical LMICs has not been determined.[8, 25, 29-30] Our resuscitation protocol is a simple guideline, and the SFT recommend doctors to be careful and adjust fluid resuscitation based on preliminary diagnoses, underlying diseases and rapid diagnostic test results (i.e. if sepsis is caused by malaria or dengue infection). The implementation of the SFT programme in our study hospital and in

For peer review only - http://bmjopen.bmj.com/site/about/guidelines.xhtml

Thailand is consistent with the recommendation of "SCAN-TEACH-TREAT" programme developed by Sepsis in Resource-Limited Settings Workgroup of the Surviving Sepsis Campaign.[7] The SFT programme evaluated resources in the setting (SCAN component), focused on educational interventions on early recognition and management of sepsis among medical personnel including physicians, nurses and students (TEACH component) and implemented pragmatic and simple bundles into practice (TREAT component). In addition, the SFT programme has the strong support and endorsement of local health and governmental leaders.[12]

Strength and limitations of the study

This study features four strengths. First, the study hospital utilized the published framework, SCAN-TEACH-TREAT programme to develop a context specific quality of care improvement for sepsis,[7] and we closely monitor and evaluate the effectiveness of an intervention. Second, the study took advantage of a robust prospective observational study design that strengthened causal inference by providing pre-intervention information, having an appropriate non-exposed group from both pre and post-intervention periods, and controlling important confounding factors (i.e. the modified SOFA score) which were measured systematically throughout the study period. Third, this study incorporated several predictors of interest (measured sepsis management interventions and admission to the ICUs). This allows us to identify that most measured sepsis interventions increased and that admission to the ICUs were not associated with the survival benefit of the SFT programme. Fourth, the focus on sepsis at a public tertiary-care hospital in Thailand helped us to estimate the effect of sepsis protocol in a tropical resource-limited setting with large sample size.

For peer review only - http://bmjopen.bmj.com/site/about/guidelines.xhtml

Our study had several limitations. First, a modified SOFA score was used because the dosage of dobutamine, dopamine, epinephrine and norepinephrine were not recorded and arterial blood gases were rarely performed. The modified SOFA score (maximum 23) may be lower than the SOFA score (maximum 24). Nonetheless, the modified SOFA score is strongly associated with mortality in sepsis.[16-17] Second, the proportional hazards assumption was met for all variables, including the main variable (the SFT), except one controlled variable (the modified SOFA score). The adjusted effect estimates could be under or overestimated due to residual confounding factors such as improvement of care and profile of organ failure recognition overtimes.

Conclusions and future implications

Our study successfully demonstrated effectiveness of a sepsis programme implemented in a LMIC. Measuring effectiveness of a sepsis programme is a complex issue, and we utilized a data of a prospective observational study and carefully controlled for severity of sepsis and temporal trends in our analyses. Care in sepsis patients improved after the implementation of the programme. Additional research is needed to better understand cost of the intervention, long-term benefits and impact of the programme on a national scale. National strategies aimed at saving lives from sepsis in LMICs should be encouraged. Such strategies should include analysis of resources and local circumstances, followed by development, implementation and assessment of customized programmes.

For peer review only

For peer review only - http://bmjopen.bmj.com/site/about/guidelines.xhtml

Acknowledgement:

We thank all patients, their relatives, and staff of the Sunpasitthiprasong hospital who participated in the study. We thank Mayura Malasit, Praweennuch Watanachaiprasert, Chayamon Krainoonsing, Passaraporn Kesaphun, Nannicha Jirapornuwat, Gumphol Wongsuvan, Areeya Faosap, Yaowaret Dokket, Sukhumal Pewlaorng, Jintana Suwannapruek, Prapass Wannapinij and Diane Tomita for their clinical, laboratory and administrative support.

Contributors:

NPJD, TEW, and DL obtained grant funding. SB, VH, PT, TEW, and DL contributed to study conception development and study design. SB, VH, PT, TEW and DL contributed to study conduct, data collection, and study administration. VH and DL performed the statistical analysis and interpreted the data and had full access to all of the data in the study. Both authors can take responsibility for the integrity of the data and the accuracy of the data analysis. DL is a guarantor. SB, VH, PT, TEW, and DL wrote the first draft of a manuscript, with input from SS, CB, PC, KR, and AD. PP, BS, OW, and RC provided scientific or administrative support. All authors contributed to results interpretation, critically revised, and approved the final submitted manuscript. The corresponding author attests that all listed authors meet authorship criteria and that no others meeting the criteria have been omitted.

Funding:

The study was funded by the Wellcome Trust (090219/Z/09/Z) and National Heart, Lung and Blood Institute, National Institutes of Health (R01HL113382). DL is supported by an intermediate

For peer review only

For peer review only - http://bmjopen.bmj.com/site/about/guidelines.xhtml

fellowship from the Wellcome Trust (101103/Z/13/Z). The funders had no role in the design and conduct of the study, all study procedures, data collection, data analyses, data interpretation, writing of the report, and the decision to submit the article for publication.

Competing interests:

The authors declare that they have no completing interests.

Ethics approvals:

The study was conducted the study in full compliance with the principles of good clinical practice (GCP), and the ethical principles of the Declaration of Helsinki. The study protocol and related documents were approved by Sunpasitthiprasong Hospital Ethics Committee (039/2556), the Ethics Committee of the Faculty of Tropical Medicine, Mahidol University (MUTM2012-024-01), the University of Washington Institutional Review Board (42988) and the Oxford Tropical Research Ethics Committee at the University of Oxford (OXTREC172-12). Signed or fingerprinted informed consent was obtained from the participants or their representatives before enrollment.

Data sharing:

The final database with the data dictionary are publicly available online https://doi.org/10.6084/m9.figshare.12102627.

The lead authors (SB, VH and DL) affirm that the manuscript is an honest, accurate, and transparent account of the study being reported; that no important aspects of the study have been

For peer review only - http://bmjopen.bmj.com/site/about/guidelines.xhtml

omitted; and that any discrepancies from the study as planned have been explained. This is an Open Access article distributed in accordance with the terms of the Creative Commons Attribution (CC BY 4.0) license, which permits unrestricted reuse, distribution, and reproduction in any medium, provided the original work is properly cited. See: http://creativecommons.org/licenses/by/4.0/

Dissemination to participants and related patient and public communities:

The results of this study will be disseminated to physicians at the study hospital, health care providers, policy makers, and academic communities through various mediums, including printed report, internal hospital meetings, academic conferences, and institutional networks. The results from this study will be used to inform the current Sepsis Fast Track programme at Sunpasitthiprasong hospital and the community hospitals which are located in jurisdiction of the study hospital catchment areas in the Northeast Thailand. The study results will not be disseminated to patients or general population, because study results are in medical context.

For peer review only

For peer review only - http://bmjopen.bmj.com/site/about/guidelines.xhtml

References

1 Singer M, Deutschman CS, Seymour CW, et al. The Third International Consensus Definitions for Sepsis and Septic Shock (Sepsis-3). *JAMA* 2016;315(8):801-10. doi: 10.1001/jama.2016.0287

2 Rhodes A, Evans LE, Alhazzani W, et al. Surviving Sepsis Campaign: International Guidelines for Management of Sepsis and Septic Shock 2016. *Crit Care Med* 2017;45(3):486-552. doi: 10.1097/ccm.0000000000002255

3 WHO. WHO Sepsis Technical Expert Meeting - Meeting report Geneva: World Health Organization; 2018 [Available from: https://www.who.int/servicedeliverysafety/areas/sepsis_meeting-report-2018.pdf accessed 25 November 2019 2019].

4 Vincent J-L. The Clinical Challenge of Sepsis Identification and Monitoring. *PLoS Med* 2016;13(5):e1002022. doi: 10.1371/journal.pmed.1002022

5 Rudd KE, Johnson SC, Agesa KM, et al. Global, regional, and national sepsis incidence and mortality, 1990–2017: analysis for the Global Burden of Disease Study. *The Lancet* 2020;395(10219):200-11. doi: 10.1016/S0140-6736(19)32989-7

6 Reinhart K, Daniels R, Kissoon N, et al. Recognizing Sepsis as a Global Health Priority — A WHO Resolution. *N Engl J Med* 2017;377(5):414-17. doi: 10.1056/NEJMp1707170

For peer review only

For peer review only - http://bmjopen.bmj.com/site/about/guidelines.xhtml

7 Kwizera A, Baelani I, Mer M, et al. The long sepsis journey in low- and middle-income countries begins with a first step...but on which road? *Crit Care* 2018;22(1):64. doi: 10.1186/s13054-018-1987-z

8 McGloughlin S, Richards GA, Nor MBM, et al. Sepsis in tropical regions: Report from the task force on tropical diseases by the World Federation of Societies of Intensive and Critical Care Medicine. *J Crit Care* 2018;46:115-18. doi: 10.1016/j.jcrc.2017.12.018

9 Becker JU, Theodosis C, Jacob ST, et al. Surviving sepsis in low-income and middle-income countries: new directions for care and research. *Lancet Infect Dis* 2009;9(9):577-82. doi: 10.1016/S1473-3099(09)70135-5

10 Schultz MJ, Dunser MW, Dondorp AM, et al. Current challenges in the management of sepsis in ICUs in resource-poor settings and suggestions for the future. *Intensive Care Med* 2017;43(5):612-24. doi: 10.1007/s00134-017-4750-z

11 Dellinger RP, Levy MM, Rhodes A, et al. Surviving Sepsis Campaign: international guidelines for management of severe sepsis and septic shock, 2012. *Intensive Care Med* 2013;39(2):165-228. doi: 10.1007/s00134-012-2769-8

12 Office of permanent secretatry, Ministry of Public Health, Thailand. The Development of Health Service Plan and Indicators of Health Outcomes 2018:8.

13 Ruangchan S, Chusri S, Saengsanga P, et al. Clinical Outcomes of Community-Acquired Severe Sepsis after Implementation of a Simple Severe Sepsis Fast Track. *J Med Assoc Thai* 2016;99(8):877-85.

For peer review only

For peer review only - http://bmjopen.bmj.com/site/about/guidelines.xhtml

14 Ittisanyakorn M, Ruchichanantakul S, Vanichkulbodee A, et al. Prevalence and factors associated with one-year mortality of infectious diseases among elderly emergency department patients in a middle-income country. *BMC Infect Dis* 2019;19(1):662. doi: 10.1186/s12879-019-4301-z

15 Clarke GM, Conti S, Wolters AT, et al. Evaluating the impact of healthcare interventions using routine data. *BMJ* 2019;365:l2239. doi: 10.1136/bmj.l2239

16 Hantrakun V, Somayaji R, Teparrukkul P, et al. Clinical epidemiology and outcomes of community acquired infection and sepsis among hospitalized patients in a resource limited setting in Northeast Thailand: A prospective observational study (Ubon-sepsis). *PLoS One* 2018;13(9):e0204509. doi: 10.1371/journal.pone.0204509

17 Rudd KE, Hantrakun V, Somayaji R, et al. Early management of sepsis in medical patients in rural Thailand: a single-center prospective observational study. *J Intensive Care* 2019;7:55. doi: 10.1186/s40560-019-0407-z

18 Seymour CW, Liu VX, Iwashyna TJ, et al. Assessment of Clinical Criteria for Sepsis: For the Third International Consensus Definitions for Sepsis and Septic Shock (Sepsis-3). *Jama* 2016;315(8):762

19 Craig P, Cooper C, Gunnell D, et al. Using natural experiments to evaluate population health interventions: new Medical Research Council guidance. *J Epidemiol Community Health* 2012;66(12):1182-6. doi: 10.1136/jech-2011-200375

20 Westreich D, Greenland S. The Table 2 Fallacy: Presenting and Interpreting Confounder and Modifier Coefficients. *Am J Epidemiol* 2013;177(4):292-98. doi: 10.1093/aje/kws412

For peer review only

For peer review only - http://bmjopen.bmj.com/site/about/guidelines.xhtml

21 Teparrukkul P, Hantrakun V, Imwong M, et al. Utility of qSOFA and modified SOFA in severe malaria presenting as sepsis. *PLoS One* 2019;14(10):e0223457. doi: 10.1371/journal.pone.0223457

22 Teparrukkul P, Hantrakun V, Day NPJ, et al. Management and outcomes of severe dengue patients presenting with sepsis in a tropical country. *PLoS One* 2017;12(4):e0176233. doi: 10.1371/journal.pone.0176233

23 Pierson DJ. Indications for mechanical ventilation in adults with acute respiratory failure. *Respir Care* 2002;47(3):249-62; discussion 62-5.

24 Pham T, Brochard LJ, Slutsky AS. Mechanical Ventilation: State of the Art. *Mayo Clin Proc* 2017;92(9):1382-400. doi: 10.1016/j.mayocp.2017.05.004

25 Jacob ST, Banura P, Baeten JM, et al. The impact of early monitored management on survival in hospitalized adult Ugandan patients with severe sepsis: a prospective intervention study*. *Crit Care Med* 2012;40(7):2050-8. doi: 10.1097/CCM.0b013e31824e65d7

26 Machado FR, Ferreira EM, Schippers P, et al. Implementation of sepsis bundles in public hospitals in Brazil: a prospective study with heterogeneous results. *Crit Care* 2017;21(1):268. doi: 10.1186/s13054-017-1858-z

27 Noritomi DT, Ranzani OT, Monteiro MB, et al. Implementation of a multifaceted sepsis education program in an emerging country setting: clinical outcomes and cost-effectiveness in a long-term follow-up study. *Intensive Care Med* 2014;40(2):182-91. doi: 10.1007/s00134-013-3131-5

For peer review only - http://bmjopen.bmj.com/site/about/guidelines.xhtml

28 Sazawal S, Black RE, Pneumonia Case Management Trials G. Effect of pneumonia case management on mortality in neonates, infants, and preschool children: a meta-analysis of community-based trials. *Lancet Infect Dis* 2003;3(9):547-56. doi: 10.1016/s1473-3099(03)00737-0

29 Maitland K, Kiguli S, Opoka RO, et al. Mortality after fluid bolus in African children with severe infection. *N Engl J Med* 2011;364(26):2483-95. doi: 10.1056/NEJMoa1101549

30 Andrews B, Semler MW, Muchemwa L, et al. Effect of an Early Resuscitation Protocol on In-hospital Mortality Among Adults With Sepsis and Hypotension: A Randomized Clinical Trial. *JAMA* 2017;318(13):1233-40. doi: 10.1001/jama.2017.10913

For peer review only - http://bmjopen.bmj.com/site/about/guidelines.xhtml

582 **Figure legends**

583 **Figure 1| Flow of participants through study**

584 **Footnote of figure 1:** This study used the data of an observational study on sepsis patients (Ubon-
585 sepsis) from March 2013 to January 2017 to evaluate the effectiveness of a Sepsis Fast Track
586 (SFT) programme implemented at the study hospital in January 2015

587

For peer review only

For peer review only - http://bmjopen.bmj.com/site/about/guidelines.xhtml

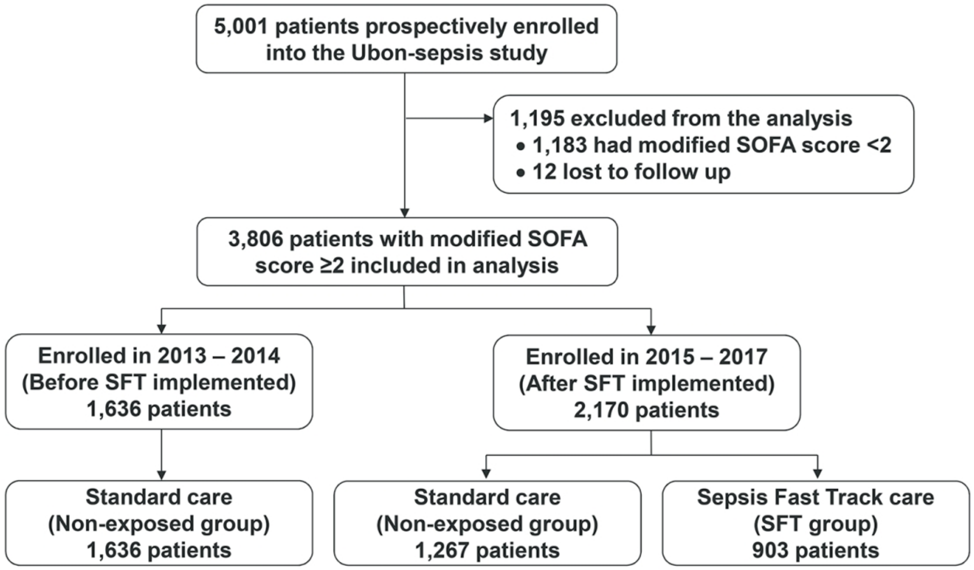

Figure 1| Flow of participants through study

Effectiveness of a sepsis programme in a resource-limited setting

Suchart Booraphun MD[1], Viriya Hantrakun PhD[2], Suwatthiya Siriboon MD[1], Chaiyaporn Boonsri MD[1], Pulyamon Poomthong MD[1], Bung-Orn Singkaew BNS[1], Oratai Wasombat BNS[1], Parinya Chamnan MD, PhD[1], Ratapum Champunot MD[3], Kristina Rudd MD, MPH[4, 5], Nicholas PJ. Day[2,6] MD[2], PhD, Arjen M. Dondorp MD, PhD[2,6], Prapit Teparrukkul MD[1], T. Eoin West MD, MPH[5,7], Direk Limmathurotsakul MD, PhD[2,6,8]

Short Title: Sepsis Fast Track Thailand

[1]Sunpasitthiprasong Hospital, Ubon Ratchathani, Thailand

[2]Mahidol Oxford Tropical Medicine Research Unit, Faculty of Tropical Medicine, Mahidol University, Bangkok, Thailand

[3]Department of Internal Medicine, Buddhachinaraj Phitsanulok Hospital, Phitsanulok, Thailand

[4]Department of Critical Care Medicine, University of Pittsburgh, Pittsburgh, Pennsylvania, USA

[5]Division of Pulmonary, Critical Care, and Sleep Medicine, University of Washington, Seattle, United States

[6]Centre for Tropical Medicine and Global Health, Nuffield Department of Medicine, University of Oxford, Churchill Hospital, Oxford, United Kingdom

[7]Department of Microbiology and Immunology, Faculty of Tropical Medicine, Mahidol University, Bangkok, Thailand

[8]Department of Tropical Hygiene, Faculty of Tropical Medicine, Mahidol University, Bangkok, Thailand

For peer review only - http://bmjopen.bmj.com/site/about/guidelines.xhtml

Supplementary Table 1 | Criteria used to systematically enroll patents into Sepsis Fast Track (SFT) upon admission

1. **Present with 2 or more of below Signs of systemic inflammatory response syndrome (SIRS)**
    - Body temperature > 38.3 °C or < 36.0 °C
    - Heart rate > 90 bpm
    - Respiratory rate > 20 pm or $PaCO_2$ < 32 mmHg
    - WBC > 12,000 /µL or < 4,000 /µL or Band forms > 10%
2. **Suspected sources of infection**
    - Pneumonia
    - Urinary track infection
    - Intra-abdominal infection
    - Skin and soft tissue infection
    - CNS infection
    - Others infections or unspecified source of infection
3. **Diagnostic criteria for severe sepsis:** patient met criteria in no 1 and 2 and has at least one of the following criteria
    - Mottled skin
    - Capillary refilling time ≥ 3 seconds
    - Urine output < 0.5 ml/kg/hour
    - Abrupt change in mental status
    - Acute respiratory failure
    - Platelet count < 100,000 /µL
    - Disseminated intravascular coagulation
    - Lactate > 2 mmol/L
    - SBP < 90 mmHg or MAP < 65 mmHg
4. **Diagnostic criteria for septic shock:** patient who are severe sepsis and has at least 1 of the following criteria
    - SBP < 90 mmHg or MAP < 65 mmHg after crystalliod administration ≥ 40-60 ml/kg of body weight OR after colloid administration ≥ 20-30 ml/kg of body weight
    - Require administration of dopamine > 5µg/kg of BW/min or norepinephrine / epinephrine > 0.02 µg/kg of BW/min to maintain MAP to be > 65 mmHg

For peer review only - http://bmjopen.bmj.com/site/about/guidelines.xhtml

Supplementary Table 2 | Systemic manifestation of infection criteria used for enrollment in Ubon-Sepsis Cohort

General parameters

1. Fever or hypothermia (Core body temperature defined as > 38.3 °C or < 36.0 °C)
2. Tachycardia (heart rate > 90 beats per minute)
3. Tachypnea (respiratory rate > 20 per minute)
4. Altered mental status with Glasgow Coma Score (GCS) < 15 or <10 if intubated
5. Hyperglycemia (plasma glucose > 140 mg/dL) in the absence of diabetes

Inflammatory parameters

6. Leukocytosis (white blood cell count > 12,000/µL), leukopenia (white blood cell count < 4000/µL) or immature forms > 10%
7. Plasma C-reactive protein > 2 SD above the normal value
8. Plasma procalcitonin > 2 SD above the normal value

Hemodynamic parameters

9. Arterial hypotension (systolic blood pressure (SBP) < 90 mmHg, mean arterial pressure (MAP) < 70 mmHg, or SBP decrease > 40 mmHg)

Organ dysfunction parameters

10. Low oxygen saturation determined by pulse oximetry (SpO2 < 95%) determined by pulse oximetry
11. Arterial hypoxemia (PaO2 / FIO2 < 300)
12. Acute oliguria (urine output < 0.5 mL/kg/hr or 45 mmol/L for 2 hours)
13. Creatinine increase > 0.5 mg/dL
14. Coagulation abnormalities (international normalised ratio >1·5 or activated partial thromboplastin time >60 seconds)
15. Thrombocytopenia (Platelet count < 100,000 cells/µL)
16. Ileus (absent bowel sounds)
17. Hyperbilirubinaemia (plasma total bilirubin > 4 mg/dL)

Tissue perfusion parameters

18. Hyperlactatemia (> 1 mmol/L)
19. Decreased capillary refill or mottling
20. Significant edema or positive fluid balance

For peer review only

For peer review only - http://bmjopen.bmj.com/site/about/guidelines.xhtml

89 **Supplementary Table 3| Factors associated with 28-day mortality using multivariable Cox**
90 **proportional hazards model without a variable of the direct admission to the ICU**

Variables	Died (n=779)	Survived (n=3027)	Adjusted hazard ratio (95%CI)	P value
**SFT group**[1]	205 (26%)	698 (23%)	0.77 (0.63- 0.94)	0.01
**Male gender**	445 (57%)	698 (23%)	0.86 (0.74- 1.00)	0.05
**Age group (years) (n [%])**				
• 18-40	59 (8%)	688 (23%)	1	<0.001
• >40-60	222 (29%)	930 (31%)	1.69 (1.26- 2.26)	
• >60-70	159 (20%)	568 (19%)	2.07 (1.52- 2.81)	
• >70	339 (44%)	841 (28%)	3.32 (2.50- 4.41)	
**Transferred from other hospital**	715 (92%)	2595 (86%)	1.16 (0.89- 1.52)	0.26
**Modified SOFA score (median, IQR)**	6 (4-9)	4 (3-6)	1.25 (1.22- 1.27)	<0.001
**Comorbidities**				
• Diabetes mellitus	205 (26%)	602 (20%)	1.08 (0.91- 1.27)	0.39
• Chronic kidney disease	141 (18%)	379 (13%)	1.20 (0.99- 1.45)	0.07
• Liver disease	39 (5%)	85 (3%)	1.24 (0.89- 1.72)	0.20
• Malignancy	24 (3%)	36 (1%)	2.52 (1.67- 3.81)	<0.001
**Blood culture positive for pathogenic organisms**	190 (24%)	332 (11%)	1.83 (1.54- 2.16)	<0.001
**Year**				
• 2013	165 (21%)	637 (21%)	1	0.34
• 2014	183 (23%)	651 (22%)	0.98 (0.79- 1.21)	
• 2015	207 (27%)	808 (27%)	1.01 (0.81- 1.26)	
• 2016[2]	224 (29%)	931 (31%)	0.85 (0.68- 1.07)	

91 [1] Enrolled in the Sepsis Fast Track (SFT) programme
92 [2] Included 28 patients in 2017

For peer review only - http://bmjopen.bmj.com/site/about/guidelines.xhtml

Supplementary Table 4 | Clinical management within the first day of hospital

Clinical management[1]	SFT group (n=903)	Control group (n=2903)	Adjusted odds ratio (95% CI)	P value
**Antibiotic**	897 (99%)	2497 (86%)	14.10 (6.10-32.60)	<0.001
**Blood culture**	829 (92%)	2387 (82%)	1.77 (1.31-2.38)	<0.001
**Urinary catherization**	862 (95%)	1642 (57%)	11.04 (7.71-15.80)	<0.001
**Acute dialysis**	10 (1.1%)	23 (0.8%)	2.08 (0.69-6.25)	0.19
**Adrenergic agent**	706 (78%)	902 (31%)	11.25 (8.86-14.28)	<0.001
**Mechanical ventilation**	290 (32%)	840 (29%)	0.30 (0.24-0.38)	<0.001

[1]The effect of SFT on each clinical management were estimated by using the multivariable logistic regression models adjusted for admission year, gender, age, comorbidities, modified SOFA score, transfer from other hospital, blood culture positive for pathogenic organisms, and direct admission to the ICU.

For peer review only

For peer review only - http://bmjopen.bmj.com/site/about/guidelines.xhtml

Supplementary Table 5 | Baseline characteristics of sepsis patients included in the Sepsis Fast Track (SFT) programme or standard of care (control) after the implementation of SFT programme. Values are number (percentages) unless stated otherwise

Characteristics	SFT group[2] (n=903)	Non-exposed group[3] (n=1267)	P value
**Male gender**	526 (58%)	720 (57%)	0.54
**Age (years) (median [IQR])**	63 (49-74)	59 (41-73)	<0.001
**Age group (years) (n [%])**			
18-40	100 (11%)	293 (23%)	<0.001
>40-60	277 (31%)	350 (28%)	
>60-70	214 (24%)	230 (18%)	
>70	312 (35%)	394 (31%)	
**Comorbidities**	486 (54%)	666 (53%)	0.56
Hypertension	239 (26%)	349 (28%)	0.58
Diabetes mellitus	213 (24%)	286 (23%)	0.58
Chronic kidney disease	129 (14%)	197 (16%)	0.42
Dyslipidemia	66 (7%)	80 (6%)	0.36
Heart disease	48 (5%)	98 (8%)	0.03
Lung disease	67 (7%)	126 (10%)	0.04
Liver disease	33 (4%)	46 (4%)	0.98
Cerebrovascular disease	29 (3%)	25 (2%)	0.07
Malignancy	13 (1%)	33 (3%)	0.06
Human immunodeficiency virus (HIV)	6 (1%)	9 (1%)	0.90
**Organ dysfunction[4]**			
Total modified SOFA score (median [IQR])	6 (4-9)	4 (3-6)	<0.001
Renal dysfunction[4]	706 (78%)	743 (59%)	<0.001
Cardiovascular dysfunction[4]	811 (90%)	546 (43%)	<0.001
Coagulation dysfunction[4]	419 (46%)	650 (51%)	0.03
Liver dysfunction[4]	311 (34%)	318 (25%)	<0.001
Respiratory dysfunction[4]	337 (37%)	400 (32%)	0.01
Central nervous system dysfunction[4]	166 (18%)	217 (17%)	0.61
**Transferred from other hospitals**	878 (97%)	1041 (82%)	<0.001
**Duration of symptoms (median [IQR])**	2 (1-3)	3 (1-5)	<0.001
≤ 2 days	505 (56%)	537 (42%)	<0.001
3-7 days	362 (40%)	650 (51%)	
> 7 days	36 (4%)	80 (6%)	
**Presenting clinical syndromes[5] (n [%])**			
Septic shock	687 (76%)	179 (14%)	<0.001

For peer review only - http://bmjopen.bmj.com/site/about/guidelines.xhtml

For peer review only

Characteristics	SFT group[2] (n=903)	Non-exposed group[3] (n=1267)	P value
Acute febrile illness	206 (23%)	485 (38%)	<0.001
Lower respiratory infection	223 (25%)	457 (36%)	<0.001
Sepsis	225 (25%)	137 (11%)	<0.001
Others	13 (1%)	168 (13%)	<0.001
Diarrheal illness	150 (17%)	105 (8%)	<0.001
**Admitted directly to an ICU upon admission**	170 (19%)	39 (3%)	<0.001
**Blood culture positive for pathogenic organisms**	175 (19%)	160 (13%)	<0.001
**Year**			
2015	356 (39%)	659 (52%)	<0.001
2016	535 (59%)	592 (47%)	
2017	12 (1%)	16 (1%)	

[1]Organ dysfunction is defined by modified SOFA ≥2
[2]Sepsis patients who were identified and treated in Sepsis-FT system in 2015 – January 2017.
[3]Sepsis patients whom were not in Sepsis-FT system.
[4]Organ dysfunction defined as modified SOFA score was ≥1 for each organ system.
[5]Patients may have more than one presenting clinical syndrome.

For peer review only

For peer review only - http://bmjopen.bmj.com/site/about/guidelines.xhtml

Supplementary Table 6 | Factors associated with 28-day mortality using multivariable Cox proportional hazards model in 2,170 patients enrolled into the study after the implementation of the Sepsis Fast Track (SFT) programme

Variables	Died (n=431)	Survived (n=1739)	Adjusted hazard ratio (95%CI)	P value
**SFT group**	205(48%)	698(40%)	0.68 (0.55- 0.84)	<0.001
**Male gender**	254(59%)	992(57%)	0.89 (0.73- 1.09)	0.26
**Age group (years) (n [%])**				
• 18-40	30(7%)	363(21%)	1	<0.001
• >40-60	125(29%)	502(29%)	1.97 (1.31- 2.95)	
• >60-70	85(20%)	359(21%)	1.97 (1.28- 3.03)	
• >70	191(44%)	515(30%)	3.33 (2.24- 4.95)	
**Transferred from other hospital**	406(94%)	1513(87%)	1.49 (0.99- 2.26)	0.06
**Modified SOFA score (median, IQR)**	7 (5-10)	4 (3-6)	1.24 (1.21- 1.28)	<0.001
**Comorbidities**				
• Diabetes mellitus	122(28%)	377(22%)	1.08 (0.87- 1.35)	0.49
• Chronic kidney disease	84(19%)	242(14%)	1.23 (0.96- 1.58)	0.11
• Liver disease	22(5%)	57(3%)	1.10 (0.71- 1.70)	0.68
• Malignancy	19(4%)	27(2%)	2.90 (1.81- 4.63)	<0.001
**Blood culture positive for pathogenic organisms**	110(26%)	225(13%)	1.64 (1.31- 2.05)	<0.001
**Year**				
• 2015	207(48%)	808(46%)	1	0.08
• 2016	224(52%)	931(54%)	0.84 (0.69- 1.02)	
**Direct admission to the ICU**	85(20%)	124(7%)	1.78 (1.37- 2.32)	<0.001

For peer review only

For peer review only - http://bmjopen.bmj.com/site/about/guidelines.xhtml

Supplementary Figure 1 | Preprinted recommended doctor orders for sepsis fast track programme used at the Emergency Department at Sunpasitthiprasong Hopsital from 1 January 2015

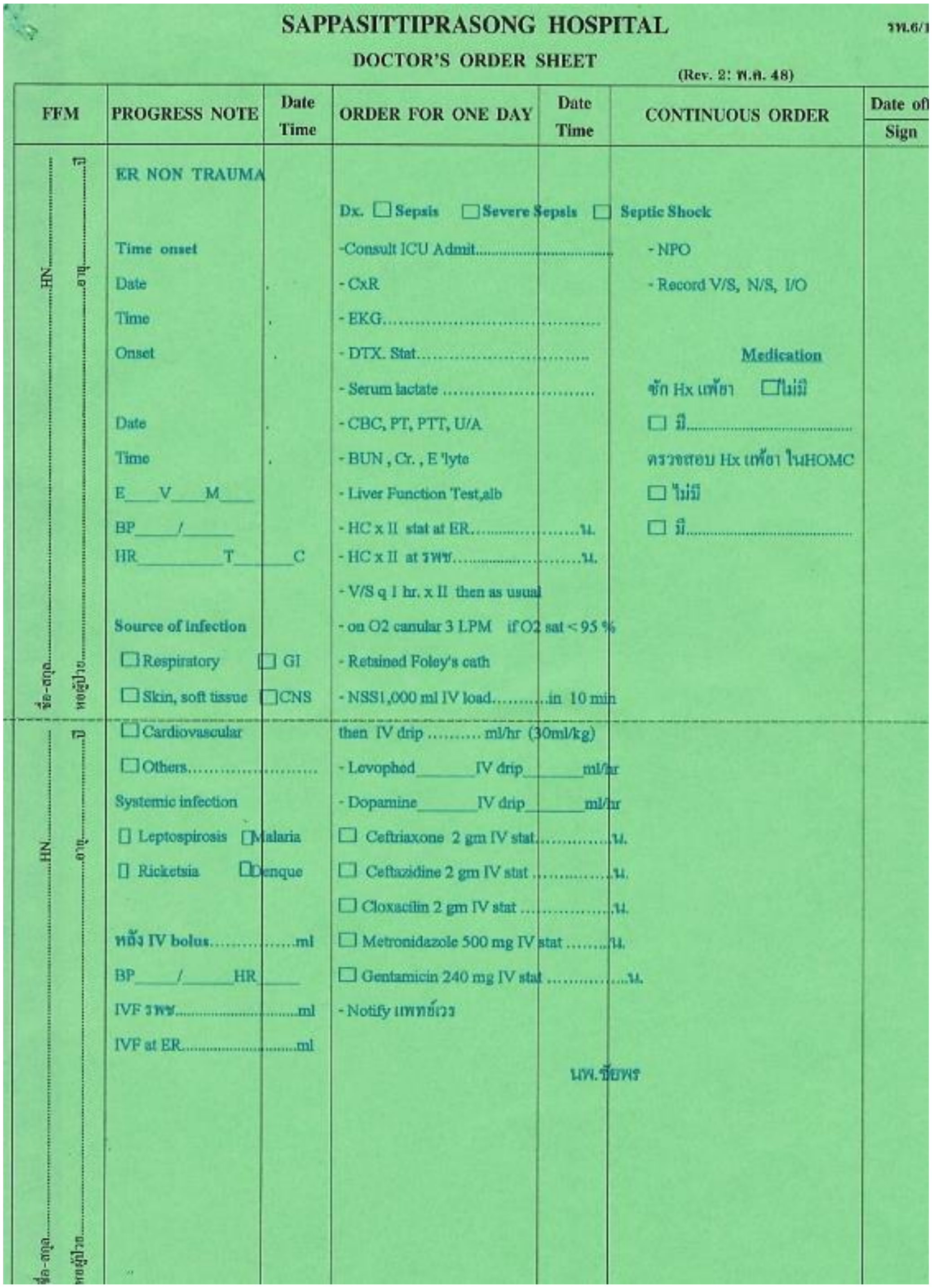

SAPPASITTIPRASONG HOSPITAL

รพ.6/1

DOCTOR'S ORDER SHEET

(Rev. 2: พ.ศ. 48)

FFM	PROGRESS NOTE	Date Time	ORDER FOR ONE DAY	Date Time	CONTINUOUS ORDER	Date of Sign
HN. ชื่อ-สกุล อายุ ปี หอผู้ป่วย	ER NON TRAUMA<br>Time onset<br>Date .<br>Time .<br>Onset .<br>Date .<br>Time .<br>E___V___M___<br>BP____/_____<br>HR_______T_____C<br>Source of infection<br>☐ Respiratory ☐ GI<br>☐ Skin, soft tissue ☐ CNS<br>☐ Cardiovascular<br>☐ Others……………………<br>Systemic infection<br>☐ Leptospirosis ☐ Malaria<br>☐ Ricketsia ☐ Denque<br>หลัง IV bolus……………ml<br>BP____/_____HR_____<br>IVF รพช.……………………ml<br>IVF at ER……………………ml		Dx. ☐ Sepsis ☐ Severe Sepsis ☐ Septic Shock<br>-Consult ICU Admit……………………<br>- CxR<br>- EKG……………………………<br>- DTX. Stat……………………<br>- Serum lactate ………………………<br>- CBC, PT, PTT, U/A<br>- BUN , Cr. , E 'lyte<br>- Liver Function Test,alb<br>- HC x II stat at ER………………น.<br>- HC x II at รพช.………………น.<br>- V/S q 1 hr. x II then as usual<br>- on O2 canular 3 LPM if O2 sat < 95 %<br>- Retained Foley's cath<br>- NSS1,000 ml IV load………in 10 min<br>then IV drip ………. ml/hr (30ml/kg)<br>- Levophed______IV drip______ml/hr<br>- Dopamine______IV drip______ml/hr<br>☐ Ceftriaxone 2 gm IV stat…………น.<br>☐ Ceftazidine 2 gm IV stat …………น.<br>☐ Cloxacilin 2 gm IV stat ……………น.<br>☐ Metronidazole 500 mg IV stat ………น.<br>☐ Gentamicin 240 mg IV stat ……………น.<br>- Notify แพทย์เวร<br>นพ.ชัยพร		- NPO<br>- Record V/S, N/S, I/O<br>**Medication**<br>ซัก Hx แพ้ยา ☐ไม่มี<br>☐ มี……………………………<br>ตรวจสอบ Hx แพ้ยา ในHOMC<br>☐ ไม่มี<br>☐ มี……………………………	

Supplementary Figure 2 | The Sepsis Fast Track sepsis resuscitation workflow used at the Emergency Department at Sunpasitthiprasong Hopsital from 1 March 2016

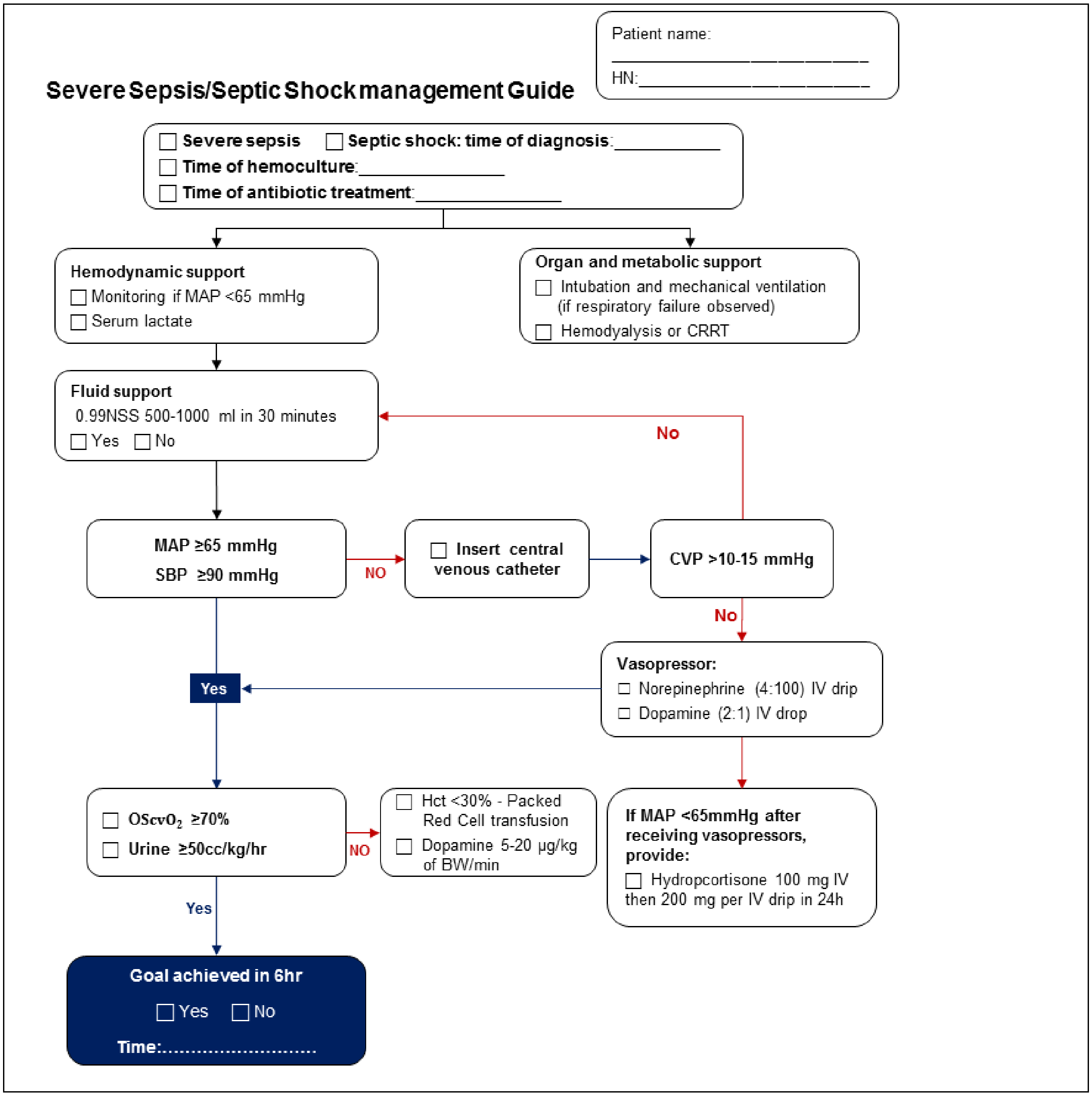

STROBE Statement

Checklist of items that should be included in reports of observational studies

Section/Topic	Item No	Recommendation	Reported on Page No
**Title and abstract**	1	(*a*) Indicate the study's design with a commonly used term in the title or the abstract	1, 3
		(*b*) Provide in the abstract an informative and balanced summary of what was done and what was found	3-4
**Introduction**			
Background/rationale	2	Explain the scientific background and rationale for the investigation being reported	5
Objectives	3	State specific objectives, including any prespecified hypotheses	6
**Methods**			
Study design	4	Present key elements of study design early in the paper	6
Setting	5	Describe the setting, locations, and relevant dates, including periods of recruitment, exposure, follow-up, and data collection	6
Participants	6	(*a*) *Cohort study*—Give the eligibility criteria, and the sources and methods of selection of participants. Describe methods of follow-up<br>*Case-control study*—Give the eligibility criteria, and the sources and methods of case ascertainment and control selection. Give the rationale for the choice of cases and controls<br>*Cross-sectional study*—Give the eligibility criteria, and the sources and methods of selection of participants	7-8
		(*b*) *Cohort study*—For matched studies, give matching criteria and number of exposed and unexposed<br>*Case-control study*—For matched studies, give matching criteria and the number of controls per case	
Variables	7	Clearly define all outcomes, exposures, predictors, potential confounders, and effect modifiers. Give diagnostic criteria, if applicable	8-9
Data sources/measurement	8*	For each variable of interest, give sources of data and details of methods of assessment (measurement). Describe comparability of assessment methods if there is more than one group	8-9
Bias	9	Describe any efforts to address potential sources of bias	11-12
Study size	10	Explain how the study size was arrived at	10
Quantitative variables	11	Explain how quantitative variables were handled in the analyses. If applicable, describe which groupings were chosen and why	11-12
Statistical methods	12	(*a*) Describe all statistical methods, including those used to control for confounding	11-12
		(*b*) Describe any methods used to examine subgroups and interactions	11-12
		(*c*) Explain how missing data were addressed	8
		(*d*) *Cohort study*—If applicable, explain how loss to follow-up was addressed<br>*Case-control study*—If applicable, explain how matching of cases and controls was addressed<br>*Cross-sectional study*—If applicable, describe analytical methods taking account of sampling strategy	6
		(*e*) Describe any sensitivity analyses	12

For peer review only - http://bmjopen.bmj.com/site/about/guidelines.xhtml

Section/Topic	Item No	Recommendation	Reported on Page No
**Results**			
Participants	13*	(a) Report numbers of individuals at each stage of study—eg numbers potentially eligible, examined for eligibility, confirmed eligible, included in the study, completing follow-up, and analysed	13
		(b) Give reasons for non-participation at each stage	
		(c) Consider use of a flow diagram	
Descriptive data	14*	(a) Give characteristics of study participants (eg demographic, clinical, social) and information on exposures and potential confounders	13-14
		(b) Indicate number of participants with missing data for each variable of interest	
		(c) *Cohort study*—Summarise follow-up time (eg, average and total amount)	
Outcome data	15*	*Cohort study*—Report numbers of outcome events or summary measures over time	15-16
		Case-control study—Report numbers in each exposure category, or summary measures of exposure	
		Cross-sectional study—Report numbers of outcome events or summary measures	
Main results	16	(*a*) Give unadjusted estimates and, if applicable, confounder-adjusted estimates and their precision (eg, 95% confidence interval). Make clear which confounders were adjusted for and why they were included	14-16
		(*b*) Report category boundaries when continuous variables were categorized	
		(*c*) If relevant, consider translating estimates of relative risk into absolute risk for a meaningful time period	
Other analyses	17	Report other analyses done—eg analyses of subgroups and interactions, and sensitivity analyses	17-19
**Discussion**			
Key results	18	Summarise key results with reference to study objectives	19-21
Limitations	19	Discuss limitations of the study, taking into account sources of potential bias or imprecision. Discuss both direction and magnitude of any potential bias	22
Interpretation	20	Give a cautious overall interpretation of results considering objectives, limitations, multiplicity of analyses, results from similar studies, and other relevant evidence	21-23
Generalisability	21	Discuss the generalisability (external validity) of the study results	22
**Other Information**			
Funding	22	Give the source of funding and the role of the funders for the present study and, if applicable, for the original study on which the present article is based	24

**Give information separately for cases and controls in case-control studies and, if applicable, for exposed and unexposed groups in cohort and cross-sectional studies.*

Note: An Explanation and Elaboration article discusses each checklist item and gives methodological background and published examples of transparent reporting. The STROBE checklist is best used in conjunction with this article (freely available on the Web sites of PLoS Medicine at http://www.plosmedicine.org/, Annals of Internal Medicine at http://www.annals.org/, and Epidemiology at http://www.epidem.com/). Information on the STROBE Initiative is available at www.strobe-statement.org.

For peer review only - http://bmjopen.bmj.com/site/about/guidelines.xhtml

For peer review only

BMJ Open

Effectiveness of a sepsis programme in a resource-limited setting: a retrospective analysis of data of a prospective observational study (Ubon-sepsis)

Journal:	*BMJ Open*
Manuscript ID	bmjopen-2020-041022.R2
Article Type:	Original research
Date Submitted by the Author:	18-Dec-2020

Complete List of Authors:	Booraphun, Suchart; Sunpasithiprasong Hospital, Medical Department<br>Hantrakun, Viriya; Mahidol Oxford Tropical Medicine Research Unit, Microbiology<br>Siriboon, Suwatthiya; Sunpasithiprasong Hospital<br>Boonsri, Chaiyaporn ; Sunpasithiprasong Hospital, Emergency Department<br>Poomthong, Pulyamon; Sunpasithiprasong Hospital<br>Singkaew, Bung-Orn; Sunpasithiprasong Hospital<br>Wasombat, Oratai; Sunpasithiprasong Hospital<br>Chamnan, Parinya; Sunpasithiprasong Hospital<br>Champunot, Ratapum; Buddhachinaraj Phitsanulok Hospital, Department of Internal Medicine<br>Rudd, Kristina; University of Pittsburgh, Department of Critical Care Medicine<br>Day, Nicholas; Mahidol Oxford Tropical Medicine Research Unit; Univerity of Oxford Nuffield Department of Medicine, Centre for Tropical Medicine<br>Dondorp, Arjen; Mahidol Oxford Tropical Medicine Research Unit; Univerity of Oxford Nuffield Department of Medicine, Centre for Tropical Medicine<br>Teparrukkul, Prapit; Sunpasithiprasong Hospital<br>West, Timothy Eoin; University of Washington, Division of Pulmonary, Critical Care, and Sleep Medicine; Mahidol University Faculty of Tropical Medicine, Department of Microbiology and Immunology<br>Limmathurotsakul, Direk; Mahidol Oxford Tropical Medicine Research Unit; Mahidol University Faculty of Tropical Medicine, Department of Tropical Hygiene

**Primary Subject Heading**:	Intensive care
Secondary Subject Heading:	Infectious diseases, Epidemiology
Keywords:	Epidemiology < INFECTIOUS DISEASES, INFECTIOUS DISEASES, INTENSIVE & CRITICAL CARE

I, the Submitting Author has the right to grant and does grant on behalf of all authors of the Work (as defined in the below author licence), an exclusive licence and/or a non-exclusive licence for contributions from authors who are: i) UK Crown employees; ii) where BMJ has agreed a CC-BY licence shall apply, and/or iii) in accordance with the terms applicable for US Federal Government officers or employees acting as part of their official duties; on a worldwide, perpetual, irrevocable, royalty-free basis to BMJ Publishing Group Ltd ("BMJ") its licensees and where the relevant Journal is co-owned by BMJ to the co-owners of the Journal, to publish the Work in this journal and any other BMJ products and to exploit all rights, as set out in our licence.

The Submitting Author accepts and understands that any supply made under these terms is made by BMJ to the Submitting Author unless you are acting as an employee on behalf of your employer or a postgraduate student of an affiliated institution which is paying any applicable article publishing charge ("APC") for Open Access articles. Where the Submitting Author wishes to make the Work available on an Open Access basis (and intends to pay the relevant APC), the terms of reuse of such Open Access shall be governed by a Creative Commons licence – details of these licences and which Creative Commons licence will apply to this Work are set out in our licence referred to above.

Other than as permitted in any relevant BMJ Author's Self Archiving Policies, I confirm this Work has not been accepted for publication elsewhere, is not being considered for publication elsewhere and does not duplicate material already published. I confirm all authors consent to publication of this Work and authorise the granting of this licence.

For peer review only

Effectiveness of a sepsis programme in a resource-limited setting: a retrospective analysis of data of a prospective observational study (Ubon-sepsis)

Suchart Booraphun MD[1], Viriya Hantrakun PhD[2], Suwatthiya Siriboon MD[1], Chaiyaporn Boonsri MD[1], Pulyamon Poomthong MD[1], Bung-Orn Singkaew BNS[1], Oratai Wasombat BNS[1], Parinya Chamnan MD, PhD[1], Ratapum Champunot MD[3], Kristina Rudd MD, MPH[4, 5], Nicholas PJ. Day[2,6] MD[2], PhD, Arjen M. Dondorp MD, PhD[2,6], Prapit Teparrukkul MD[1], T. Eoin West MD, MPH[5,7], Direk Limmathurotsakul MD, PhD[2,6,8]

Short Title: Sepsis Fast Track Thailand

[1]Sunpasitthiprasong Hospital, Ubon Ratchathani, Thailand

[2]Mahidol Oxford Tropical Medicine Research Unit, Faculty of Tropical Medicine, Mahidol University, Bangkok, Thailand

[3]Department of Internal Medicine, Buddhachinaraj Phitsanulok Hospital, Phitsanulok, Thailand

[4]Department of Critical Care Medicine, University of Pittsburgh, Pittsburgh, Pennsylvania, USA

[5]Division of Pulmonary, Critical Care, and Sleep Medicine, University of Washington, Seattle, United States

[6]Centre for Tropical Medicine and Global Health, Nuffield Department of Medicine, University of Oxford, Churchill Hospital, Oxford, United Kingdom

[7]Department of Microbiology and Immunology, Faculty of Tropical Medicine, Mahidol University, Bangkok, Thailand

For peer review only - http://bmjopen.bmj.com/site/about/guidelines.xhtml

[8]Department of Tropical Hygiene, Faculty of Tropical Medicine, Mahidol University, Bangkok, Thailand

***Corresponding author:** Assoc. Prof. Direk Limmathurotsakul, Mahidol-Oxford Tropical Medicines Research Unit, Faculty of Tropical Medicine, Mahidol University, 420/6 Rajvithi Road, Bangkok, 10400, Thailand. Tel: +66 2 203 6333, E-mail: direk@tropmedres.ac (DL)

Word count: abstract 285, text 3,829

Number of pages: 32, Tables: 2; Figures: 1, Supporting Information: 1

Keywords: sepsis, sepsis care, sepsis management, mortality, resource limited setting

For peer review only

Abstract

Objective: To evaluate the effectiveness of a Sepsis Fast Track (SFT) programme initiated at a regional referral hospital in Thailand in January 2015

Design: A retrospective analysis using the data of a prospective observational study (Ubon-sepsis) from March 2013 to January 2017

Setting: General medical wards and medical intensive care units (ICUs) of a study hospital

Participants: Patients with community-acquired sepsis observed under the Ubon-sepsis cohort. Sepsis was defined as modified Sequential Organ Failure Assessment (SOFA) score ≥ 2.

Main exposure: The SFT programme was a protocol to identify and initiate sepsis care on hospital admission, implemented at the study hospital in 2015. Patients in the SFT programme were admitted directly to the ICUs when available. The non-exposed group comprised of patients who received standard of care.

Main outcome: The primary outcome was 28-day mortality. The secondary outcomes were measured sepsis management interventions.

Results: Of 3,806 sepsis patients, 903 (24%) were detected and enrolled in the SFT programme of the study hospital (SFT group) and 2,903 received standard of care (non-exposed group). Patients in the SFT group had more organ dysfunction, were more likely to receive measured sepsis management and to be admitted directly to the ICU (19% vs. 4%). Patients in the SFT group were more likely to survive (adjusted hazard ratio 0.72; 95% CI 0.58 to 0.88, p=0.001) adjusted for admission year, gender, age, comorbidities, modified SOFA score and direct admission to the ICUs.

For peer review only - http://bmjopen.bmj.com/site/about/guidelines.xhtml

Conclusions: The SFT programme is associated with improved sepsis care and lower risk of death in sepsis patients in rural Thailand, where some critical care resources are limited. The survival benefit is observed even when all patients enrolled in the programme could not be admitted directly into the ICUs.

Study registration number: NCT02217592

Strengths and limitations of this study

- The study hospital utilized the published framework, SCAN-TEACH-TREAT programme to develop a context specific quality of care improvement for sepsis in a tropical resource-limited setting.
- The study took advantage of a robust prospective observational study design that strengthened causal inference by providing pre-intervention information, having an appropriate control group from both pre and post-intervention periods, and controlling important confounding factors (i.e. the modified SOFA score).
- We found that most measured sepsis interventions increased.
- The study did not record dosage of dobutamine, dopamine, epinephrine and norepinephrine, arterial blood gases were rarely performed, and the modified SOFA score (maximum 23) may be lower than the SOFA score (maximum 24).
- The observational study may have residual confounding factors such as improvement of care and profile of organ failure recognition overtimes.

For peer review only

For peer review only - http://bmjopen.bmj.com/site/about/guidelines.xhtml

INTRODUCTION

Sepsis is defined as life-threatening organ dysfunction caused by a dysregulated host response to infection,[1] and is the primary cause of death from infection, especially if not recognized and treated promptly.[2-4] Sepsis is a major cause of health loss worldwide and is associated with approximately eleven million deaths each year, most of which occur in low and middle-income countries (LMICs).[5] The United Nations World Health Assembly has recognized sepsis as a global health priority and adopted a resolution on improving its worldwide prevention, diagnosis and management.[6] Comprehensive guidelines such as those developed by the Surviving Sepsis Campaign have been associated with reduced mortality in high-income countries,[2-4] but effectiveness of these guidelines in LMICs needs more evaluation.[7-10]

Following the Surviving Sepsis Campaign (SSC) 2012,[11] the Ministry of Public Health Thailand and the Thai Society of Critical Care Medicine developed local recommendations on sepsis based on resource availability and local context.[12] The recommendations suggest that secondary-care and tertiary-care hospitals in the country should develop a Sepsis Fast Track (SFT) so that, on presentation, sepsis patients can be identified, treated and directly admitted to the ICUs when available. One small retrospective study showed lower mortality among sepsis patients enrolled than those not enrolled in the SFT (21% vs. 43%) at the study hospital,[13] while another study did not find an association between SFT and mortality outcome.[14] These studies were subject to selection biases due to their retrospective nature.[13-14] Interventional studies to randomize patients to receive or not receive the SFT, however, would be unethical and impractical after the national recommendations have been implemented. It is increasingly recommended to evaluate the impact

For peer review only - http://bmjopen.bmj.com/site/about/guidelines.xhtml

of healthcare interventions using routine data, particularly when a wide range of routinely collected data is available.[15]

Here, we analysed data from our prospective observational study of community-acquired sepsis patients presenting to a referral hospital in Thailand over four years (from March 2013 to January 2017)[16-17] to retrospectively evaluate the effectiveness of a SFT programme which was implemented at the study hospital in January 2015.

MATERIAL AND METHODS

Study design

We conducted a retrospective study to evaluate the effectiveness of the SFT programme by using the data of a prospective observational study (Ubon-sepsis).[16-17] The SFT programme was implemented at the study hospital in January 2015 until now as per national recommendations.[12] The SFT programme at the study hospital included (1) diagnostic criteria for attending physicians and medical teams to systematically identify sepsis patients on hospital admission (Supplementary Table 1), (2) a recommended sepsis care protocol and (3) direct admission to the ICUs when available. The SFT programme at the study hospital was generated by the SFT committee of the study hospital (S.B., S.S., C.B., P.P., B.S., O.W., P.C. and P.T.) based on SSC 2012,[11] resource availability and local context.[12] The study hospital is a referral hospital to smaller district hospitals and provincial hospitals in three adjacent provinces. The referring hospitals were not involved in the SFT programme of the study hospital during the study period.

For peer review only - http://bmjopen.bmj.com/site/about/guidelines.xhtml

Details of the Ubon-sepsis cohort have been published elsewhere.[16-17] In short, the Ubon-sepsis research team, who were not attending physicians or medical teams at the study hospital, conducted a prospective observational study of community-acquired infections and sepsis from March 2013 to January 2017.[16-17] The research team prospectively enrolled adult patients ≥ 18 years old who were admitted to the general medical wards and medical intensive care units (ICUs) with a primary diagnosis of infection made by the attending physician, were within 24 hours of admission to the study hospital, and had three of 20 systemic manifestations of infection documented in the medical records (Supplementary Table 2). The 20 systemic manifestations of the infections were consolidated from the 22 variables proposed as diagnostic criteria for sepsis for SSC 2012.[11] The study team sequentially screened all medical patients by reviewing admission logs in the emergency department (ED), medical wards, and medical ICUs twice daily (morning and afternoon) on each working day. The Ubon-sepsis cohort was initiated in 2012 prior to the implementation of SFT at the study hospital. The research team was not involved in any clinical interventions; enrollment in the SFT programme and all medical treatment was performed by attending physicians and medical teams. The research team did not adjust the study protocol, inclusion criteria and exclusion criteria of the Ubon-sepsis cohort during the entire study period, and the research team recorded whether participants in the Ubon-sepsis cohort were enrolled in the SFT programme.

The reporting of this study follows the STROBE guidelines. Written, informed permission was obtained from participants prior to enrollment in the Ubon-sepsis cohort.

For peer review only - http://bmjopen.bmj.com/site/about/guidelines.xhtml

Participants

For this study, we evaluated patients who were included into the Ubon-sepsis cohort and had community-acquired sepsis. Sepsis was defined as an infection with organ dysfunction in accordance with the 2016 international Consensus (Sepsis-3) guidelines for sepsis.[1] Organ dysfunction was determined by a modified sequential (sepsis-based) organ failure assessment (SOFA) score ≥2 as previously described.[16-17] The study was conducted in 2013 prior to the Sepsis-3 definition, and inotropic and vasopressor agent doses were not recorded into the CRF.[1, 18] For the cardiovascular component of the SOFA score, the scoring was modified such that subjects were scored a maximum of 2 (on a 4-point scale) if they received only dobutamine or dopamine, and scored a maximum of 3 if they received epinephrine or norepinephrine. For the respiratory component of the SOFA score, as PaO2/FiO2 indices were not available for the majority of subjects due to infrequency of arterial blood gas tests, the score was modified as follows: Subjects were scored a maximum of 2 (4-point scale) if they received advanced respiratory support (endotracheal tube, gas powered or electrical powered mechanical ventilation) and arterial blood gas test was not performed.[16-17] The Ubon-sepsis cohort excluded patients who were suspected of having hospital-acquired infections (determined by the attending physician), hospitalized within 30 days prior to the current admission, or hospitalized at any facility for a total duration longer than 72 hours prior to enrollment.

Main exposure

Main exposure of the study was the SFT programme. All patients included in the Ubon-sepsis cohort from March 2013 to December 2014 who received standard care were considered as the

For peer review only

For peer review only - http://bmjopen.bmj.com/site/about/guidelines.xhtml

non-exposed group. Patients included in the Ubon-sepsis cohort from January 2015 to January 2017 who received standard care or received care in the SFT programme by attending medical teams using their criteria on admission (Supplementary Table 1) were considered as the additional non-exposed group or as the SFT group, respectively. The Ubon-sepsis research team were not involved in decision-making regarding enrollment to the SFT programme.

Patients in the non-exposed group received standard care according to local guidelines. Patients in the SFT group received the standard of care along with a recommended sepsis care protocol of the SFT programme. First, preprinted recommended doctor orders for the SFT programme were used as of January 2015 (Supplementary Figure 1). The recommended orders included oxygen administration, intravenous fluid loading and fluid administration to achieve the recommended target of 30 mL/kg crystalloid, blood culture, recommended stat (immediate) doses and choices of parenteral antibiotics including ceftriaxone, ceftazidime, cloxacillin, metronidazole and gentamycin, contact ICU for ICU admission (if available), oxygen supplementation, close monitoring of vital signs and urine output, and a set of diagnostic tests including chest radiography, electrocardiogram, rapid blood glucose test, serum lactate, complete blood count, blood urea nitrogen, creatinine, electrolytes, liver function tests, albumin level, prothrombin time and partial thromboplastin time. Second, as of March 2016, the resuscitation workflow to normalize and maintain a mean arterial pressure (MAP) ≥65 mmHg, systolic blood pressure (SBP) ≥90 mmHg and urine output ≥0.5 mL/kg/hr within the first six hours was formally implemented and recommended (Supplementary Figure 2). The resuscitation workflow included fluid resuscitation, measurement of central venous pressure (CVP) and central venous oxygen saturation ($SCVO_2$),

For peer review only - http://bmjopen.bmj.com/site/about/guidelines.xhtml

administration of adrenergic agents, blood transfusion for haematocrit <30% and hydrocortisone if adequate fluid resuscitation and vasopressor therapy could not restore hemodynamic stability. The resuscitation workflow was pre-printed and included in the clinical chart of every SFT patient (together with pre-printed doctor's orders), and was recommended even if patients could not be admitted directly to the ICU. A separate set of documents, recommended management and recommended frequency of vital signs monitoring for nurses (i.e. nurse notes for SFT patients) were also used for every SFT patient. Preparation and regular meetings to implement and monitor the SFT programme were organized by the SFT committee of Sunpasitthiprasong Hospital.

Outcome measures

The primary outcome measure was 28-day mortality as recorded in the Ubon-sepsis cohort.[16] 28-day mortality data were collected via telephone contact if subjects were no longer hospitalized and had been discharged alive.[16] The secondary outcome measures were sepsis management interventions; including antibiotics administration, blood cultures, mechanical ventilation, adrenergic agents, acute haemodialysis and placement of a urinary catheter within the first day of hospitalization.[16-17]

Sample size

The sample size of the study was determined by the sample size of Ubon-sepsis cohort. We assumed that about 50% of 3,806 sepsis patients in the Ubon-sepsis cohort were enrolled after the implementation of the SFT programme, of which 50% were enrolled in the SFT programme (i.e. 952 and 2,854 patients were estimated to be the SFT and non-exposed group, respectively). We

For peer review only - http://bmjopen.bmj.com/site/about/guidelines.xhtml

207 assumed that the mortality of the non-exposed group was 21% based on published data.[16-17] Our current sample size of 3,806 would provide a power of 80% at an alpha error of 5% to detect a 4% difference in the mortality outcome.

Statistical analysis

All sepsis patients were included in the analysis regardless of whether they were enrolled before or after the implementation of the SFT programme. We used the Chi-square test and Mann-Whitney test to compare the proportions of binary variables and median of continuous variables between groups, respectively. The interquartile range is presented as 25th and 75th percentiles.

In the primary analysis, we used multivariable Cox proportional hazard models to evaluate the effectiveness of SFT programme on 28-day mortality. The multivariable Cox proportional hazard model was used to adjust the difference between those receiving the SFT programme and the others.[19] To reduce bias in the model development, we used the previous multivariable Cox proportional hazard model as the base model,[16] added the SFT group variable and direct admission to the ICU, and modified by adding a time variable to represent possible changes over time and by using continuous modified SOFA score on admission rather than as a binary variable (modified SOFA score ≥ 2). Twenty eight patients enrolled in early 2017 were considered as enrolled in 2016. The continuous modified SOFA score was used to improve regression adjustment for disease severity of the model. The other variables included in the model were gender, age group, transfer from other hospital, comorbidities (diabetes mellitus, chronic kidney disease, liver disease and malignancy) and blood culture positive for pathogenic organisms. We calculated the unadjusted

For peer review only - http://bmjopen.bmj.com/site/about/guidelines.xhtml

and adjusted probability of survival at each timepoint using the Kaplan-Meier method (using the sts graph and stcurve command in STATA, respectively)

Using a conceptual framework, we also consider that admission directly to the ICU could also be a mediator between the SFT and the primary outcome; therefore, we developed another multivariable model not including the variable for direct admission to the ICU. The goodness of fit for the multivariable Cox proportional hazard model was tested with a Hosmer and Lemeshow test. For the Cox proportional hazard model, we assessed whether the hazard ratio was constant over time using Schoenfeld residuals.

For the secondary endpoints, we used multivariable logistic regression models with similar independent variables as the model for 28-mortality outcome and used each sepsis management process as an outcome. We estimated the total effect of the SFT on each sepsis management by using the multivariable logistic regression models adjusted for difference in characteristics and disease severity of the patients. This was because each sepsis management could be caused by characteristics of the patients, disease severity and the SFT.[20]

We also performed sensitivity analyses by using multivariable logistic regression model, excluding patients enrolled prior to the implementation of the SFT programme, and by replacing direct admission to the ICUs with admission to the ICUs within the first hospital day. All analyses were performed with STATA 15.1 (StataCorp, College Station, TX, USA).

For peer review only - http://bmjopen.bmj.com/site/about/guidelines.xhtml

Patient and public involvement

No patients were involved in setting the research question or the outcome measures, nor were they involved in developing plans for recruitment, design, or implementation of the study. No patients were asked to advice on interpretation or writing the results. The results will be disseminated to the public through online social media.

RESULTS

Baseline characteristics

The observational cohort study (Ubon-sepsis) included 5,001 patients presenting with community-acquired infections from March 2013 to January 2017, and 12 patients were excluded due to unknown 28-day mortality outcome. 3,806 (76%) met criteria for sepsis within the first 24 hours of admission with a modified SOFA score ≥2, and were included for the analysis. Figure 1 shows the flow of participants through the study. Among 3,806 sepsis patients, 903 were enrolled in the SFT programme and considered as the SFT group, and 2,903 were not enrolled in the SFT programme, received standard of care, and considered as the non-exposed group. Of 2,903 sepsis patients in the non-exposed group, 1,636 were included in the observational cohort study prior to the implementation of SFT programme and 1,267 were after the implementation of the programme.

Table 1 shows the characteristics of the study patients. Patients in the SFT group were older and more likely to have underlying diseases of diabetes mellitus, cerebrovascular diseases and dyslipidemia. Patients included in the SFT group had higher severity of organ dysfunction determined by the modified SOFA score compared with the non-exposed group (median 6 [IQR

For peer review only

For peer review only - http://bmjopen.bmj.com/site/about/guidelines.xhtml

4-9] vs. 4 [IQR 3-6], p<0.001). A higher proportion of patients in the SFT group were admitted directly to the ICU compared with the non-exposed group (19% vs 5%, p<0.001).

Table 1 | Baseline characteristics of sepsis patients enrolled in the Sepsis Fast Track programme[1] (SFT group) or standard of care (non-exposed group). Values are number (percentages) unless stated otherwise

**Characteristics**	**SFT group[2] (n=903)**	**Non-exposed group[3] (n=2903)**
**Male gender**	526 (58%)	1653 (57%)
**Age (years) (median [IQR])**	63 (49-74)	56 (39-70)
**Age group (years)**		
18-40	100 (11%)	647 (22%)
>40-60	277 (31%)	875 (30%)
>60-70	214 (24%)	513 (18%)
>70	312 (35%)	868 (30%)
**Comorbidities**		
Hypertension	239 (26%)	726 (25%)
Diabetes mellitus	213 (24%)	594 (20%)
Chronic kidney disease	129 (14%)	391 (13%)
Dyslipidemia	66 (7%)	152 (5%)
Heart disease	48 (5%)	183 (6%)
Lung disease	67 (7%)	239 (8%)
Liver disease	33 (4%)	91 (3%)
Cerebrovascular disease	29 (3%)	55 (2%)
Malignancy	13 (1%)	47 (2%)
Human immunodeficiency virus (HIV)	6 (1%)	33 (1%)
**Organ dysfunction**		
Modified SOFA score (median [IQR])	6 (4-9)	4 (3-6)
Renal dysfunction[4]	706 (78%)	1846 (64%)
Cardiovascular dysfunction[4]	811 (90%)	1532 (53%)
Coagulation dysfunction[4]	419 (46%)	1562 (54%)
Liver dysfunction[4]	311 (34%)	822 (28%)
Respiratory dysfunction[4]	337 (37%)	853 (29%)
Central nervous system dysfunction[4]	166 (18%)	530 (18%)

For peer review only

For peer review only - http://bmjopen.bmj.com/site/about/guidelines.xhtml

Characteristics	SFT group[2] (n=903)	Non-exposed group[3] (n=2903)
**Transferred from other hospitals**	874 (97%)	2372 (84%)
**Duration of symptoms (median [IQR])**	2 (1-3)	3 (1-5)
≤ 2 days	505 (56%)	1191 (41%)
3-7 days	362 (40%)	1488 (51%)
> 7 days	36 (4%)	224 (8%)
**Presenting clinical syndromes[5]**		
Septic shock	687 (76%)	733 (25%)
Acute febrile illness	206 (23%)	940 (32%)
Lower respiratory infection	223 (25%)	890 (31%)
Sepsis	225 (25%)	273 (9%)
Others	13 (1%)	456 (16%)
Diarrheal illness	150 (17%)	264 (9%)
**Direct admission to the ICU**	170 (19%)	128 (4%)
**Admission to the ICU within 24 hours of admission**	270 (29%)	370 (13%)
**Blood culture positive for pathogenic organisms**	175 (19%)	347 (12%)
**Year**		
2013	N/A	1047 (26%)
2014	N/A	1156 (29%)
2015	369 (39%)	956 (24%)
2016	556 (59%)	869 (21%)
2017	14 (1%)	22 (1%)

[1] SFT programme was implemented at the study hospital in January 2015.

[2] 903 patients of the Ubon-sepsis cohort were enrolled in SFT programme after the implementation of the SFT programme (Figure 1)

[3] Included 1,636 and 1,267 patients in the Ubon-sepsis cohort before and after the implementation of the SFT programme, respectively.

[4] Organ dysfunction defined as modified SOFA score was ≥1 for each organ system.[16]

[5] Patients may have more than one presenting clinical syndrome.

For peer review only - http://bmjopen.bmj.com/site/about/guidelines.xhtml

For peer review only

Primary outcomes

The primary outcome, mortality within 28 days, occurred in 205 of 903 (23%) in the SFT group and 574 of 2,903 (20%) in the non-exposed group (Figure 2A). In the primary analysis, patients in the SFT group were more likely to survive adjusted for baseline characteristics, severity of sepsis and direct admission the ICUs (adjusted hazard ratio [aHR] 0.72, 95% CI 0.58-0.88, p=0.001, Figure 2B and Supplementary Table 3). Older age, higher modified SOFA score, underlying disease of malignancy and chronic kidney disease, blood culture positive for pathogenic organisms and direct admission to the ICUs were associated with risk of mortality.

Sensitivity Analyses

As we considered that direct admission to the ICU could be a mediator between the SFT and the outcome, a sensitivity analysis was performed by excluding the variable direct admission to the ICU (Supplementary Table 4). The effect of SFT (aHR 0.77, 95% CI 0.63-0.94, p<0.001) was also observed. We observed that constant proportional hazard assumption was not strongly hold in one variable (the modified SOFA score); therefore, additional sensitivity analyses were performed by using logistic multivariable models. The similar effect of SFT was observed (Supplementary Table 5 and 6).

We also performed a sensitivity analysis by excluding the 1,636 patients enrolled in the observational study prior to the implementation of SFT programme. Similar differences in baseline characteristics were observed when comparing 903 patients in the SFT group to the 1,267 patients in the non-exposed group enrolled after the implementation of the SFT programme

For peer review only - http://bmjopen.bmj.com/site/about/guidelines.xhtml

(Supplementary Table 7). A higher chance of survival in the SFT group compared to the non-exposed group was also observed (aHR 0.68, 95% CI 0.55 to 0.84, p<0.001; Supplementary Table 7). We also performed another sensitivity analysis by replacing direct admission to the ICUs with admission to the ICUs within the first hospital day. Of 3,806 patients, 640 (17%) were admitted to the ICUs within the first day of admission. A higher chance of survival in the SFT group compared to the non-exposed group was also observed (aHR 0.72, 95% CI 0.59 to 0.88, p=0.002).

Secondary outcomes

Using multivariable logistic regression models, we found that patients in the SFT group were more likely to receive most sepsis management interventions than patients in the non-exposed group adjusting for baseline characteristics, severity of sepsis and direct admission to the ICU (Table 2). Those included antibiotics, blood cultures, adrenergic agents, and placement of a urinary catheter within the first day of hospitalization. However, sepsis patients in the SFT group were less likely to receive mechanical ventilation compared with those in the non-exposed group adjusting for baseline characteristics, severity of sepsis and direct admission to the ICUs group (adjusted odds ratio [aOR] 0.30; 95% CI 0.24 to 0.38). We found that direct admission to the ICUs (aOR 5.77, 95% CI 4.20 to 7.92) and transfer from other hospitals (aOR 3.45, 95% CI 2.42 to 4.91) were strongly associated with the requirement of mechanical ventilation.

Table 2 | Clinical management within the first day of hospital

For peer review only - http://bmjopen.bmj.com/site/about/guidelines.xhtml

Clinical management[1]	SFT group (n=903)	Control group (n=2903)	Adjusted odds ratio (95% CI)	P value
**Antibiotic**	897 (99%)	2497 (86%)	14.69 (6.36-33.91)	<0.001
**Blood culture**	829 (92%)	2387 (82%)	1.82 (1.35-2.45)	<0.001
**Urinary catherization**	862 (95%)	1642 (57%)	12.02 (8.41-17.20)	<0.001
**Acute dialysis**	10 (1.1%)	23 (0.8%)	1.96 (0.66-5.87)	0.23
**Adrenergic agent**	706 (78%)	902 (31%)	11.53 (9.10-14.61)	<0.001
**Mechanical ventilation**	290 (32%)	840 (29%)	0.39 (0.31-0.49)	<0.001
**Direct admission to the ICU**	170 (18.8%)	128 (4.4%)	4.34 (2.96-6.36)	<0.001

[1]The effect of SFT on each clinical management were estimated by using the multivariable logistic regression models adjusted for admission year, gender, age, comorbidities, modified SOFA score, transfer from other hospital, and blood culture positive for pathogenic organisms, and direct admission to the ICU.

DISCUSSION

In this study evaluating patients with community-acquired sepsis, enrollment into a programme to identify and initiate sepsis care implemented at the study hospital (SFT programme) was associated with 28% lower risk of mortality. In recent years, there has been an increasing need to understand benefit and cost effectiveness of implementation of sepsis care interventions in LMICs because of concerns that international sepsis guidelines[11] may not be extrapolated to patients with tropical infectious diseases[7-9] and to resource-limited settings with poor ICU capacity.[10] In this study we show the effectiveness of sepsis protocol modified based on resource availability in a tropical country, where causes of community-acquired sepsis include malaria and tropical viral diseases.[16, 21-22] Majority of sepsis patients in our study were managed on the general wards, including those with respiratory failure or shock. Nonetheless, our study shows that enhancing sepsis care in the

For peer review only - http://bmjopen.bmj.com/site/about/guidelines.xhtml

emergency department and general medical wards, as well as improving access to ICUs can reduce sepsis mortality in a LMIC.

The lower odds of receiving mechanical ventilation in the SFT group could be a sign of improved sepsis care. Patients in the SFT group are monitored closely either in or outside the ICUs, and the attending physicians aim to obviate the need for airway intubation when possible.[7] Attending physicians may tend to provide mechanical ventilation to patients in the non-exposed group based on broad indications such as (1) airway protection, (2) hypercapnic respiratory failure, (3) hypoxemic respiratory failure or (4) circulatory failure[23-24] because they may not be able to monitor patients' breathing and oxygen saturation as often as those enrolled in the SFT programme.

It is not surprising that patients in the SFT group had more organ dysfunction than those in the non-exposed group. This is because the severity of organ dysfunction among patients with septic shock, respiratory failure and alteration of conscious can be assessed clinically on admission, and those patients could be enrolled in the SFT programme when the laboratory test results were not yet available. However, the non-exposed group were defined as having sepsis based on clinical findings and all laboratory test results within 24 hours of admission (per protocol of Ubon-sepsis cohort study[16-17]). Therefore, the non-exposed group could use laboratory test results (i.e. liver function tests, creatinine level, international normalised ratio and activated partial thromplastin time) from blood specimens drawn on admission. Therefore, the SFT programme were more likely to enroll patients with obvious signs of sepsis and septic shock; such as acute respiratory failure

For peer review only

For peer review only - http://bmjopen.bmj.com/site/about/guidelines.xhtml

and hypotension, while Ubon-sepsis cohort could include sepsis patients with relatively lower modified SOFA scores.

Comparison with other studies

Our study is not the first to evaluate effectiveness of sepsis intervention in LMICs. Early recognition and protocol directed intervention improves outcomes of sepsis in adults[25-27] and severe infection in children[28] in LMICs. The optimal method of fluid resuscitation in sepsis in tropical LMICs has not been determined.[8, 25, 29-30] Our resuscitation protocol is a simple guideline, and the SFT recommend doctors to be careful and adjust fluid resuscitation based on preliminary diagnoses, underlying diseases and rapid diagnostic test results (i.e. if sepsis is caused by malaria or dengue infection). The implementation of the SFT programme in our study hospital and in Thailand is consistent with the recommendation of "SCAN-TEACH-TREAT" programme developed by Sepsis in Resource-Limited Settings Workgroup of the Surviving Sepsis Campaign.[7] The SFT programme evaluated resources in the setting (SCAN component), focused on educational interventions on early recognition and management of sepsis among medical personnel including physicians, nurses and students (TEACH component) and implemented pragmatic and simple bundles into practice (TREAT component). In addition, the SFT programme has the strong support and endorsement of local health and governmental leaders.[12]

Strength and limitations of the study

This study features four strengths. First, the study hospital utilized the published framework, SCAN-TEACH-TREAT programme to develop a context specific quality of care improvement for

For peer review only - http://bmjopen.bmj.com/site/about/guidelines.xhtml

sepsis,[7] and we closely monitor and evaluate the effectiveness of an intervention. Second, the study took advantage of a robust prospective observational study design that strengthened causal inference by providing pre-intervention information, having an appropriate non-exposed group from both pre and post-intervention periods, and controlling important confounding factors (i.e. the modified SOFA score) which were measured systematically throughout the study period. Third, this study incorporated several predictors of interest (measured sepsis management interventions and admission to the ICUs). This allows us to identify that the increase in most measured sepsis interventions associated with the SFT programme and that led to the survival benefit among sepsis patients. Fourth, the focus on sepsis at a public tertiary-care hospital in Thailand helped us to estimate the effect of sepsis protocol in a tropical resource-limited setting with large sample size.

Our study had several limitations. First, a modified SOFA score was used because the dosage of dobutamine, dopamine, epinephrine and norepinephrine were not recorded and arterial blood gases were rarely performed. The modified SOFA score (maximum 23) may be lower than the SOFA score (maximum 24). Nonetheless, the modified SOFA score is strongly associated with mortality in sepsis.[16-17] Second, the proportional hazards assumption was met for all variables, including the main variable (the SFT), except one controlled variable (the modified SOFA score). The adjusted effect estimates could be under or overestimated due to residual confounding factors such as improvement of care and profile of organ failure recognition overtimes. Third, due to the use of observational data, the observed effects of the SFT on 28-day mortality in our study should be interpreted conservatively as an association rather than a causation.

For peer review only

For peer review only - http://bmjopen.bmj.com/site/about/guidelines.xhtml

Conclusions and future implications

Our study successfully demonstrated effectiveness of a sepsis programme implemented in a LMIC. Measuring effectiveness of a sepsis programme is a complex issue, and we utilized a data of a prospective observational study and carefully controlled for severity of sepsis and temporal trends in our analyses. Care in sepsis patients improved after the implementation of the programme. Additional research is needed to better understand cost of the intervention, long-term benefits and impact of the programme on a national scale. National strategies aimed at saving lives from sepsis in LMICs should be encouraged. Such strategies should include analysis of resources and local circumstances, followed by development, implementation and assessment of customized programmes.

Acknowledgement:

We thank all patients, their relatives, and staff of the Sunpasitthiprasong hospital who participated in the study. We thank Mayura Malasit, Praweennuch Watanachaiprasert, Chayamon Krainoonsing, Passaraporn Kesaphun, Nannicha Jirapornuwat, Gumphol Wongsuvan, Areeya Faosap, Yaowaret Dokket, Sukhumal Pewlaorng, Jintana Suwannapruek, Prapass Wannapinij and Diane Tomita for their clinical, laboratory and administrative support.

For peer review only - http://bmjopen.bmj.com/site/about/guidelines.xhtml

Contributors:

NPJD, TEW, and DL obtained grant funding. SB, VH, PT, TEW, and DL contributed to study conception development and study design. SB, VH, PT, TEW and DL contributed to study conduct, data collection, and study administration. VH and DL performed the statistical analysis and interpreted the data and had full access to all of the data in the study. Both authors can take responsibility for the integrity of the data and the accuracy of the data analysis. DL is a guarantor. SB, VH, PT, TEW, and DL wrote the first draft of a manuscript, with input from SS, CB, PC, KR, and AD. PP, BS, OW, and RC provided scientific or administrative support. All authors contributed to results interpretation, critically revised, and approved the final submitted manuscript. The corresponding author attests that all listed authors meet authorship criteria and that no others meeting the criteria have been omitted.

Funding:

The study was funded by the Wellcome Trust (090219/Z/09/Z) and National Heart, Lung and Blood Institute, National Institutes of Health (R01HL113382). DL is supported by an intermediate fellowship from the Wellcome Trust (101103/Z/13/Z). The funders had no role in the design and conduct of the study, all study procedures, data collection, data analyses, data interpretation, writing of the report, and the decision to submit the article for publication.

Competing interests:

The authors declare that they have no completing interests.

For peer review only - http://bmjopen.bmj.com/site/about/guidelines.xhtml

Ethics approvals:

The study was conducted the study in full compliance with the principles of good clinical practice (GCP), and the ethical principles of the Declaration of Helsinki. The study protocol and related documents were approved by Sunpasitthiprasong Hospital Ethics Committee (039/2556), the Ethics Committee of the Faculty of Tropical Medicine, Mahidol University (MUTM2012-024-01), the University of Washington Institutional Review Board (42988) and the Oxford Tropical Research Ethics Committee at the University of Oxford (OXTREC172-12). Signed or fingerprinted informed consent was obtained from the participants or their representatives before enrollment.

Data sharing:

The final database with the data dictionary are publicly available online https://doi.org/10.6084/m9.figshare.12102627.

The lead authors (SB, VH and DL) affirm that the manuscript is an honest, accurate, and transparent account of the study being reported; that no important aspects of the study have been omitted; and that any discrepancies from the study as planned have been explained. This is an Open Access article distributed in accordance with the terms of the Creative Commons Attribution (CC BY 4.0) license, which permits unrestricted reuse, distribution, and reproduction in any medium, provided the original work is properly cited. See: http://creativecommons.org/licenses/by/4.0/

For peer review only - http://bmjopen.bmj.com/site/about/guidelines.xhtml

Dissemination to participants and related patient and public communities:

The results of this study will be disseminated to physicians at the study hospital, health care providers, policy makers, and academic communities through various mediums, including printed report, internal hospital meetings, academic conferences, and institutional networks. The results from this study will be used to inform the current Sepsis Fast Track programme at Sunpasitthiprasong hospital and the community hospitals which are located in jurisdiction of the study hospital catchment areas in the Northeast Thailand. The study results will not be disseminated to patients or general population, because study results are in medical context.

For peer review only

For peer review only - http://bmjopen.bmj.com/site/about/guidelines.xhtml

References

1 Singer M, Deutschman CS, Seymour CW, et al. The Third International Consensus Definitions for Sepsis and Septic Shock (Sepsis-3). *JAMA* 2016;315(8):801-10. doi: 10.1001/jama.2016.0287

2 Rhodes A, Evans LE, Alhazzani W, et al. Surviving Sepsis Campaign: International Guidelines for Management of Sepsis and Septic Shock 2016. *Crit Care Med* 2017;45(3):486-552. doi: 10.1097/ccm.0000000000002255

3 WHO. WHO Sepsis Technical Expert Meeting - Meeting report Geneva: World Health Organization; 2018 [Available from: https://www.who.int/servicedeliverysafety/areas/sepsis_meeting-report-2018.pdf accessed 25 November 2019 2019].

4 Vincent J-L. The Clinical Challenge of Sepsis Identification and Monitoring. *PLoS Med* 2016;13(5):e1002022. doi: 10.1371/journal.pmed.1002022

5 Rudd KE, Johnson SC, Agesa KM, et al. Global, regional, and national sepsis incidence and mortality, 1990–2017: analysis for the Global Burden of Disease Study. *The Lancet* 2020;395(10219):200-11. doi: 10.1016/S0140-6736(19)32989-7

6 Reinhart K, Daniels R, Kissoon N, et al. Recognizing Sepsis as a Global Health Priority — A WHO Resolution. *N Engl J Med* 2017;377(5):414-17. doi: 10.1056/NEJMp1707170

For peer review only - http://bmjopen.bmj.com/site/about/guidelines.xhtml

7 Kwizera A, Baelani I, Mer M, et al. The long sepsis journey in low- and middle-income countries begins with a first step...but on which road? *Crit Care* 2018;22(1):64. doi: 10.1186/s13054-018-1987-z

8 McGloughlin S, Richards GA, Nor MBM, et al. Sepsis in tropical regions: Report from the task force on tropical diseases by the World Federation of Societies of Intensive and Critical Care Medicine. *J Crit Care* 2018;46:115-18. doi: 10.1016/j.jcrc.2017.12.018

9 Becker JU, Theodosis C, Jacob ST, et al. Surviving sepsis in low-income and middle-income countries: new directions for care and research. *Lancet Infect Dis* 2009;9(9):577-82. doi: 10.1016/S1473-3099(09)70135-5

10 Schultz MJ, Dunser MW, Dondorp AM, et al. Current challenges in the management of sepsis in ICUs in resource-poor settings and suggestions for the future. *Intensive Care Med* 2017;43(5):612-24. doi: 10.1007/s00134-017-4750-z

11 Dellinger RP, Levy MM, Rhodes A, et al. Surviving Sepsis Campaign: international guidelines for management of severe sepsis and septic shock, 2012. *Intensive Care Med* 2013;39(2):165-228. doi: 10.1007/s00134-012-2769-8

12 Office of permanent secretatry, Ministry of Public Health, Thailand. The Development of Health Service Plan and Indicators of Health Outcomes 2018:8.

13 Ruangchan S, Chusri S, Saengsanga P, et al. Clinical Outcomes of Community-Acquired Severe Sepsis after Implementation of a Simple Severe Sepsis Fast Track. *J Med Assoc Thai* 2016;99(8):877-85.

For peer review only - http://bmjopen.bmj.com/site/about/guidelines.xhtml

14 Ittisanyakorn M, Ruchichanantakul S, Vanichkulbodee A, et al. Prevalence and factors associated with one-year mortality of infectious diseases among elderly emergency department patients in a middle-income country. *BMC Infect Dis* 2019;19(1):662. doi: 10.1186/s12879-019-4301-z

15 Clarke GM, Conti S, Wolters AT, et al. Evaluating the impact of healthcare interventions using routine data. *BMJ* 2019;365:l2239. doi: 10.1136/bmj.l2239

16 Hantrakun V, Somayaji R, Teparrukkul P, et al. Clinical epidemiology and outcomes of community acquired infection and sepsis among hospitalized patients in a resource limited setting in Northeast Thailand: A prospective observational study (Ubon-sepsis). *PLoS One* 2018;13(9):e0204509. doi: 10.1371/journal.pone.0204509

17 Rudd KE, Hantrakun V, Somayaji R, et al. Early management of sepsis in medical patients in rural Thailand: a single-center prospective observational study. *J Intensive Care* 2019;7:55. doi: 10.1186/s40560-019-0407-z

18 Seymour CW, Liu VX, Iwashyna TJ, et al. Assessment of Clinical Criteria for Sepsis: For the Third International Consensus Definitions for Sepsis and Septic Shock (Sepsis-3). *Jama* 2016;315(8):762

19 Craig P, Cooper C, Gunnell D, et al. Using natural experiments to evaluate population health interventions: new Medical Research Council guidance. *J Epidemiol Community Health* 2012;66(12):1182-6. doi: 10.1136/jech-2011-200375

20 Westreich D, Greenland S. The Table 2 Fallacy: Presenting and Interpreting Confounder and Modifier Coefficients. *Am J Epidemiol* 2013;177(4):292-98. doi: 10.1093/aje/kws412

For peer review only - http://bmjopen.bmj.com/site/about/guidelines.xhtml

21 Teparrukkul P, Hantrakun V, Imwong M, et al. Utility of qSOFA and modified SOFA in severe malaria presenting as sepsis. *PLoS One* 2019;14(10):e0223457. doi: 10.1371/journal.pone.0223457

22 Teparrukkul P, Hantrakun V, Day NPJ, et al. Management and outcomes of severe dengue patients presenting with sepsis in a tropical country. *PLoS One* 2017;12(4):e0176233. doi: 10.1371/journal.pone.0176233

23 Pierson DJ. Indications for mechanical ventilation in adults with acute respiratory failure. *Respir Care* 2002;47(3):249-62; discussion 62-5.

24 Pham T, Brochard LJ, Slutsky AS. Mechanical Ventilation: State of the Art. *Mayo Clin Proc* 2017;92(9):1382-400. doi: 10.1016/j.mayocp.2017.05.004

25 Jacob ST, Banura P, Baeten JM, et al. The impact of early monitored management on survival in hospitalized adult Ugandan patients with severe sepsis: a prospective intervention study*. *Crit Care Med* 2012;40(7):2050-8. doi: 10.1097/CCM.0b013e31824e65d7

26 Machado FR, Ferreira EM, Schippers P, et al. Implementation of sepsis bundles in public hospitals in Brazil: a prospective study with heterogeneous results. *Crit Care* 2017;21(1):268. doi: 10.1186/s13054-017-1858-z

27 Noritomi DT, Ranzani OT, Monteiro MB, et al. Implementation of a multifaceted sepsis education program in an emerging country setting: clinical outcomes and cost-effectiveness in a long-term follow-up study. *Intensive Care Med* 2014;40(2):182-91. doi: 10.1007/s00134-013-3131-5

For peer review only

For peer review only - http://bmjopen.bmj.com/site/about/guidelines.xhtml

28 Sazawal S, Black RE, Pneumonia Case Management Trials G. Effect of pneumonia case management on mortality in neonates, infants, and preschool children: a meta-analysis of community-based trials. *Lancet Infect Dis* 2003;3(9):547-56. doi: 10.1016/s1473-3099(03)00737-0

29 Maitland K, Kiguli S, Opoka RO, et al. Mortality after fluid bolus in African children with severe infection. *N Engl J Med* 2011;364(26):2483-95. doi: 10.1056/NEJMoa1101549

30 Andrews B, Semler MW, Muchemwa L, et al. Effect of an Early Resuscitation Protocol on In-hospital Mortality Among Adults With Sepsis and Hypotension: A Randomized Clinical Trial. *JAMA* 2017;318(13):1233-40. doi: 10.1001/jama.2017.10913

For peer review only - http://bmjopen.bmj.com/site/about/guidelines.xhtml

579 **Figure legends**

580 **Figure 1| Flow of participants through study**

581 **Footnote of figure 1:** This study used the data of an observational study on sepsis patients (Ubon-sepsis) from March 2013 to January 2017 to evaluate the effectiveness of a Sepsis Fast Track (SFT) programme implemented at the study hospital in January 2015

585 **Figure 2| (A) Unadjusted probability of survival and (B) Adjusted probability of survival based on the multivariable Cox proportional hazard regression model**

For peer review only

For peer review only - http://bmjopen.bmj.com/site/about/guidelines.xhtml

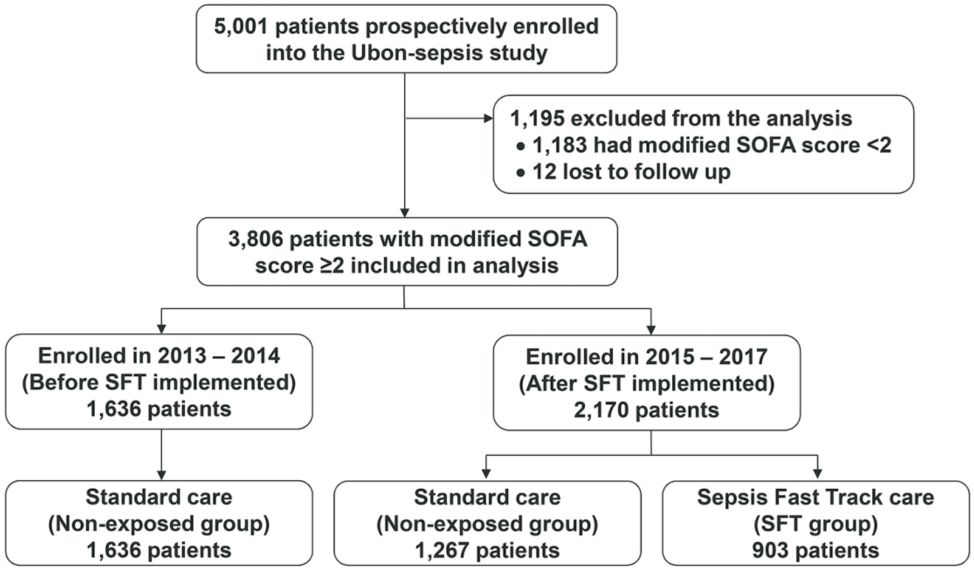

Figure 1 Flow of participants through study

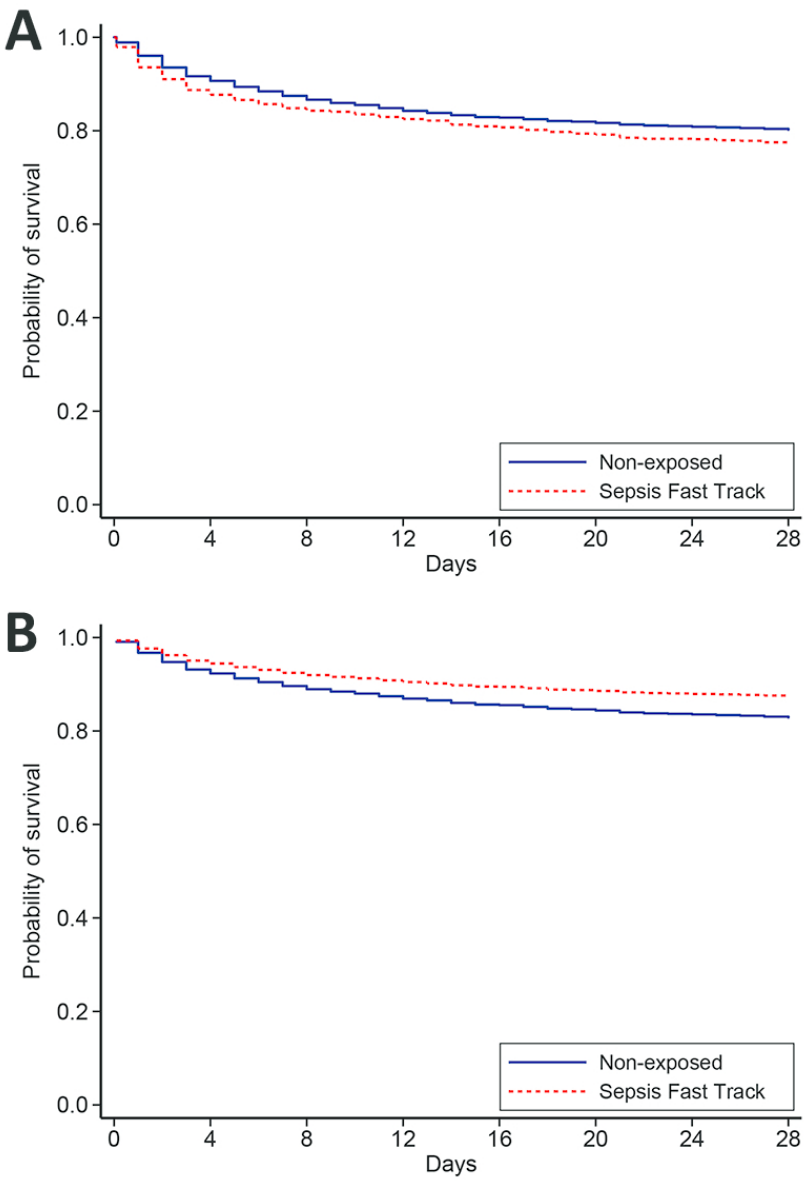

Figure 2 (A) Unadjusted probability of survival and (B) Adjusted probability of survival based on the multivariable Cox proportional hazard regression model

Effectiveness of a sepsis programme in a resource-limited setting: a retrospective analysis of data of a prospective observational study (Ubon-sepsis)

Suchart Booraphun MD[1], Viriya Hantrakun PhD[2], Suwatthiya Siriboon MD[1], Chaiyaporn Boonsri MD[1], Pulyamon Poomthong MD[1], Bung-Orn Singkaew BNS[1], Oratai Wasombat BNS[1], Parinya Chamnan MD, PhD[1], Ratapum Champunot MD[3], Kristina Rudd MD, MPH[4, 5], Nicholas PJ. Day[2,6] MD[2], PhD, Arjen M. Dondorp MD, PhD[2,6], Prapit Teparrukkul MD[1], T. Eoin West MD, MPH[5,7], Direk Limmathurotsakul MD, PhD[2,6,8]

Short Title: Sepsis Fast Track Thailand

[1]Sunpasitthiprasong Hospital, Ubon Ratchathani, Thailand

[2]Mahidol Oxford Tropical Medicine Research Unit, Faculty of Tropical Medicine, Mahidol University, Bangkok, Thailand

[3]Department of Internal Medicine, Buddhachinaraj Phitsanulok Hospital, Phitsanulok, Thailand

[4]Department of Critical Care Medicine, University of Pittsburgh, Pittsburgh, Pennsylvania, USA

[5]Division of Pulmonary, Critical Care, and Sleep Medicine, University of Washington, Seattle, United States

[6]Centre for Tropical Medicine and Global Health, Nuffield Department of Medicine, University of Oxford, Churchill Hospital, Oxford, United Kingdom

[7]Department of Microbiology and Immunology, Faculty of Tropical Medicine, Mahidol University, Bangkok, Thailand

[8]Department of Tropical Hygiene, Faculty of Tropical Medicine, Mahidol University, Bangkok, Thailand

For peer review only

For peer review only - http://bmjopen.bmj.com/site/about/guidelines.xhtml

Supplementary Table 1 | Criteria used to systematically enroll patents into Sepsis Fast Track (SFT) upon admission

1. **Present with 2 or more of below Signs of systemic inflammatory response syndrome (SIRS)**
    - Body temperature > 38.3 °C or < 36.0 °C
    - Heart rate > 90 bpm
    - Respiratory rate > 20 pm or $PaCO_2$ < 32 mmHg
    - WBC > 12,000 /µL or < 4,000 /µL or Band forms > 10%
2. **Suspected sources of infection**
    - Pneumonia
    - Urinary track infection
    - Intra-abdominal infection
    - Skin and soft tissue infection
    - CNS infection
    - Others infections or unspecified source of infection
3. **Diagnostic criteria for severe sepsis:** patient met criteria in no 1 and 2 and has at least one of the following criteria
    - Mottled skin
    - Capillary refilling time ≥ 3 seconds
    - Urine output < 0.5 ml/kg/hour
    - Abrupt change in mental status
    - Acute respiratory failure
    - Platelet count < 100,000 /µL
    - Disseminated intravascular coagulation
    - Lactate > 2 mmol/L
    - SBP < 90 mmHg or MAP < 65 mmHg
4. **Diagnostic criteria for septic shock:** patient who are severe sepsis and has at least 1 of the following criteria
    - SBP < 90 mmHg or MAP < 65 mmHg after crystalliod administration ≥ 40-60 ml/kg of body weight OR after colloid administration ≥ 20-30 ml/kg of body weight
    - Require administration of dopamine > 5µg/kg of BW/min or norepinephrine/ epinephrine > 0.02 µg/kg of BW/min to maintain MAP to be > 65 mmHg

For peer review only - http://bmjopen.bmj.com/site/about/guidelines.xhtml

Supplementary Table 2 | Systemic manifestation of infection criteria used for enrollment in Ubon-Sepsis Cohort

General parameters

1. Fever or hypothermia (Core body temperature defined as > 38.3 °C or < 36.0 °C)
2. Tachycardia (heart rate > 90 beats per minute)
3. Tachypnea (respiratory rate > 20 per minute)
4. Altered mental status with Glasgow Coma Score (GCS) < 15 or <10 if intubated
5. Hyperglycemia (plasma glucose > 140 mg/dL) in the absence of diabetes

Inflammatory parameters

6. Leukocytosis (white blood cell count > 12,000/µL), leukopenia (white blood cell count < 4000/µL) or immature forms > 10%
7. Plasma C-reactive protein > 2 SD above the normal value
8. Plasma procalcitonin > 2 SD above the normal value

Hemodynamic parameters

9. Arterial hypotension (systolic blood pressure (SBP) < 90 mmHg, mean arterial pressure (MAP) < 70 mmHg, or SBP decrease > 40 mmHg)

Organ dysfunction parameters

10. Low oxygen saturation determined by pulse oximetry (SpO2 < 95%) determined by pulse oximetry
11. Arterial hypoxemia (PaO2 / FIO2 < 300)
12. Acute oliguria (urine output < 0.5 mL/kg/hr or 45 mmol/L for 2 hours)
13. Creatinine increase > 0.5 mg/dL
14. Coagulation abnormalities (international normalised ratio >1·5 or activated partial thromboplastin time >60 seconds)
15. Thrombocytopenia (Platelet count < 100,000 cells/µL)
16. Ileus (absent bowel sounds)
17. Hyperbilirubinaemia (plasma total bilirubin > 4 mg/dL)

Tissue perfusion parameters

18. Hyperlactatemia (> 1 mmol/L)
19. Decreased capillary refill or mottling
20. Significant edema or positive fluid balance

For peer review only

Supplementary Table 3 | Factors associated with 28-day mortality using multivariable Cox proportional hazards model

Variables	Died (n=779)	Survived (n=3027)	Adjusted hazard ratio (95%CI)	P value
**SFT group**[1]	205 (26%)	698 (23%)	0.72 (0.58-0.88)	0.001
**Male gender**	445 (57%)	698 (23%)	0.87 (0.75-1.01)	0.06
**Age group (years) (n [%])**				
• 18-40	59 (8%)	688 (23%)	1	<0.001
• >40-60	222 (29%)	930 (31%)	1.72 (1.28-2.30)	
• >60-70	159 (20%)	568 (19%)	2.10 (1.54-2.86)	
• >70	339 (44%)	841 (28%)	3.41 (2.57-4.53)	
**Transferred from other hospital**	715 (92%)	2595 (86%)	1.14 (0.88-1.49)	0.33
**Modified SOFA score (median, IQR)**	6 (4-9)	4 (3-6)	1.23 (1.21-1.26)	<0.001
**Comorbidities**				
• Diabetes mellitus	205 (26%)	602 (20%)	1.06 (0.90-1.26)	0.47
• Chronic kidney disease	141 (18%)	379 (13%)	1.22 (1.01-1.48)	0.04
• Liver disease	39 (5%)	85 (3%)	1.27 (0.91-1.76)	0.16
• Malignancy	24 (3%)	36 (1%)	2.64 (1.75-3.99)	<0.001
**Blood culture positive for pathogenic organisms**	190 (24%)	332 (11%)	1.83 (1.55-2.17)	<0.001
**Year**				
• 2013	165 (21%)	637 (21%)	1	0.30
• 2014	183 (23%)	651 (22%)	1.03 (0.83-1.27)	
• 2015	207 (27%)	808 (27%)	1.05 (0.84-1.31)	
• 2016[2]	224 (29%)	931 (31%)	0.88 (0.70-1.11)	
**Direct admission to the ICU**	128 (16%)	170 (6%)	1.68 (1.36-2.06)	<0.001

[1] Enrolled in the Sepsis Fast Track (SFT) programme
[2] Included 28 patients in 2017

For peer review only

For peer review only - http://bmjopen.bmj.com/site/about/guidelines.xhtml

Supplementary Table 4 | Factors associated with 28-day mortality using multivariable Cox proportional hazards model without a variable of the direct admission to the ICU

Variables	Died (n=779)	Survived (n=3027)	Adjusted hazard ratio (95%CI)	P value
**SFT group**[1]	205 (26%)	698 (23%)	0.77 (0.63-0.94)	0.01
**Male gender**	445 (57%)	698 (23%)	0.86 (0.74-1.00)	0.05
**Age group (years) (n [%])**				
• 18-40	59 (8%)	688 (23%)	1	<0.001
• >40-60	222 (29%)	930 (31%)	1.69 (1.26-2.26)	
• >60-70	159 (20%)	568 (19%)	2.07 (1.52-2.81)	
• >70	339 (44%)	841 (28%)	3.32 (2.50-4.41)	
**Transferred from other hospital**	715 (92%)	2595 (86%)	1.16 (0.89-1.52)	0.26
**Modified SOFA score (median, IQR)**	6 (4-9)	4 (3-6)	1.25 (1.22-1.27)	<0.001
**Comorbidities**				
• Diabetes mellitus	205 (26%)	602 (20%)	1.08 (0.91-1.27)	0.39
• Chronic kidney disease	141 (18%)	379 (13%)	1.20 (0.99-1.45)	0.07
• Liver disease	39 (5%)	85 (3%)	1.24 (0.89-1.72)	0.20
• Malignancy	24 (3%)	36 (1%)	2.52 (1.67-3.81)	<0.001
**Blood culture positive for pathogenic organisms**	190 (24%)	332 (11%)	1.83 (1.54-2.16)	<0.001
**Year**				
• 2013	165 (21%)	637 (21%)	1	0.34
• 2014	183 (23%)	651 (22%)	0.98 (0.79-1.21)	
• 2015	207 (27%)	808 (27%)	1.01 (0.81-1.26)	
• 2016[2]	224 (29%)	931 (31%)	0.85 (0.68-1.07)	

[1] Enrolled in the Sepsis Fast Track (SFT) programme
[2] Included 28 patients in 2017

For peer review only

For peer review only - http://bmjopen.bmj.com/site/about/guidelines.xhtml

Supplementary Table 5 | Factors associated with 28-day mortality using logistic multivariable model

Variables	Died (n=779)	Survived (n=3027)	Adjusted odds ratio (95%CI)	P value
**SFT group**[1]	205 (26%)	698 (23%)	0.61 (0.48-0.79)	<0.001
**Male gender**	445 (57%)	698 (23%)	0.88 (0.73-1.06)	0.18
**Age group (years) (n [%])**				
• 18-40	59 (8%)	688 (23%)	1	<0.001
• >40-60	222 (29%)	930 (31%)	1.91 (1.36-2.66)	
• >60-70	159 (20%)	568 (19%)	2.54 (1.78-3.63)	
• >70	339 (44%)	841 (28%)	4.76 (3.43-6.59)	
**Transferred from other hospital**	715 (92%)	2595 (86%)	1.21 (0.89-1.65)	0.23
**Modified SOFA score (median, IQR)**	6 (4-9)	4 (3-6)	1.30 (1.26-1.34)	<0.001
**Comorbidities**				
• Diabetes mellitus	205 (26%)	602 (20%)	1.12 (0.91-1.39)	0.29
• Chronic kidney disease	141 (18%)	379 (13%)	1.20 (0.94-1.53)	0.13
• Liver disease	39 (5%)	85 (3%)	1.34 (0.85-2.10)	0.21
• Malignancy	24 (3%)	36 (1%)	3.65 (2.05-6.51)	<0.001
**Blood culture positive for pathogenic organisms**	190 (24%)	332 (11%)	2.08 (1.66-2.61)	<0.001
**Year**				
• 2013	165 (21%)	637 (21%)	1	0.64
• 2014	183 (23%)	651 (22%)	1.05 (0.80-1.37)	
• 2015	207 (27%)	808 (27%)	1.05 (0.80-1.38)	
• 2016[2]	224 (29%)	931 (31%)	0.91 (0.69-1.21)	
**Admitted directly to an ICU upon admission**	128 (16%)	170 (6%)	1.95 (1.45-2.62)	<0.001

[1] Enrolled in the Sepsis Fast Track (SFT) programme
[2] Included 28 patients in 2017

For peer review only - http://bmjopen.bmj.com/site/about/guidelines.xhtml

Supplementary Table 6 | Factors associated with 28-day mortality using logistic multivariable model without a variable of the direct admission to the ICU

Variables	Died (n=779)	Survived (n=3027)	Adjusted odds ratio (95%CI)	P value
**SFT group[1]**	205 (26%)	698 (23%)	0.67 (0.53-0.86)	0.002
**Male gender**	445 (57%)	698 (23%)	0.87 (0.73-1.05)	0.14
**Age group (years) (n [%])**				
• 18-40	59 (8%)	688 (23%)	1	<0.001
• >40-60	222 (29%)	930 (31%)	1.85 (1.33-2.58)	
• >60-70	159 (20%)	568 (19%)	2.44 (1.71-3.46)	
• >70	339 (44%)	841 (28%)	4.55 (3.29-6.28)	
**Transferred from other hospital**	715 (92%)	2595 (86%)	1.23 (0.90-1.68)	0.19
**Modified SOFA score (median, IQR)**	6 (4-9)	4 (3-6)	1.32 (1.28-1.36)	<0.001
**Comorbidities**				
• Diabetes mellitus	205 (26%)	602 (20%)	1.16 (0.94-1.43)	0.17
• Chronic kidney disease	141 (18%)	379 (13%)	1.17 (0.92-1.49)	0.20
• Liver disease	39 (5%)	85 (3%)	1.31 (0.84-2.06)	0.23
• Malignancy	24 (3%)	36 (1%)	3.49 (1.95-6.24)	<0.001
**Blood culture positive for pathogenic organisms**	190 (24%)	332 (11%)	2.08 (1.67-2.61)	<0.001
**Year**				
• 2013	165 (21%)	637 (21%)	1	0.70
• 2014	183 (23%)	651 (22%)	1.00 (0.77-1.31)	
• 2015	207 (27%)	808 (27%)	1.01 (0.77-1.33)	
• 2016[2]	224 (29%)	931 (31%)	0.89 (0.67-1.17)	

[1] Enrolled in the Sepsis Fast Track (SFT) programme
[2] Included 28 patients in 2017

For peer review only

For peer review only - http://bmjopen.bmj.com/site/about/guidelines.xhtml

Supplementary Table 7 | Factors associated with 28-day mortality using multivariable Cox proportional hazards model in 2,170 patients enrolled into the study after the implementation of the Sepsis Fast Track (SFT) programme

Variables	Died (n=431)	Survived (n=1739)	Adjusted hazard ratio (95%CI)	P value
**SFT group**	205 (48%)	698 (40%)	0.68 (0.55-0.84)	<0.001
**Male gender**	254(59%)	992 (57%)	0.89 (0.73-1.09)	0.26
**Age group (years) (n [%])**				
• 18-40	30 (7%)	363(21%)	1	<0.001
• >40-60	125 (29%)	502 (29%)	1.97 (1.31-2.95)	
• >60-70	85 (20%)	359 (21%)	1.97 (1.28-3.03)	
• >70	191 (44%)	515 (30%)	3.33 (2.24-4.95)	
**Transferred from other hospital**	406 (94%)	1513 (87%)	1.49 (0.99-2.26)	0.06
**Modified SOFA score (median, IQR)**	7 (5-10)	4 (3-6)	1.24 (1.21-1.28)	<0.001
**Comorbidities**				
• Diabetes mellitus	122 (28%)	377(22%)	1.08 (0.87-1.35)	0.49
• Chronic kidney disease	84 (19%)	242 (14%)	1.23 (0.96-1.58)	0.11
• Liver disease	22 (5%)	57 (3%)	1.10 (0.71-1.70)	0.68
• Malignancy	19 (4%)	27 (2%)	2.90 (1.81-4.63)	<0.001
**Blood culture positive for pathogenic organisms**	110(26%)	225 (13%)	1.64 (1.31-2.05)	<0.001
**Year**				
• 2015	207 (48%)	808 (46%)	1	0.08
• 2016	224 (52%)	931 (54%)	0.84 (0.69-1.02)	
**Direct admission to the ICU**	85 (20%)	124 (7%)	1.78 (1.37-2.32)	<0.001

For peer review only

For peer review only - http://bmjopen.bmj.com/site/about/guidelines.xhtml

Supplementary Figure 1 | Preprinted recommended doctor orders for sepsis fast track programme used at the Emergency Department at Sunpasitthiprasong Hospital from 1 January 2015

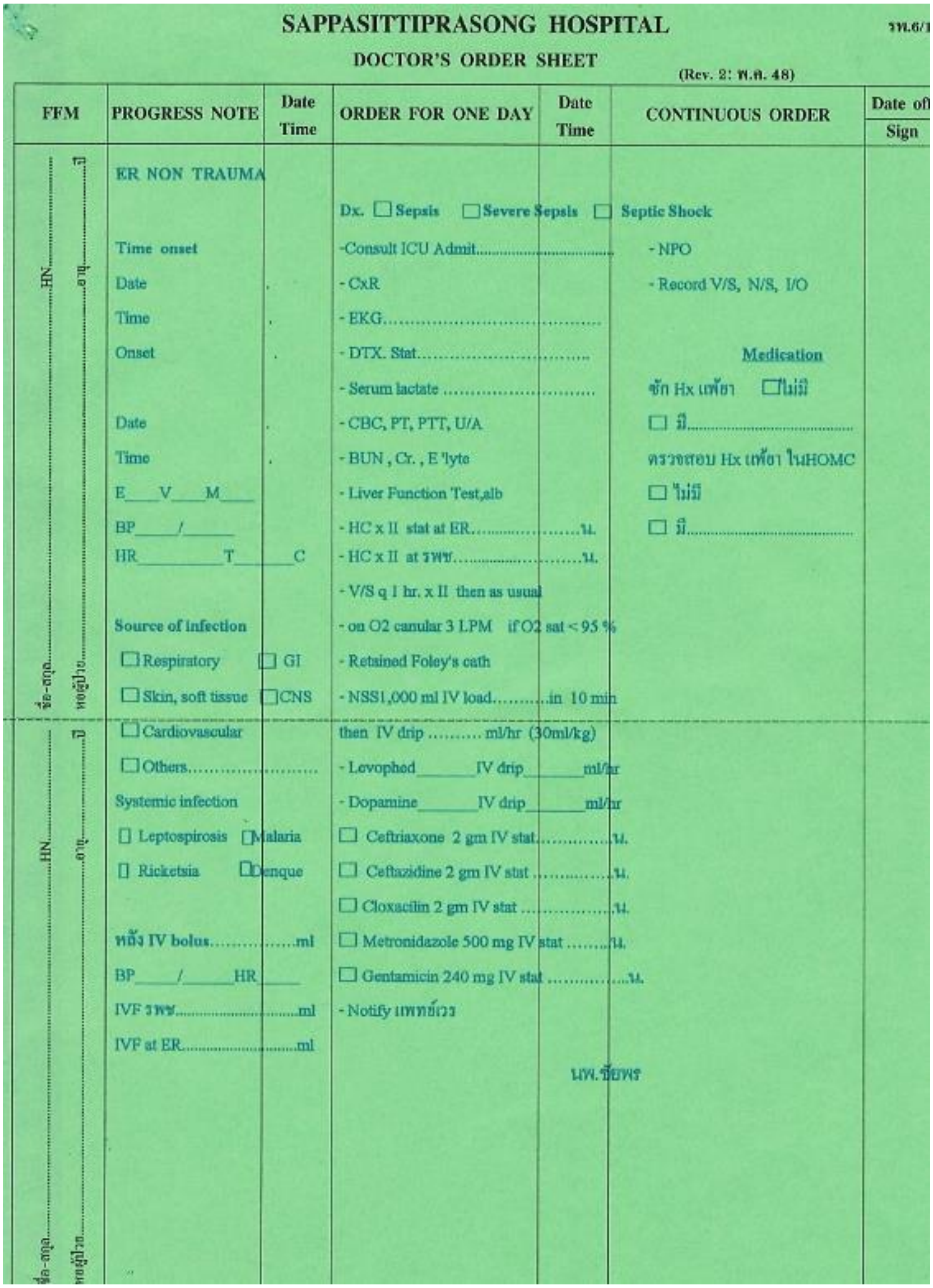
SAPPASITTIPRASONG HOSPITAL รพ.6/1

DOCTOR'S ORDER SHEET (Rev. 2: พ.ศ. 48)

FFM	PROGRESS NOTE	Date Time	ORDER FOR ONE DAY	Date Time	CONTINUOUS ORDER	Date off Sign
HN......... อายุ......... ปี	ER NON TRAUMA					
			Dx. ☐ Sepsis ☐ Severe Sepsis ☐ Septic Shock			
	Time onset		-Consult ICU Admit..........		- NPO	
	Date		- CxR		- Record V/S, N/S, I/O	
	Time		- EKG..........			
	Onset		- DTX. Stat..........		Medication	
			- Serum lactate		ซัก Hx แพ้ยา ☐ไม่มี	
	Date		- CBC, PT, PTT, U/A		☐ มี..........	
	Time		- BUN , Cr. , E 'lyte		ตรวจสอบ Hx แพ้ยา ในHOMC	
	E___V___M___		- Liver Function Test,alb		☐ ไม่มี	
	BP____/_____		- HC x II stat at ER..........น.		☐ มี..........	
	HR_______T_______C		- HC x II at รพช..........น.			
			- V/S q 1 hr. x II then as usual			
	Source of infection		- on O2 canular 3 LPM if O2 sat < 95 %			
	☐ Respiratory ☐ GI		- Retained Foley's cath			
ชื่อ-สกุล......... หอผู้ป่วย.........	☐ Skin, soft tissue ☐CNS		- NSS1,000 ml IV load..........in 10 min			
HN......... อายุ......... ปี	☐ Cardiovascular		then IV drip .... ml/hr (30ml/kg)			
	☐ Others..........		- Levophed______IV drip______ml/hr			
	Systemic infection		- Dopamine______IV drip______ml/hr			
	☐ Leptospirosis ☐Malaria		☐ Ceftriaxone 2 gm IV stat..........น.			
	☐ Ricketsia ☐Denque		☐ Ceftazidine 2 gm IV stat ....น.			
			☐ Cloxacilin 2 gm IV stat ....น.			
	หลัง IV bolus..........ml		☐ Metronidazole 500 mg IV stat ....น.			
	BP____/_____HR_____		☐ Gentamicin 240 mg IV stat ....น.			
	IVF รพช..........ml		- Notify แพทย์เวร			
	IVF at ER..........ml					
			นพ.ชัยพร			
ชื่อ-สกุล......... หอผู้ป่วย.........						

For peer review only - http://bmjopen.bmj.com/site/about/guidelines.xhtml

118 **Supplementary Figure 2 | The Sepsis Fast Track sepsis resuscitation workflow used at the**
119 **Emergency Department at Sunpasitthiprasong Hospital from 1 March 2016**

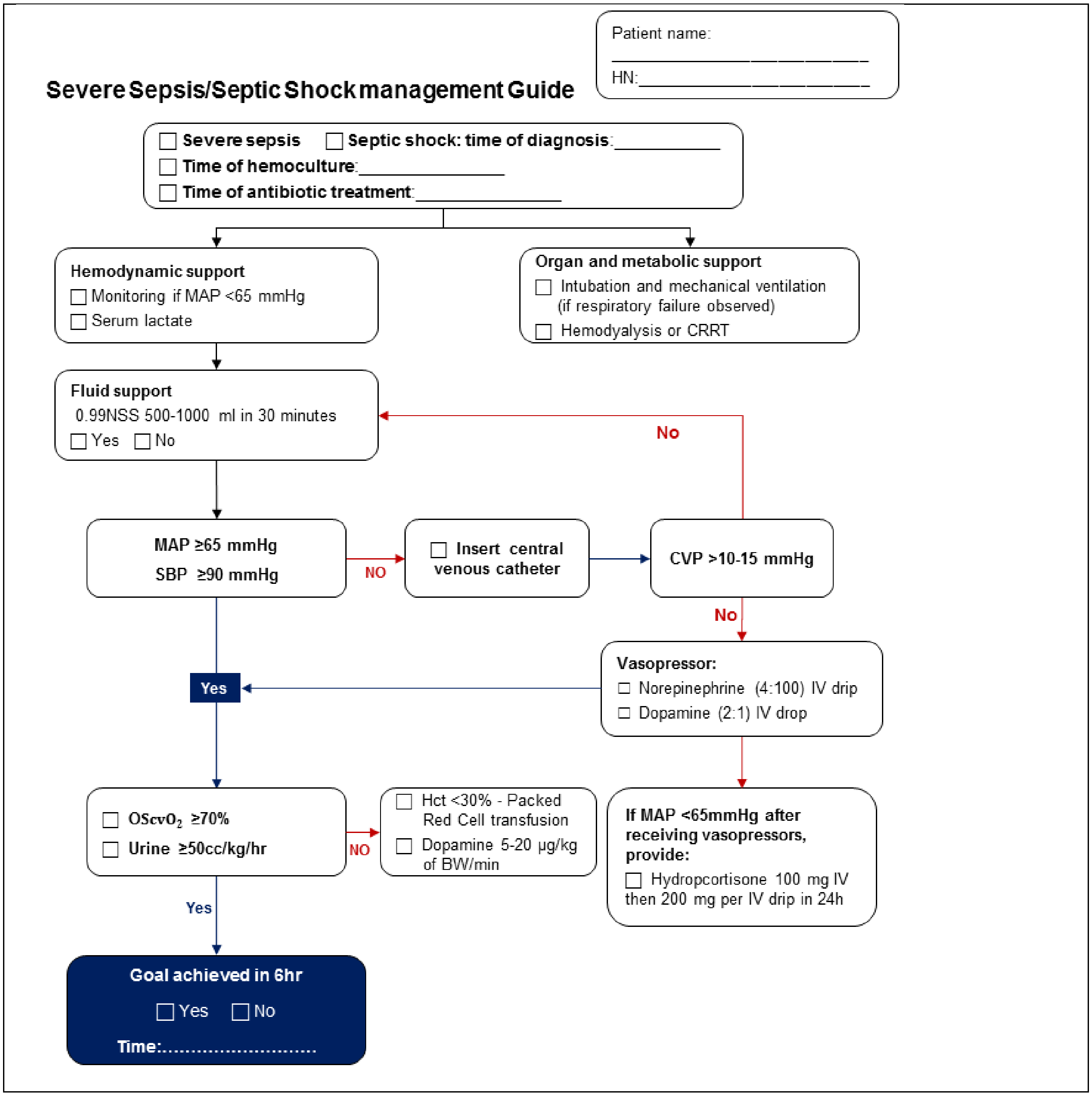

120
121
122

For peer review only - http://bmjopen.bmj.com/site/about/guidelines.xhtml

STROBE Statement

Checklist of items that should be included in reports of observational studies

Section/Topic	Item No	Recommendation	Reported on Page No
**Title and abstract**	1	(*a*) Indicate the study's design with a commonly used term in the title or the abstract	1, 3-4
		(*b*) Provide in the abstract an informative and balanced summary of what was done and what was found	3-4
**Introduction**			
Background/rationale	2	Explain the scientific background and rationale for the investigation being reported	5-6
Objectives	3	State specific objectives, including any prespecified hypotheses	6
**Methods**			
Study design	4	Present key elements of study design early in the paper	6-7
Setting	5	Describe the setting, locations, and relevant dates, including periods of recruitment, exposure, follow-up, and data collection	6-7
Participants	6	(*a*) *Cohort study*—Give the eligibility criteria, and the sources and methods of selection of participants. Describe methods of follow-up<br>*Case-control study*—Give the eligibility criteria, and the sources and methods of case ascertainment and control selection. Give the rationale for the choice of cases and controls<br>*Cross-sectional study*—Give the eligibility criteria, and the sources and methods of selection of participants	8-9
		(*b*) *Cohort study*—For matched studies, give matching criteria and number of exposed and unexposed<br>*Case-control study*—For matched studies, give matching criteria and the number of controls per case	
Variables	7	Clearly define all outcomes, exposures, predictors, potential confounders, and effect modifiers. Give diagnostic criteria, if applicable	10-11
Data sources/measurement	8*	For each variable of interest, give sources of data and details of methods of assessment (measurement). Describe comparability of assessment methods if there is more than one group	10-11
Bias	9	Describe any efforts to address potential sources of bias	11-12
Study size	10	Explain how the study size was arrived at	10-11
Quantitative variables	11	Explain how quantitative variables were handled in the analyses. If applicable, describe which groupings were chosen and why	11-12
Statistical methods	12	(*a*) Describe all statistical methods, including those used to control for confounding	11-12
		(*b*) Describe any methods used to examine subgroups and interactions	11-12
		(*c*) Explain how missing data were addressed	8
		(*d*) *Cohort study*—If applicable, explain how loss to follow-up was addressed<br>*Case-control study*—If applicable, explain how matching of cases and controls was addressed<br>*Cross-sectional study*—If applicable, describe analytical methods taking account of sampling strategy	6
		(*e*) Describe any sensitivity analyses	12

For peer review only - http://bmjopen.bmj.com/site/about/guidelines.xhtml

Section/Topic	Item No	Recommendation	Reported on Page No
**Results**			
Participants	13*	(a) Report numbers of individuals at each stage of study—eg numbers potentially eligible, examined for eligibility, confirmed eligible, included in the study, completing follow-up, and analysed	13
		(b) Give reasons for non-participation at each stage	
		(c) Consider use of a flow diagram	
Descriptive data	14*	(a) Give characteristics of study participants (eg demographic, clinical, social) and information on exposures and potential confounders	13-14
		(b) Indicate number of participants with missing data for each variable of interest	
		(c) *Cohort study*—Summarise follow-up time (eg, average and total amount)	
Outcome data	15*	*Cohort study*—Report numbers of outcome events or summary measures over time	16-17
		Case-control study—Report numbers in each exposure category, or summary measures of exposure	
		Cross-sectional study—Report numbers of outcome events or summary measures	
Main results	16	(*a*) Give unadjusted estimates and, if applicable, confounder-adjusted estimates and their precision (eg, 95% confidence interval). Make clear which confounders were adjusted for and why they were included	13-17
		(*b*) Report category boundaries when continuous variables were categorized	
		(*c*) If relevant, consider translating estimates of relative risk into absolute risk for a meaningful time period	
Other analyses	17	Report other analyses done—eg analyses of subgroups and interactions, and sensitivity analyses	16-17
**Discussion**			
Key results	18	Summarise key results with reference to study objectives	18-22
Limitations	19	Discuss limitations of the study, taking into account sources of potential bias or imprecision. Discuss both direction and magnitude of any potential bias	20-21
Interpretation	20	Give a cautious overall interpretation of results considering objectives, limitations, multiplicity of analyses, results from similar studies, and other relevant evidence	20-21
Generalisability	21	Discuss the generalisability (external validity) of the study results	21-22
**Other Information**			
Funding	22	Give the source of funding and the role of the funders for the present study and, if applicable, for the original study on which the present article is based	23

**Give information separately for cases and controls in case-control studies and, if applicable, for exposed and unexposed groups in cohort and cross-sectional studies.*

Note: An Explanation and Elaboration article discusses each checklist item and gives methodological background and published examples of transparent reporting. The STROBE checklist is best used in conjunction with this article (freely available on the Web sites of PLoS Medicine at http://www.plosmedicine.org/, Annals of Internal Medicine at http://www.annals.org/, and Epidemiology at http://www.epidem.com/). Information on the STROBE Initiative is available at www.strobe-statement.org.

For peer review only - http://bmjopen.bmj.com/site/about/guidelines.xhtml
